# Controlled Molecular Orders in Layered Multiple Porphyrins

**DOI:** 10.1021/jacs.5c07484

**Published:** 2025-08-22

**Authors:** Tomoki Kodama, Naoyuki Hisano, Shin-ichi Tate, Takeharu Haino

**Affiliations:** § Department of Chemistry, Graduate School of Advanced Science and Engineering, 124684 Hiroshima University, 1-3-1 Kagamiyama, Higashi-Hiroshima, Hiroshima 739-8526, Japan; ¶ International Institute for Sustainability with Knotted Chiral Meta Matter (WPI-SKCM2), Hiroshima University, 1-3-1 Kagamiyama, Higashi-Hiroshima, Hiroshima 739-8526, Japan; ∥ Department of Mathematical and Life Sciences, Graduate School of Integrated Sciences for Life, 124684 Hiroshima University, 1-3-1, Kagamiyama, Higashi-Hiroshima 739-8526, Japan

## Abstract

Nature precisely regulates multicomponent assemblies with the assistance of cooperativity. However, establishing such high precision in multicomponent assemblies of artificial supramolecular structures remains challenging. Here, we successfully position multiple distinct guest molecules within two equivalent binding cavities of a zinc-metalated trisporphyrin host by combining two distinct negative cooperative interactions, including donor–acceptor π-stacking and metal–ligand coordination. Comprehensive characterization using UV–vis absorption spectroscopy and diffusion-ordered NMR spectroscopy confirmed the exclusive formation of a ternary supramolecular complex. X-ray crystallographic analysis further revealed that the introduction of an additional bridging ligand effectively linked the two ternary complexes to produce an unprecedented septenary supramolecular assembly with alternating guest sequences. In contrast to conventional methods, which require structural differentiation or positive cooperativity, our strategy relies exclusively on negative cooperativity to achieve highly precise molecular ordering. This study presents a novel approach toward constructing sophisticated multicomponent molecular assemblies, emphasizing the significant but underutilized role of negative cooperativity in achieving molecular precision in artificial supramolecular chemistry.

## Graphical abstract

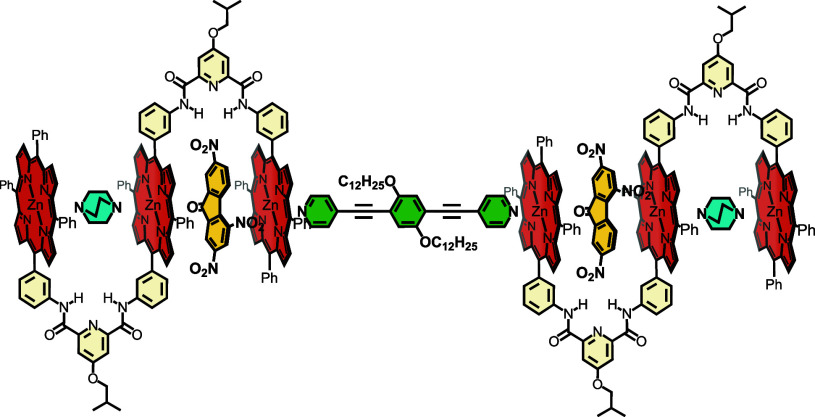

## Introduction

Molecular tweezers are open-ended, cleft-shaped host molecules composed of two parallel aromatic ″arms″ that form a cavity for binding guests.^1–5^ These hosts have attracted broad interest in areas such as small-molecule recognition, supramolecular polymers, sensors, and molecular machines.^6–9^ Guest encapsulation within cleft cavities is driven by noncovalent interactions such as donor–acceptor, π–π stacking, and CH/π; therefore, electron-rich or electron-deficient aromatic rings have often been exploited to gain sufficient binding energy for host–guest complexation.^10–36^

Extending a molecular tweezer by stacking additional aromatic units in a layer-by-layer fashion yields a host with multiple identical clefts, enabling the binding of multiple guests simultaneously.^5^ Porphyrin-based systems are prominent examples: folded porphyrin oligomers can accommodate several guest molecules (e.g., fullerenes, diamines), and multidecker metallo-″tweezers″ have been shown to intercalate planar aromatic guests in layered arrays.^37–40^ Two mononuclear planar platinum complexes were intercalated within double-decker tweezers layer by layer.^41,42^ However, guest binding in these multicleft hosts is usually noncooperative; each cavity acts independently. As a result, mixtures of different guests tend to be distributed randomly among equivalent sites rather than in a defined sequence. For example, a polymer host with repeating aromatic clefts can fold around pyrene to form a perfectly ordered one-dimensional array of identical guests, but introducing a second aromatic guest most likely leads to statistical (random) occupancy instead of ordered alternation.^43–48^ This limitation calls for a strategy to bias the occupancy of each site and thereby achieve an ordered heteroguest arrangement in multicavity hosts.

One promising mechanism is the homotropic negative cooperative binding of a guest at one site that significantly diminishes the affinity of an identical site for the same guest. Unlike positive cooperative binding, wherein the binding of one guest enhances the affinity for subsequent identical guests and promotes homotropic guest occupation, negative cooperativity enables selective heteroguest arrangement in a multicleft host. If a two-cleft host exhibits strong negative cooperativity for two different guests, A and B, with each guest binding via an orthogonal interaction, and then the binding of guest A in one cleft will prevent a second A from binding, effectively favoring guest B in the remaining cleft. However, realizing such behavior is exceptionally challenging because it requires each guest-binding event to allosterically inhibit further binding of the same kind while remaining compatible with the other guest-binding event.^49,50^

In this work, a tailor-made triple-layered porphyrin host **1** with two identical clefts is used to achieve this selective binding behavior (Figure 1).^51^ In its free-base form, **1** binds one electron-deficient aromatic guest **G1** via donor–acceptor π-stacking;^52^ importantly, that first binding event reduces the electron density of the central porphyrin and prevents a second **G1** from binding in the other cleft; thus, an exclusive 1:1 complex **G1**•**1** is obtained, demonstrating strong negative cooperativity. To enable a different guest to occupy the remaining site, **1** was metalated with Zn­(II) to obtain **1Zn**. Metalation activates the empty cleft to bind a divalent guest such as 1,4-diazabicyclo[2.2.2]­octane (**G2**) via axial coordination to the Zn centers. This coordination is orthogonal to the π-stacking mode and also exhibits negative cooperativity, where each **1Zn** host binds only one **G2** due to the pentacoordinate geometry of its Zn centers.^53–57^ Thus, in the presence of both guests, zinc trisporphyrin selectively forms a ternary complex, **G1**•**1Zn**•**G2**, with one of each guest in its two clefts. Moreover, by introducing additional ditopic guests as bridges, these ternary complexes could be connected to form higher-order assemblies with precisely ordered alternating guest sequences (Scheme 1). To our knowledge, this work provides the first example of a multicleft host that can direct the orderly coencapsulation of two different guests through purely negative cooperative interactions operating across two spatially separated yet electronically communicated cleft cavities, thereby offering a new design strategy for sequence-controlled multicomponent supramolecular systems.

**Figure 1:**
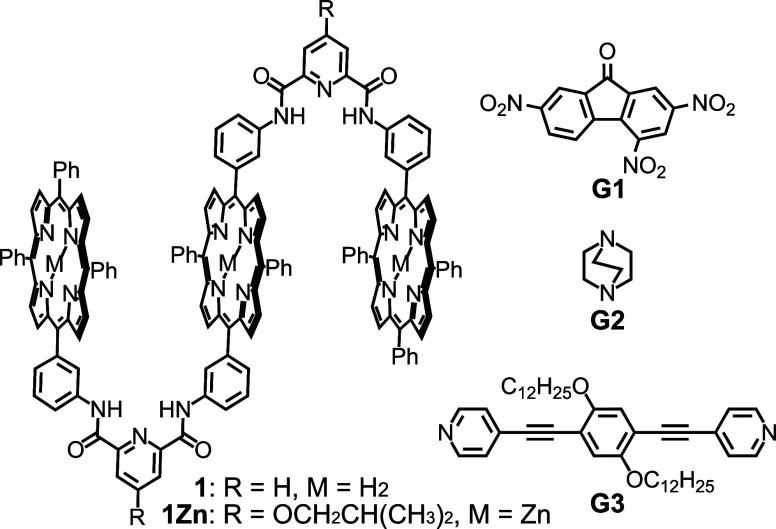
Structures of trisporphyrin **1** and **1Zn** and guest molecules **G1**, **G2**, and **G3**.

**Scheme 1:**
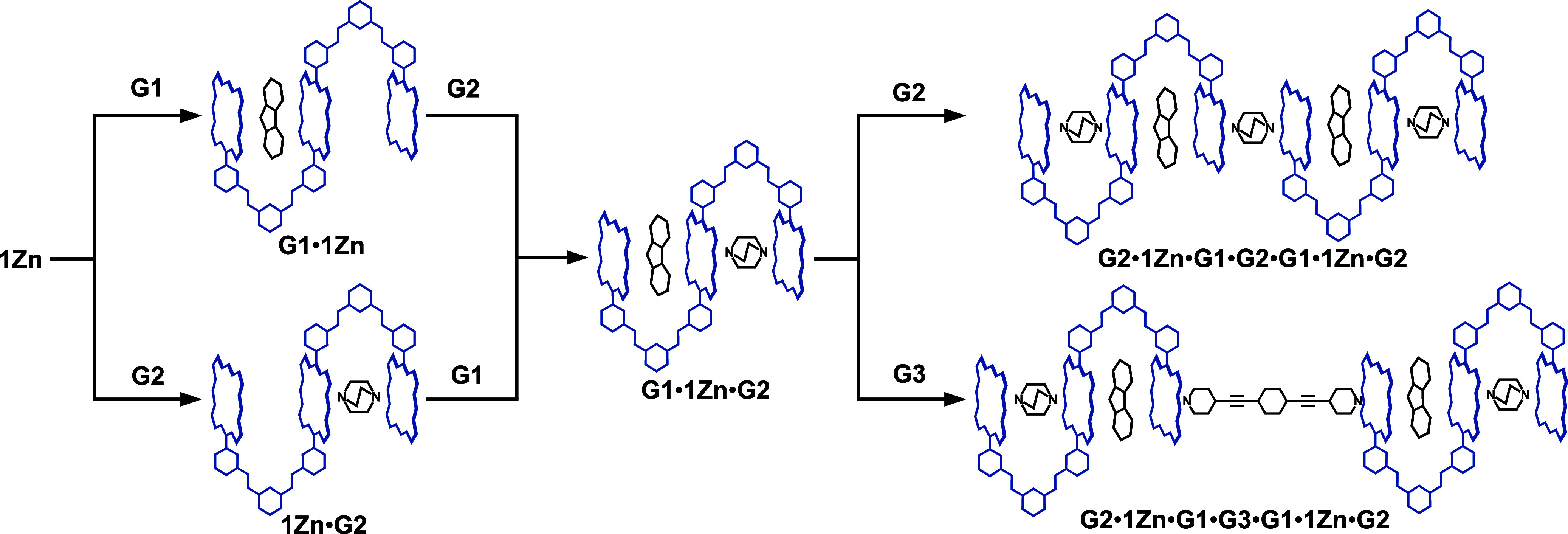
Multistep Host–Guest Complexation of **1Zn** with **G1**, **G2**, and **G3**

## Results and Discussion

Self-sorting of the ternary complex **G1**•**1Zn**•**G2** requires perfect homotropic negative cooperativity in the guest-binding behavior of both **G1** and **G2** to **1Zn**. Therefore, each guest binding behavior was independently assessed to verify the negative cooperativity in the host–guest complexation prior to studying the ternary complex formation in solution. The UV–vis absorption spectrum of **1Zn** yielded the Soret band at 422 nm, which was sensitive to the host–guest complexation. Upon the addition of **G1**, the Soret band gradually diminished, and a new absorption band emerged at approximately 440 nm with an isosbestic point at 432 nm (Figure 2a).^58^ The symmetric Job plot exhibited a peak at a host molar fraction of 0.5, indicating that the host–guest complex **1Zn**•**G1** was exclusively formed in a ratio of 1:1 (Figure 2b), although **1Zn** has two identical cleft cavities; thus, the host–guest complexation between **1Zn** and **G1** exhibits strong homotropic negative cooperativity, where the first guest binding substantially reduces the electron density of the central porphyrin ring, remarkably impeding the successive guest binding into the resting cleft cavity.^59^ The plots of the absorption changes versus the guest concentrations were well fitted by nonlinear regression analysis using the 1:1 binding model (Figure S11). The binding constant of **1Zn** and **G1** was estimated to be 9.34(5) × 10^5^ L mol^–1^. The host–guest complexation between **1Zn** and **G2** was examined in the same manner. The addition of **G2** reduced the intensity of the Soret band at 422 nm, while a sharp absorption band at 424 nm with isosbestic points at 422, 428, and 435 nm appeared simultaneously (Figure 2c).^60^ The Job plot with a peak at a host molar fraction of 0.5 determined the formation of a 1:1 host–guest ratio in the complexation between **1Zn** and **G2** (Figure 2d). Nonlinear regression analysis yielded a binding constant of 7.2(9) × 10^7^ L mol^–1^ for the host–guest complex **1Zn**•**G2** (Figure S11).

**Figure 2:**
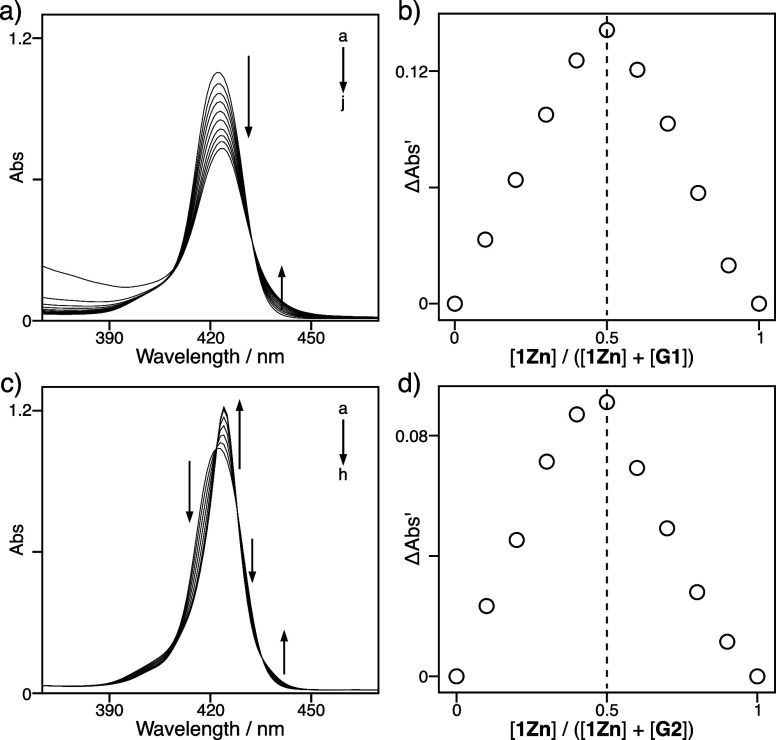
UV–vis absorption spectra of **1Zn** (8.1 × 10^–7^ mol L^–1^) with (a) **G1** (a–j: 0.0, 3.1, 6.5, 10, 16, 24, 40, 65, 120, and 400 × 10^–7^ mol L^–1^) and (c) **G2** (a–h: 0.0, 1.6, 3.3, 4.9, 6.5, 8.1, 16, and 24 × 10^–7^ mol L^–1^) at 295 K in chloroform. Job plot **1Zn** with (b) **G1** and (d) **G2** at 295 K in chloroform. The total concentration of a mixture of host and guest was maintained at a constant value (**1Zn** + **G1**: 2.0 × 10^–4^ mol L^–1^; **1Zn** + **G2**: 2.0 × 10^–4^ mol L^–1^). ΔAbs′ indicates |Abs – Abs_H_·*X* – Abs_G_·*X*|, where Abs, Abs_H_, and Abs_G_ indicate the observed absorbance, absorbance of the host, and absorbance of the guest, respectively.

The host–guest complexation of **G1** and **1Zn** is orthogonal to that of **G2** due to the strong negative cooperativity. Therefore, both the host–guest complexes, **1Zn**•**G1** and **1Zn**•**G2**, have resting cleft cavities that are capable of encapsulating **G2** and **G1**, respectively, resulting in the ternary host–guest complex **G1**•**1Zn**•**G2** (Scheme 1). To assess the binding ability of the resting cleft cavities of **1Zn**•**G1** and **1Zn**•**G2**, stepwise titration experiments of **1Zn** with **G1** and **G2** were performed at a micromolar concentration of **1Zn** (2.0 × 10^–5^ mol L^–1^), which allowed the absorption change at the *Q* bands to be easily followed upon the addition of **G1** and **G2** (Figure 3). The addition of **G1** to a solution of **1Zn** reduced the intensity of the *Q*-band at 552 nm, and new broad bands emerged at approximately 500, 580, and 620 nm (Figure 3a). The conversion to **1Zn**•**G1** was completed by adding 15 equiv of **G1**. Subsequently, the addition of **G2** to the resulting solution decreased the intensity of the *Q*-band at 552 nm, and new absorption bands appeared at 561 and 601 nm, with isosbestic points at 559, 581, and 593 nm (Figure 3b). In contrast, the addition of **G2** to a solution of **1Zn** resulted in band shifts from 552 to 561 nm and 594 to 601 nm (Figure 3c). The shifts were completed after the addition of 15 equiv of **G2**. The successive addition of **G1** to the solution reduced the new *Q* bands at 561 and 601 nm, and new, broad absorption bands emerged at approximately 530, 585, and 630 nm (Figure 3d). Eventually, the *Q*-band spectrum obtained from the former titration experiment was consistent with that obtained from the latter, which was visualized by plotting the absorbance at 563 nm versus the stoichiometries of **G1** and **G2** to **1Zn**, indicating perfectly independent two-step processes of host–guest complexation without guest scrambling (Figure 3e). Accordingly, the zinc trisporphyrin host **1Zn** exclusively formed the ternary host–guest complex **G1**•**1Zn**•**G2** even in the presence of an excess amount of **G1** and **G2** due to the strong negative cooperativity (Figures S16 and S17).

**Figure 3:**
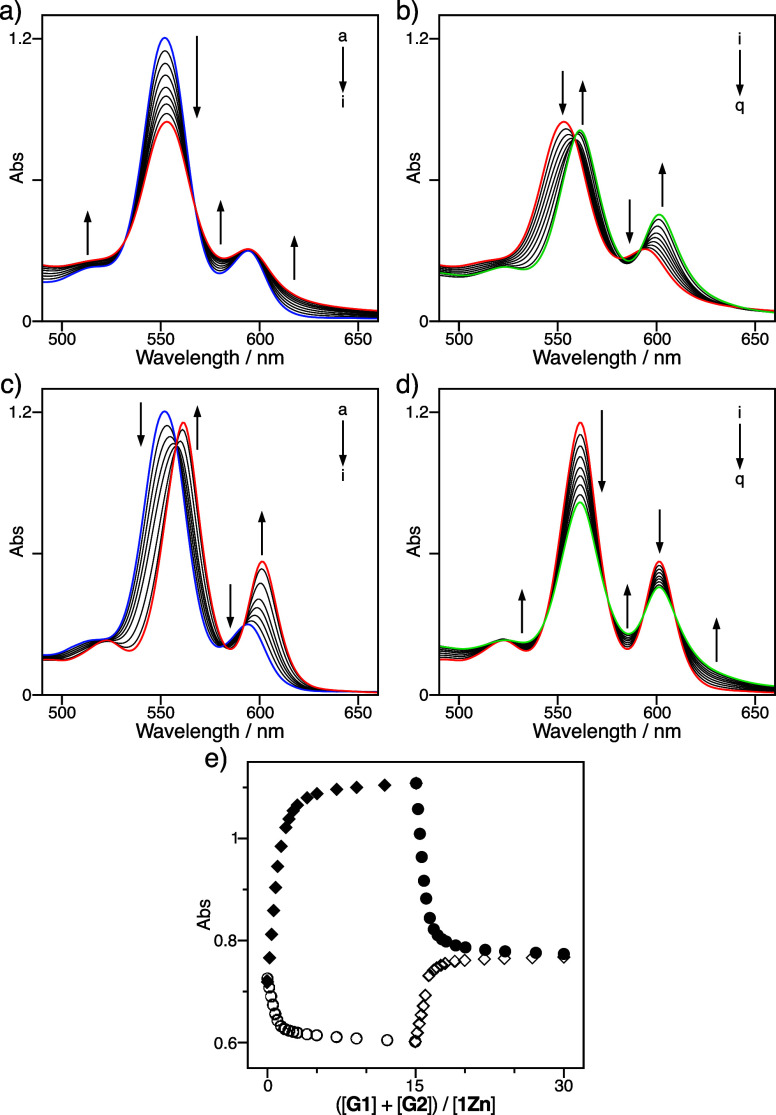
(a, b) UV–vis absorption spectral changes of **1Zn** (2.0 × 10^–5^ mol L^–1^) upon addition of **G1** (a–i: 0.0, 0.38, 0.77, 1.2, 1.6, 2.1, 2.8, 8.1, and 30 × 10^–5^ mol L^–1^) and then **G2** (i–q: 0.0, 0.42, 0.83, 1.2, 1.7, 2.1, 2.8, 5.3, and 30 × 10^–5^ mol L^–1^) at 295 K in chloroform. (c, d) UV–vis absorption spectral changes of **1Zn** (2.0 × 10^–5^ mol L^–1^) upon addition of **G2** (a–i: 0.0, 0.42, 0.83, 1.2, 1.7, 2.8, 3.8, 6.1, and 30 × 10^–5^ mol L^–1^) and then **G1** (i–q: 0.0, 0.38, 0.77, 1.2, 1.6, 2.1, 2.8, 5.3, and 30 × 10^–5^ mol L^–1^) at 295 K in chloroform. (e) Plots of Abs at 563 nm of **1Zn** versus ([**G1**] + [**G2**])/[**1Zn**] upon the stepwise addition of **G1** (circle) and **G2** (rhombus).

The ternary complex formation of a host possessing two identical binding sites with two distinct guest molecules is governed either by heterotropic positive cooperativity, wherein the initial binding of one guest molecule enhances the affinity for subsequent guest binding, or by homotropic negative cooperativity, wherein the two distinct guest molecules independently exhibit negative cooperative binding without mutual influence. To discuss the cooperative effect in the host–guest complexation of **1Zn**, the binding constants of the resting cleft cavities of **1Zn**•**G1** and **1Zn**•**G2** were determined by titration experiments with **1Zn** in the presence of **G1** or **G2**. The binding constant of **1Zn** with **G1** was determined to be 2.60(2) × 10^5^ L mol^–1^ in the presence of 1 equiv of **G2** at a concentration of 8.1 × 10^–**7**^ mol L^–1^, where 88% of **1Zn** existed as **1Zn**•**G2** (Figure S12). The binding constant of **1Zn** and **G2** in the presence of 20 equiv of **G1** was determined to be 4.8(6) × 10^**7**^ L mol^–1^ at a concentration of 8.0 × 10^–7^ mol L^–1^, where 93% of **1Zn** existed as **1Zn**•**G1** (Figure S12). The binding ability of **1Zn** to **G1** and **G2** was not influenced by the presence of competitive guests **G2** and **G1**, respectively. Thus, the resting cleft cavities of **1Zn**•**G1** and **1Zn**•**G2** maintained binding powers comparable to those of **1Zn**.

The formation of the ternary host–guest complex **G1**•**1Zn**•**G2** was investigated using ^1^H NMR spectroscopy and diffusion-ordered NMR (DOSY) spectroscopy.^61,62^ DOSY experiments provided information about the size of the supramolecular complexes in solution. According to the Stokes–Einstein relation, there is an inversely proportional relationship between the diffusion coefficient (*D*) and hydrodynamic radius (*r*_h_) in solution. After 1.0 equiv each of **G1** and **G2** were added to a solution of **1Zn** at a concentration of 10 mmol L^–**1**^ in chloroform-*d*, the aromatic protons of **G1** and the methylene protons of **G2** showed large upfield shifts (Figure 4a–c). Aromatic protons Ha, Hb, Hc, Hd, and He appeared at 6.28, 5.77, 2.75, 5.28, and 4.61 ppm, respectively (Figure S18). Large complexation-induced shifts (CISs) of −2.41, −2.80, −5.62, −3.75, and −4.22 for Ha, Hb, Hc, Hd, and He, respectively, locate **G1** within the cleft cavity of **1Zn**, where the aromatic protons of **G1** experienced the strong shielding effect of the porphyrin rings. In the presence of **1Zn**, the chemically equivalent methylene protons Hf of **G2** became magnetically nonequivalent and split into two signals at −5.27 and −5.76 ppm. Large CISs of −8.06 and −8.55 ppm for the methylene protons Hf locate a **G2** molecule within the cavity of trisporphyrin **1Zn**. The upfield-shifted protons of **G1** and **G2** showed a diffusion coefficient of 2.76(2) × 10^–10^ m^2^ s^–1^ and 2.79(2) × 10^–10^ m^2^ s^–1^, respectively, which is consistent with those observed for the protons of **1Zn** (2.81(1) × 10^–10^ m^2^ s^–1^), thereby confirming the formation of the ternary complex **G1**•**1Zn**•**G2** in solution (Figure 4d and Figures S20 and S21).^63^ There is a resting coordination site on the outer porphyrin ring that interacts with **G2**. Upon the addition of 0.5 equiv excess of **G2**, the diffusion coefficient reached a minimum value of 2.32(2) × 10^–10^ m^2^ s^–1^ (Figure 4d and Figures S22 and S23), indicating that the multicomponent supramolecular complex **G2•1Zn•G1•G2•G1•1Zn•G2** was most likely formed. Beyond this point, the addition of more **G2** increased the diffusion coefficient, which disrupted the septenary complex into the quaternary complex **G2•G1•1Zn•G2**.^64^

**Figure 4:**
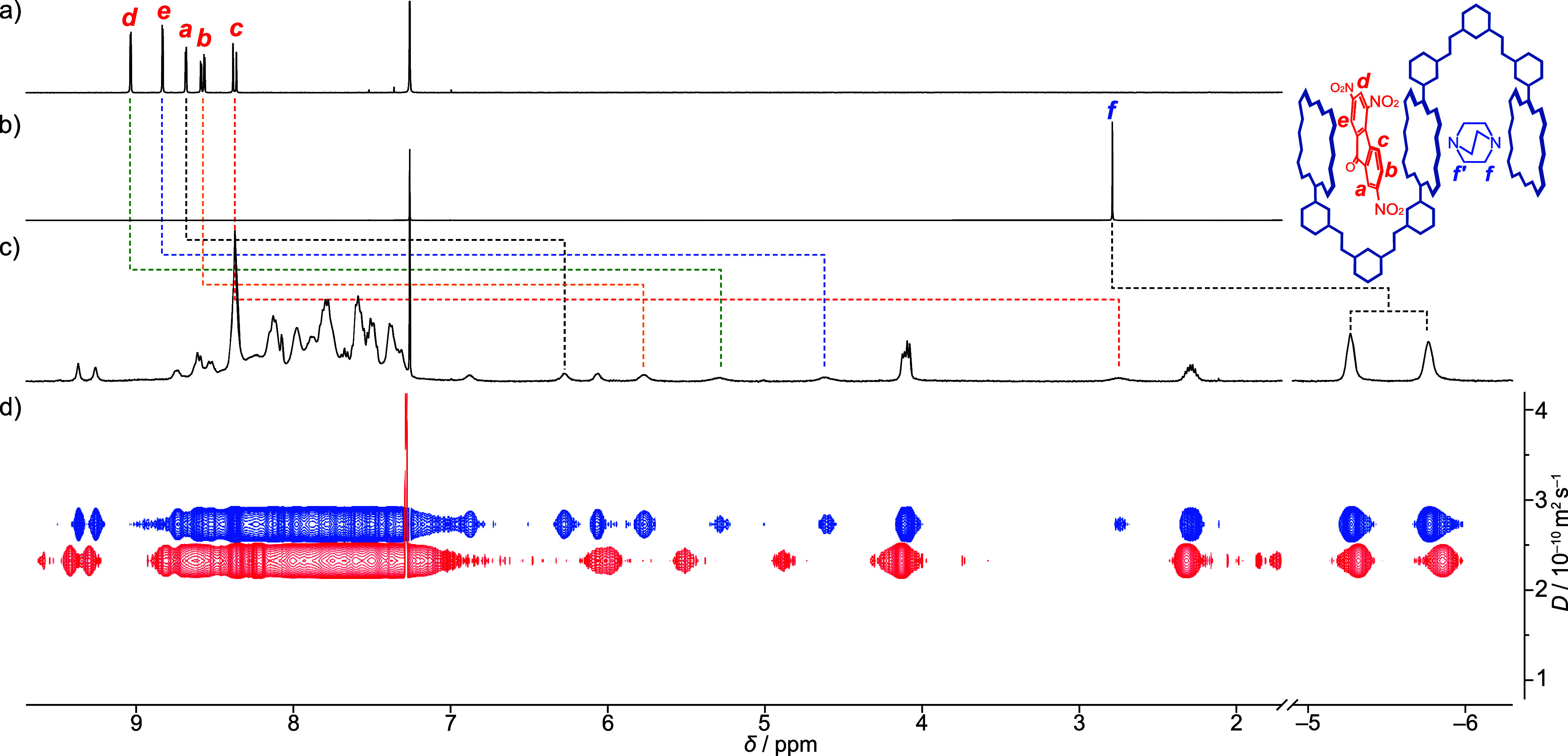
^1^H NMR spectra of (a) **G1**, (b) **G2**, and (c) a 1:1:1 mixture of **1Zn**, **G1**, and **G2**. (d) Diffusion coefficients of a 1:1:1 mixture of **1Zn**, **G1**, and **G2** (blue line) and a 1:1:1.5 mixture of **1Zn**, **G1**, and **G2** (red line).

A single crystal of trisporphyrin **1Zn** in the presence of **G1** and **G2** was grown in a monoclinic unit cell with space group *P*2_1_/*n* (#14) by liquid–liquid diffusion of hexane into a chloroform solution (Figure 5 and Figure S28). The crystal structure showed that **G1** and **G2** were encapsulated within the cavities of the host molecule **1Zn**. **G1** was accommodated in the bisporphyrin cleft cavity with an interlayer porphyrin distance of 6.55 Å, resulting from the π–π stacking interactions of the porphyrin rings and the **G1** molecule (Figure S29). The intermolecular N···O distances between the amide and nitro groups and the C···O distances between the phenyl, nitro, and carbonyl groups were in the range of 3.15–3.74 Å, indicating the presence of weak intermolecular hydrogen bonding interactions. The bisporphyrin cleft cavity encapsulated the **G2** molecules through the coordination interactions. The interlayer distance of the two porphyrin rings is fairly extended to 7.72 Å because **G2** generates the coordination interactions with the interatomic Zn···N distances of 2.17 and 2.20 Å. The unit cell contained a 2:2:3 ratio of **1Zn**, **G1**, and **G2**, where a **G2** molecule was located at the outer end of the bisporphyrin cleft that encapsulated **G1** to form the self-sorted septenary complex **G2**•**1Zn**•**G1**•**G2**•**G1**•**1Zn**•**G2**, where seven molecules of the three types were sorted in order.^65^

**Figure 5:**
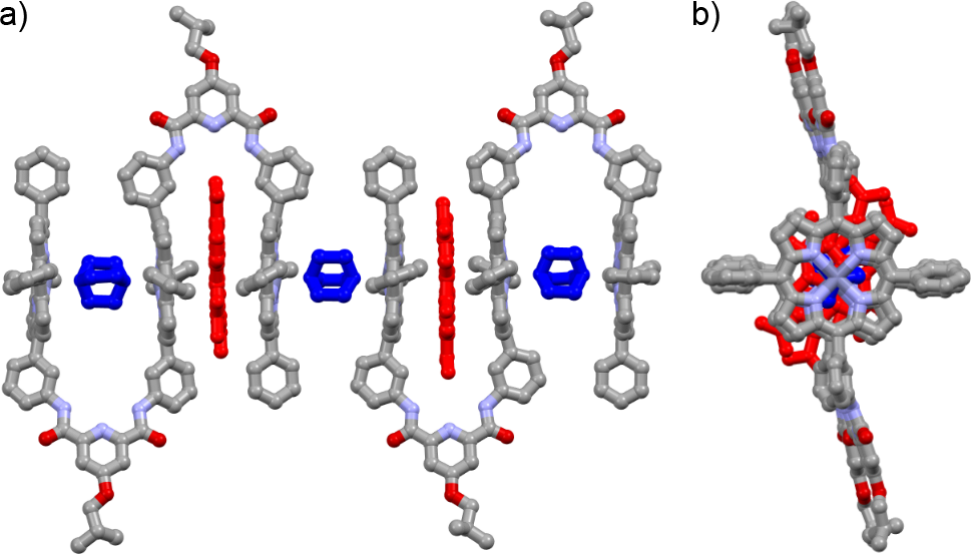
Crystal structures of **G2**•**1Zn**•**G1**•**G2**•**G1**•**1Zn**•G2 from (a) the side and (b) the top. Guest molecules **G1** and **G2** are shown in red and blue, respectively. All hydrogen atoms have been omitted for clarity.

This crystal structure inspired us to employ another divalent ligand **G3**, which is too long to be accommodated within the bisporphyrin cleft cavity. **G3** becomes a cross-linker that connects two of the ternary host–guest complexes **G1**•**1Zn**•**G2** to develop an unprecedented septenary complex, **G2**•**1Zn**•**G1**•**G3**•**G1**•**1Zn**•**G2**, where the seven molecules of the four types are sorted in that order (Scheme 1). When 0.5 equiv of **G3** was added to the solution of **G1**•**1Zn**•**G2**, aromatic protons Hg, Hh, and Hi of **G3** were upfield shifted and appeared at 1.82, 5.09, and 5.99 ppm with CISs of −6.78, −2.29, and −1.04 ppm, respectively (Figure 6a and Figures S30 and S31). The characteristic upfield shifts of Hg and Hh indicate that the pyridine ring is coordinated to the outer porphyrin ring, where the protons experience the strong shielding effect of the porphyrin ring. The diffusion coefficient of the ternary host–guest complex **G1**•**1Zn**•**G2** was estimated to be 2.81(1) × 10^–10^ m^2^ s^–1^. The addition of 0.5 equiv of **G3** to the solution decreased the diffusion coefficient to 1.92(1) × 10^–10^ m^2^ s^–1^ (Figure 6b and Figures S32 and S33), which suggests that the addition of **G3** increased the hydrodynamic volume of **G1**•**1Zn**•**G2** by roughly three times that before the addition. Based on these findings, the septenary supramolecular complex **G2**•**1Zn**•**G1**•**G3**•**G1**•**1Zn**•**G2** was established with a controlled molecular order directed by self-sorting behavior.

**Figure 6:**
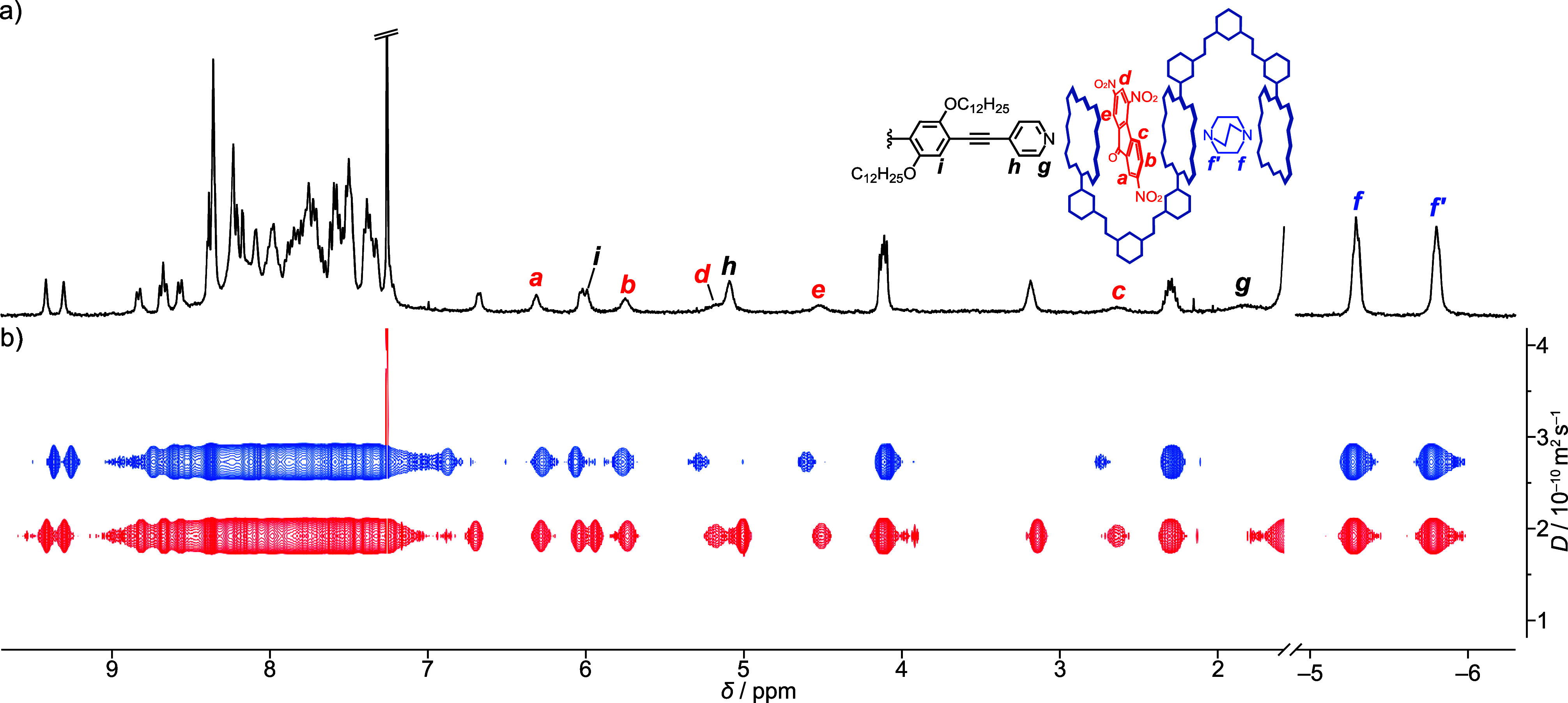
^1^H NMR spectra of (a) a 1:1:1:0.5 mixture of **1Zn**, **G1**, **G2**, and **G3**, and (b) diffusion coefficients of a 1:1:1 mixture of **1Zn**, **G1**, and **G2** (blue line) and a 1:1:1:0.5 mixture of **1Zn**, **G1**, **G2**, and **G3** (red line).

## Conclusions

In conclusion, we demonstrated that a mixture of an electron-deficient aromatic molecule, a ditopic donor ligand, and zinc trisporphyrin **1Zn** yielded the ternary host–guest complex **G1**•**1Zn**•**G2** through strong homotropic negative cooperativity. The structure of the ternary host–guest complex was determined in solution. X-ray analysis revealed that **G2** bridged two molecules of the ternary host–guest complex, **G1**•**1Zn**•**G2**. Guests **G2** and **G3** coordinated **G1**•**1Zn**•**G2** to form the unprecedented septenary supramolecular complexes **G2**•**1Zn**•**G1**•**G2**•**G1**•**1Zn**•**G2** and **G2**•**1Zn**•**G1**•**G3**•**G1**•**1Zn**•**G2**, respectively.

Nature makes extensive use of negative cooperativity in feedback regulation. However, negative cooperativity, as opposed to positive cooperativity, has not received much attention in supramolecular and host–guest chemistry. This example established unprecedented multiple molecular orders, reminding us of the vital value of negative cooperativity for the precise structural control of complex supramolecular structures, which may pave the way to providing clues for the design of far more complex supramolecular structures as nature does.

## Supplementary Material



## Notes

Full details of the synthesis, characterization, host–guest study, and crystallographic analysis (PDF)
