# Supplementary material for: Controlled Molecular Orders in Layered Multiple Porphyrins
Source: J Am Chem Soc. 2025 Aug 22;147(35):31671–8. doi: 10.1021/jacs.5c07484 (PMC12412108; doi:10.1021/jacs.5c07484)
Supplement: Supplementary file 1 [file ja5c07484_si_001.pdf]

Supporting Information for

Controlled Molecular Orders in Layered Multiple Porphyrins

Tomoki Kodama[§,¶], Naoyuki Hisano[§], Shin-ichi Tate[¶,∥] and Takeharu Haino*[,¶,§]

[§] *Department of Chemistry, Graduate School of Advanced Science and Engineering, Hiroshima University, 1-3-1, Kagamiyama, Higashi-Hiroshima 739-8526, Japan*

[¶] *International Institute for Sustainability with Knotted Chiral Meta Matter (WPI-SKCM²), Hiroshima University, 1-3-1 Kagamiyama, Higashi-Hiroshima, 739-8526 Japan*

[∥] *Department of Mathematical and Life Sciences, Graduate School of Integrated Sciences for Life, Hiroshima University, 1-3-1 Kagamiyama, Higashi-Hiroshima, Hiroshima, 739-8526 Japan*

*To whom correspondence should be addressed: E-mail: haino@hiroshima-u.ac.jp.

Table of contents

1 Experimental procedures S2

1.1 General methods S2

1.2 Synthesis of **1Zn** S3

1.3 Synthesis of **9Zn** S5

1.4 NMR and ESI spectra S6

2 Titration experiments and binding constants S15

2.1 UV-Vis studies of binding behavior of **1Zn** S15

2.2 UV-Vis studies of binding behavior of **9Zn** S19

2.3 FL studies of binding behavior of **1Zn** S20

3 Formation of ternary and septenary supramolecular complexes S21

3.1 NMR studies of ternary complex S21

3.2 DOSY experiments of ternary complex S22

3.3 ESI-MS study of ternary and septenary supramolecular complex S26

3.4 X-ray Crystallography S26

4 Formation of septenary supramolecular complex G2•1Zn•G1•G3•G1•1Zn•G2 S28

5 DFT calculations S30

6 References S36

1 Experimental procedures

1.1 General methods

Commercially available reagents and solvents were used without purification except where noted. ^{1}H and ^{13}C NMR spectra were recorded on a Bruker Ascend 400 spectrometer, a Bruker biospin AVANCE NEO 700 spectrometer (cryogenic probe accessory), and a JEOL ECA500 spectrometer, and chemical shifts were reported on the delta scale in ppm relative to residual chloroform (δ = 7.26 and 77.16 for ^{1}H and ^{13}C, respectively) and pyridine (δ = 8.74 and 150.35 for ^{1}H and ^{13}C, respectively). Structural assignments were made with additional information from DQF COSY, gCOSY, NOESY, and HSQC experiments. UV-Vis absorption spectra were recorded on a JASCO V-760 spectrometers. High-resolution mass spectra (HRMS) were recorded on a Thermo Fisher Scientific LTQ Orbitrap XL hybrid FTMS by electron spray ionization (ESI) methods. Melting points (Mp) were measured with an AS ONE micro melting point apparatus. Preparative separations were performed by silica gel gravity column chromatography (silica gel 60 N (spherical, neutral)). Preparative medium pressure liquid chromatography (MPLC) separations were carried out on a YAMAZEN smart flash EPCLC AL-580S using a preparative Bio-Beads (S-X1) column. **2**,[S1] **3**,[S2,S3], **6**[S4], **G4**[S5], and **G5**[S6] were synthesized according to reported methods.

1.2 Synthesis of **1Zn**

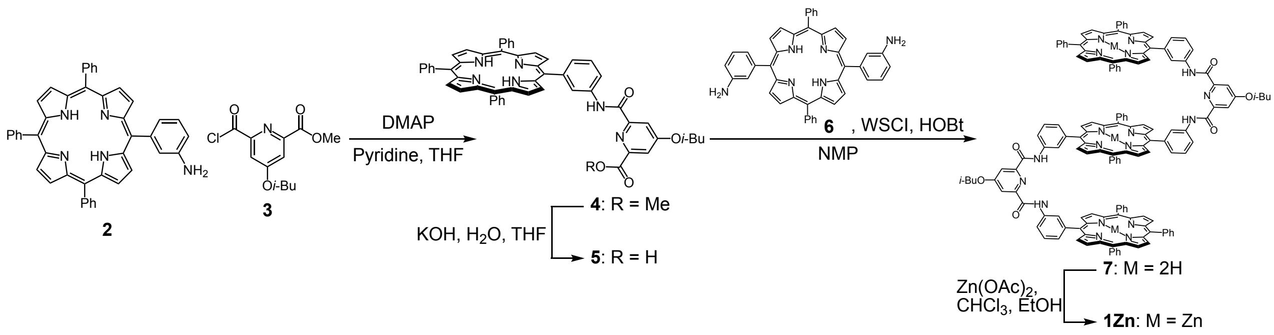

Scheme S1. Synthesis of **1Zn**. DMAP: 4-dimethylaminopyridine, WSCI: 1-ethyl-3-(3-dimethylaminopropyl)carbodiimide hydrochloride, HOBt: 1-hydroxybenzotriazole.

Methyl 4-isobutoxy-6-((3-(10,15,20-triphenylporphyrin-5-yl)phenyl)carbamoyl)picolinate (4).

To a solution of aminoporphyrin **2** (496 mg, 0.79 mmol) in THF (23 mL) were added a solution of 4-isobutoxy-6-(methoxycarbonyl)picolinic acid chloride **3** (321 mg, 1.18 mmol), DMAP (965 mg, 7.9 mmol) and pyridine (0.10 mL, 1.24 mmol) in THF (14 mL) at 0 °C. After the mixture was stirred at room temperature for 1 h, the reaction mixture was poured into aqueous sodium bicarbonate and the aqueous layer was extracted with chloroform. The organic layer was washed with brine, dried over sodium sulfate, and concentrated under reduced pressure. The residue was purified by silica-gel column chromatography (50% dichloromethane–hexane) and subsequent MPLC (chloroform) to give a purple solid (458 mg, 67%). Mp: > 300 °C. ^{1}H NMR (chloroform-*d*, 400 MHz): δ 10.32 (s, 1H), 8.94 (d, 2H, J = 4.8 Hz), 8.88 (d, 2H, J = 4.8 Hz), 8.87 (s, 4H), 8.54 (s, 1H), 8.43 (d, 1H, J = 8.0 Hz), 8.19–8.30 (m, 6H), 8.04 (d, 1H, J = 8.0 Hz), 7.92 (s, 1H), 7.73–7.82 (m, 10H), 7.68 (s, 1H), 3.89 (s, 3H), 3.87 (d, 2H, J = 6.2 Hz), 2.13 (m, 1H), 1.04 (d, 6H, J = 6.6 Hz) –2.76 (s, 2H). ^{13}C{^{1}H} NMR (chloroform-*d*, 100 MHz): δ 167.9, 165.1, 161.9, 152.0, 148.1, 143.2, 142.3, 142.3, 136.2, 134.7, 134.7, 131.3, 131.2, 127.9, 127.6, 126.8, 126.4, 120.4, 120.4, 119.6, 119.5, 115.0, 111.0, 75.4, 53.0, 28.1, 19.2. HRMS (ESI$^+$) m/z: [M+H]$^+$ Calcd for $C_{56}H_{45}O_4N_6$ 865.3497. Found 865.3504.

4-Isobutoxy-6-((3-(10,15,20-triphenylporphyrin-5-yl)phenyl)carbamoyl)picolinic acid (5).

To a solution of **4** (315 mg, 0.36 mmol) in THF (36 mL) was added 0.5 M aqueous potassium hydroxide (9.0 mL) at 0 °C. After the mixture was stirred at room temperature for 1 h, the resulting mixture was poured into water, and the pH value of the aqueous layer was adjusted to 3. The aqueous layer was extracted with chloroform. The organic layer was washed with water, dried over sodium sulfate, and concentrated under reduced pressure. The resulting purple solid (308 mg, 99%) was used for the next step without further purification. Mp: > 300 °C. ^{1}H NMR (pyridine-*d*$_5$, 400 MHz): δ 11.28 (s, 1H), 9.19 (d, 2H, J = 4.8 Hz), 9.07 (d, 2H, J = 4.8 Hz), 9.06 (s, 4H), 9.01 (s, 1H), 8.66 (d, 1H, J = 7.5 Hz), 8.34–8.44 (m, 6H), 8.22 (d, 1H, J = 2.3 Hz), 8.18 (d, 1H, J = 7.5 Hz), 8.11 (d, 1H, J = 2.3 Hz), 7.76–7.84 (m, 10H), 3.76 (d, 2H, J = 6.5 Hz), 2.00 (m, 1H), 0.93 (d, 6H, J = 6.7 Hz), –2.36 (s, 2H). ^{13}C{^{1}H} NMR (pyridine-*d*$_5$, 100 MHz): δ 168.5, 167.8, 163.3, 153.0, 151.6, 143.6, 143.0, 142.9, 138.0, 135.5, 132.3, 131.6, 128.8, 128.2, 127.8, 127.4, 121.3, 121.3, 120.8, 120.6, 115.2, 111.9, 75.5, 28.7, 19.4. HRMS (ESI$^+$) m/z: [M+H]$^+$ Calcd for $C_{55}H_{43}O_4N_6$ 851.3340. Found 851.3345.

Free-base trisporphyrin (7).

To a solution of **5** (158 mg, 0.19 mmol) and **6** (60 mg, 93 μmol) in NMP (0.9 mL) were added HOBt (38 mg, 0.28 mmol) and WSCI•HCl (53 mg, 0.28 mmol). After the mixture was stirred at room temperature for 2 h, the reaction solution was diluted by adding chloroform. The organic solvent was washed with brine, dried over sodium sulfate, and concentrated under reduced pressure. The residue was purified by silica-gel column chromatography (10% ethyl acetate–dichloromethane) and subsequent MPLC (chloroform) to give a purple solid (189 mg, 88%). Mp: > 300 °C. ^{1}H NMR (pyridine-d_5, 500 MHz): δ 11.02 (s, 4H), 9.06 (d, 4H, J = 4.9 Hz), 9.02 (d, 4H, J = 4.9 Hz), 9.01 (d, 4H, J = 4.9 Hz), 9.00 (d, 4H, J = 4.9 Hz), 8.90 (d, 4H, J = 4.9 Hz), 8.88 (d, 4H, J = 4.9 Hz), 8.80 (br, 4H), 8.33–8.42 (m, 8H), 8.29 (s, 4H), 8.18–8.26 (m, 8H), 8.01–8.16 (m, 8H), 7.81–7.87 (m, 6H), 7.76 (t, 4H, J = 7.9 Hz), 7.63–7.72 (m, 14H), 7.49–7.57 (m, 4H), 3.95 (*overlapped*, 4H), 2.09 (m, 2H), 1.02 (d, 12H, J = 6.8 Hz), –2.30 (s, 4H), –2.38 (s, 2H). ^{13}C{^{1}H} NMR (pyridine-d_5, 100 MHz): δ 168.8, 163.6, 152.8, 143.3, 143.2, 142.9, 142.9, 142.8, 142.7, 142.7, 142.6, 137.9, 135.4, 135.3, 135.1, 131.7, 128.8, 128.6, 128.6, 128.5, 128.5, 128.4, 128.3, 127.9, 127.9, 127.8, 127.6, 127.5, 121.8, 121.3, 121.3, 121.2, 121.1, 120.9, 120.8, 120.7, 120.6, 120.6, 112.8, 75.5, 28.6, 19.4. HRMS (ESI^{+}) m/z: $[M+2H]^{2+}$ Calcd for $C_{154}H_{114}O_6N_{18}$ 1155.4579. Found 1155.4588.

Zinc trisporphyrin (1Zn).

To a solution of free-base trisporphyrin **7** (100 mg, 43 μmol) in chloroform (24 mL) was added a solution of zinc acetate (110 mg, 0.60 mmol) in ethanol (20 mL). After stirring at room temperature for 20 min, the organic layer was washed with water, dried over sodium sulfate, and concentrated under reduced pressure to give a purple solid (101 mg, 94%). Mp: > 300 °C. ^{1}H NMR (pyridine-d_5, 400 MHz): δ 10.99 (s, 4H), 9.10–9.17 (m, 16H), 9.06 (d, 4H, J = 4.9 Hz), 9.05 (d, 4H, J = 4.9 Hz), 8.90 (br, 4H), 8.41 (m, 4H), 8.35 (d, 8H, J = 7.2 Hz), 8.29 (d, 4H, J = 7.5 Hz), 8.26 (*overlapped*, 4H), 8.26 (s, 4H), 8.12 (d, 4H, J = 7.5 Hz), 7.79–7.84 (m, 6H) , 7.70–7.79 (m, 14H), 7.67 (*overlapped*, 4H), 7.66 (t, 4H, J = 7.5 Hz), 3.95 (d, 4H, J = 6.5 Hz), 2.10 (m, 2H), 1.01 (d, 12H, J = 6.5 Hz). ^{13}C{^{1}H} NMR (pyridine-d_5, 100 MHz): δ 168.8, 163.5, 152.9, 151.1, 151.1, 151.0, 151.0, 151.0, 150.9, 144.8, 144.4, 144.3, 144.3, 137.5, 135.5, 135.4, 135.3, 132.8, 131.9, 128.7, 128.3, 128.2, 128.1, 127.5, 127.4, 127.3, 127.3, 121.9, 121.8, 121.4, 121.2, 112.7, 75.6, 28.6, 19.4. HRMS (ESI^{+}) m/z: M^{3+} Calcd for $C_{154}H_{106}O_6N_{18}Zn_3$ 831.5467. Found 831.5474.

1.3 Synthesis of **9Zn**

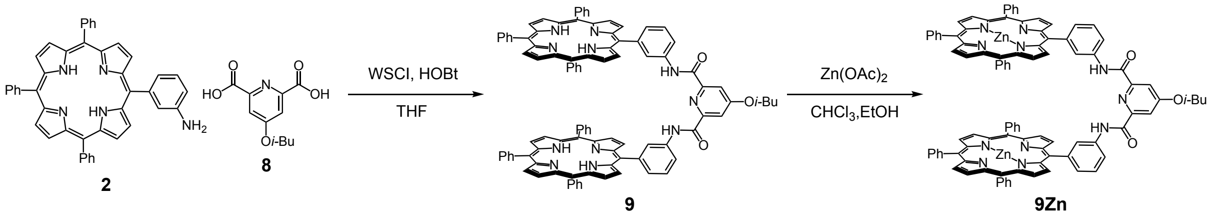

Scheme S2. Synthesis of **9Zn**. WSCI: 1-ethyl-3-(3-dimethylaminopropyl)carbodiimide hydrochloride, HOBt: 1-hydroxybenzotriazole.

Free-base bisporphyrin (9).

To a solution of **2** (600 mg, 0.40 mmol) and **8** (48 mg, 0.20 μmol) in THF (4.0 mL) were added HOBt (59 mg, 0.44 mmol) and WSCI•HCl (167 mg, 0.87 mmol). After the mixture was stirred at room temperature for 2 h, the reaction solution was diluted by adding chloroform. The organic layer was washed with brine, dried over sodium sulfate, and concentrated under reduced pressure. The residue was purified by silica-gel column chromatography (10% ethyl acetate–dichloromethane) and subsequent MPLC (chloroform) to give a purple solid (244 mg, 84%). Mp: > 300 °C. ^{1}H NMR (pyridine-d_5, 400 MHz): δ 11.42 (s, 2H), 9.09 (d, 4H, J = 4.9 Hz), 9.03 (d, 4H, J = 4.9 Hz), 8.96 (d, 4H, J = 4.9 Hz), 8.85 (d, 4H, J = 4.9 Hz), 8.79 (br, 2H), 8.49 (d, 2H, J = 8.2 Hz), 8.44 (d, 2H, J = 6.5 Hz), 8.38 (d, 2H, J = 6.5 Hz), 8.29 (d, 4H, J = 6.9 Hz), 8.23 (s, 2H), 8.07 (d, 4H, J = 6.9 Hz), 8.03 (d, 2H, J = 8.2 Hz), 7.79–7.89 (m, 6H), 7.67–7.77 (m, 8H), 7.63 (t, 2H, J = 8.2 Hz), 7.48 (t, 4H, J = 6.9 Hz), 3.70 (d, 2H, J = 6.4 Hz), 1.93 (m, 1H), 0.88 (d, 6H, J = 6.4 Hz), –2.50 (s, 4H). ^{13}C{^{1}H} NMR (pyridine-d_5, 100 MHz): δ 168.7, 163.5, 152.7, 143.2, 143.0, 142.8, 137.8, 135.5, 135.4, 135.3, 135.2, 132.1, 131.6, 128.8, 128.6, 128.4, 127.9, 127.8, 127.7, 127.6, 124.5, 123.4, 121.8, 121.3, 121.1, 120.6, 112.7, 75.5, 28.6, 19.4. HRMS (ESI$^+$) m/z: $[M+2H]^{2+}$ Calcd for $C_{99}H_{73}O_3N_{11}$ 731.7943. Found 731.7950.

Zinc bisporphyrin (9Zn).

To a solution of free-base bisporphyrin **9** (60 mg, 41 μmol) in chloroform (22 mL) was added a solution of zinc acetate (69 mg, 0.38 mmol) in ethanol (12 mL). After stirring at room temperature for 20 min, the organic layer was washed with water, dried over sodium sulfate, and concentrated under reduced pressure to give a purple solid (72 mg, 99%). Mp: > 300 °C. ^{1}H NMR (pyridine-d_5, 400 MHz): δ 11.39 (s, 2H), 9.19 (d, 4H, J = 4.6 Hz), 9.16 (d, 4H, J = 4.6 Hz), 9.14 (d, 4H, J = 4.6 Hz), 9.05 (d, 4H, J = 4.6 Hz), 8.90 (br, 2H), 8.39–8.46 (m, 4H), 8.36 (*overlapped*, 4H), 8.36 (*overlapped*, 2H), 8.29 (d, 4H, J = 7.9 Hz), 8.27 (s, 2H), 8.10 (d, 2H, J = 7.5 Hz), 7.77–7.84 (m, 6H) , 7.70–7.76 (m, 8H), 7.63 (*overlapped*, 4H), 7.62 (t, 2H, J = 7.5 Hz), 3.78 (d, 2H, J = 6.5 Hz), 1.99 (m, 1H), 0.91 (d, 6H, J = 6.5 Hz). ^{13}C{^{1}H} NMR (pyridine-d_5, 100 MHz): δ 168.8, 163.6, 152.9, 151.1, 151.1, 151.0, 144.8, 144.4, 144.3, 137.7, 135.6, 135.5, 132.8, 132.8, 131.9, 128.8, 128.3, 128.2, 127.5, 127.5, 127.4, 124.5, 121.9, 121.8, 121.4, 121.3, 112.7, 75.6, 28.7, 19.5. HRMS (ESI$^+$) m/z: M^{2+} Calcd for $C_{99}H_{67}O_3N_{11}Zn_2$ 792.7000. Found 792.7009.

1.4 NMR and ESI spectra

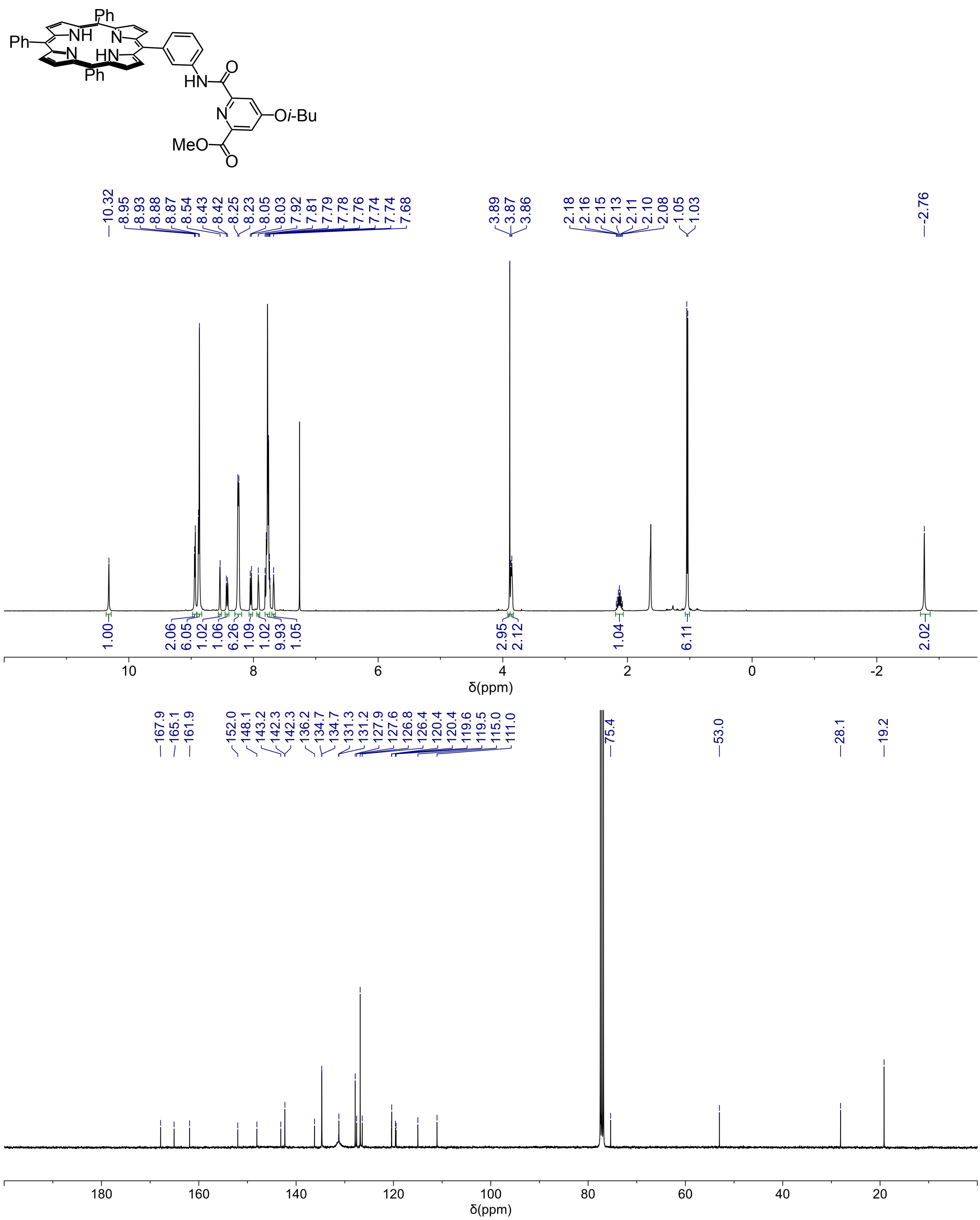

Figure S1. ^{1}H (400 MHz, 298 K) and $^{13}C\{^{1}H\}$ (100 MHz, 298 K) NMR spectra of **4** in chloroform-*d*.

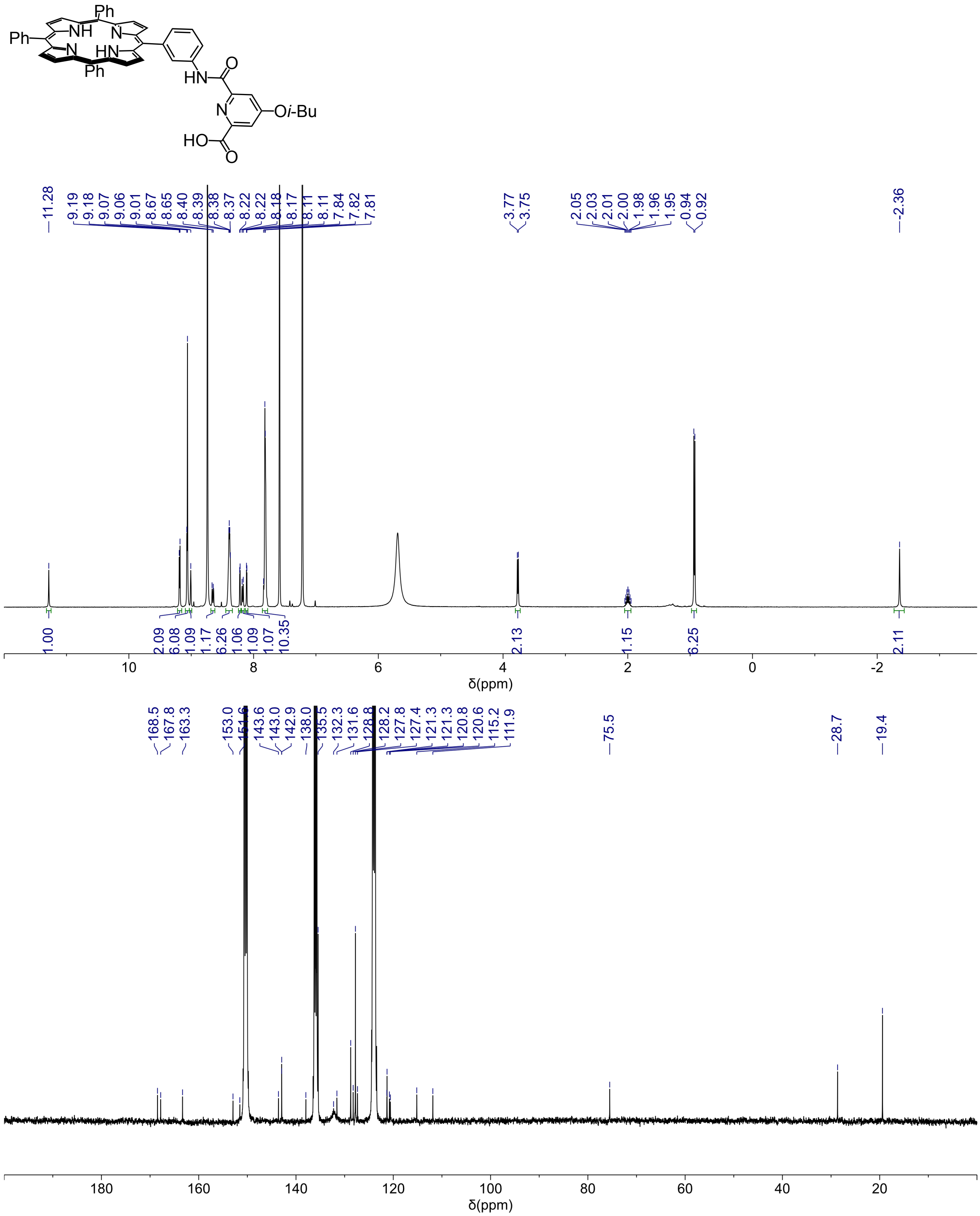

Figure S2. ^{1}H (400 MHz, 298 K) and ^{13}C{^{1}H } (100 MHz, 298 K) NMR spectra of **5** in pyridine-d_5.

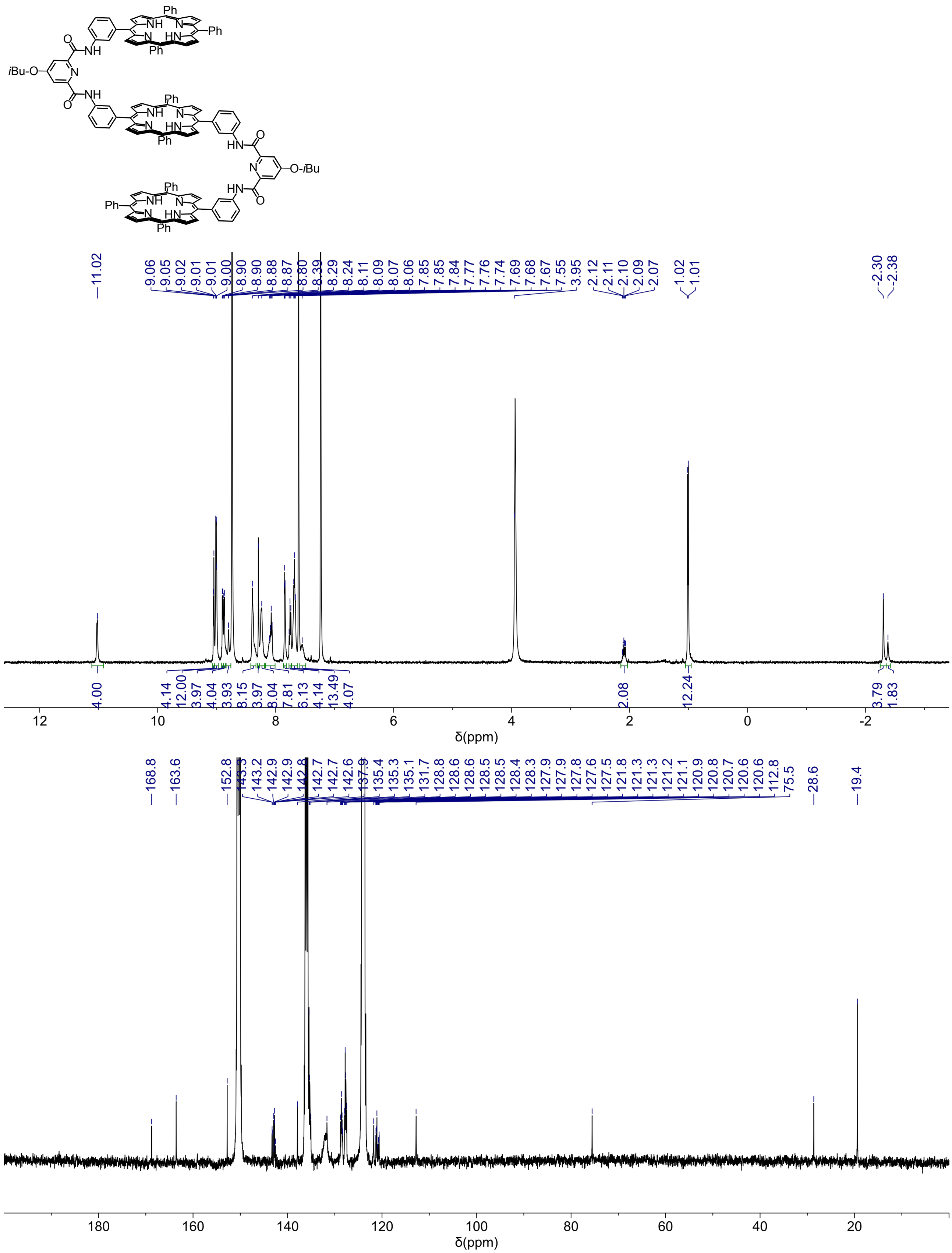

Figure S3. ^{1}H (500 MHz, 398 K) and $^{13}C\{^{1}H\}$ (100 MHz, 298 K) NMR spectra of **7** in pyridine-d_5.

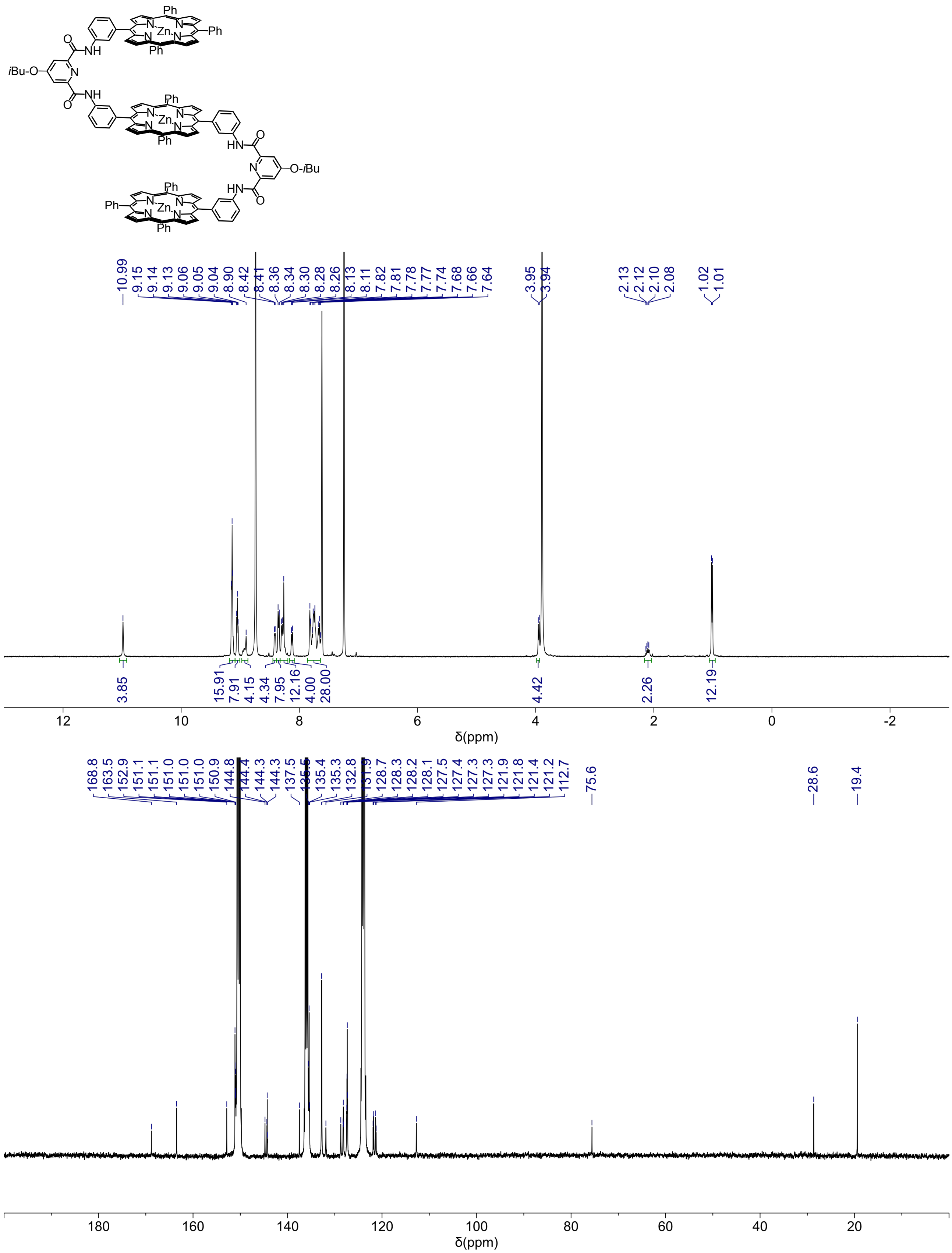

Figure S4. ^{1}H (400 MHz, 398 K) and ^{13}C{^{1}H } (100 MHz, 298 K) NMR spectra of **1Zn** in pyridine-d_5.

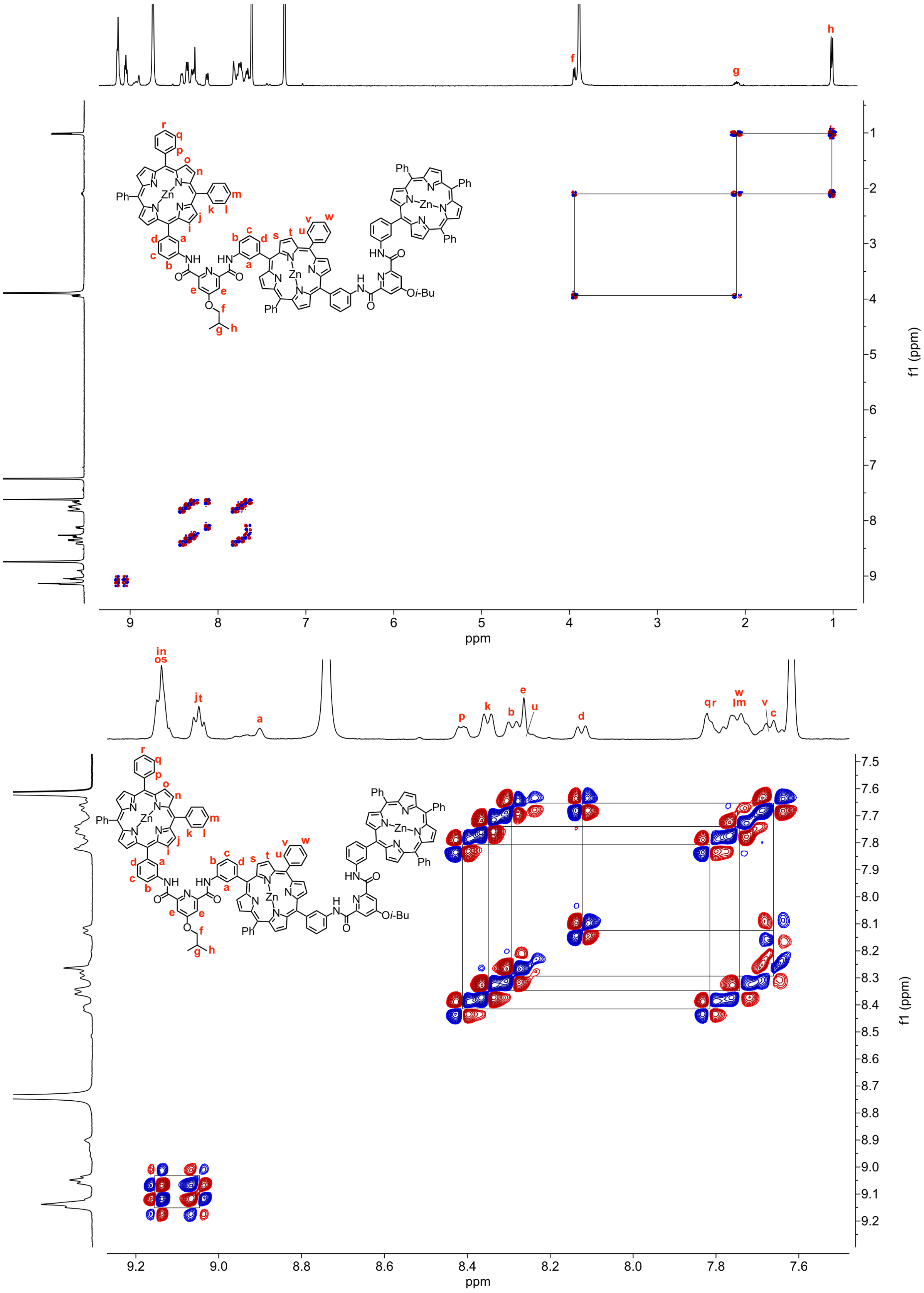

Figure S5. DQF COSY (400 MHz, 398 K) of **1Zn** in pyridine-d_5.

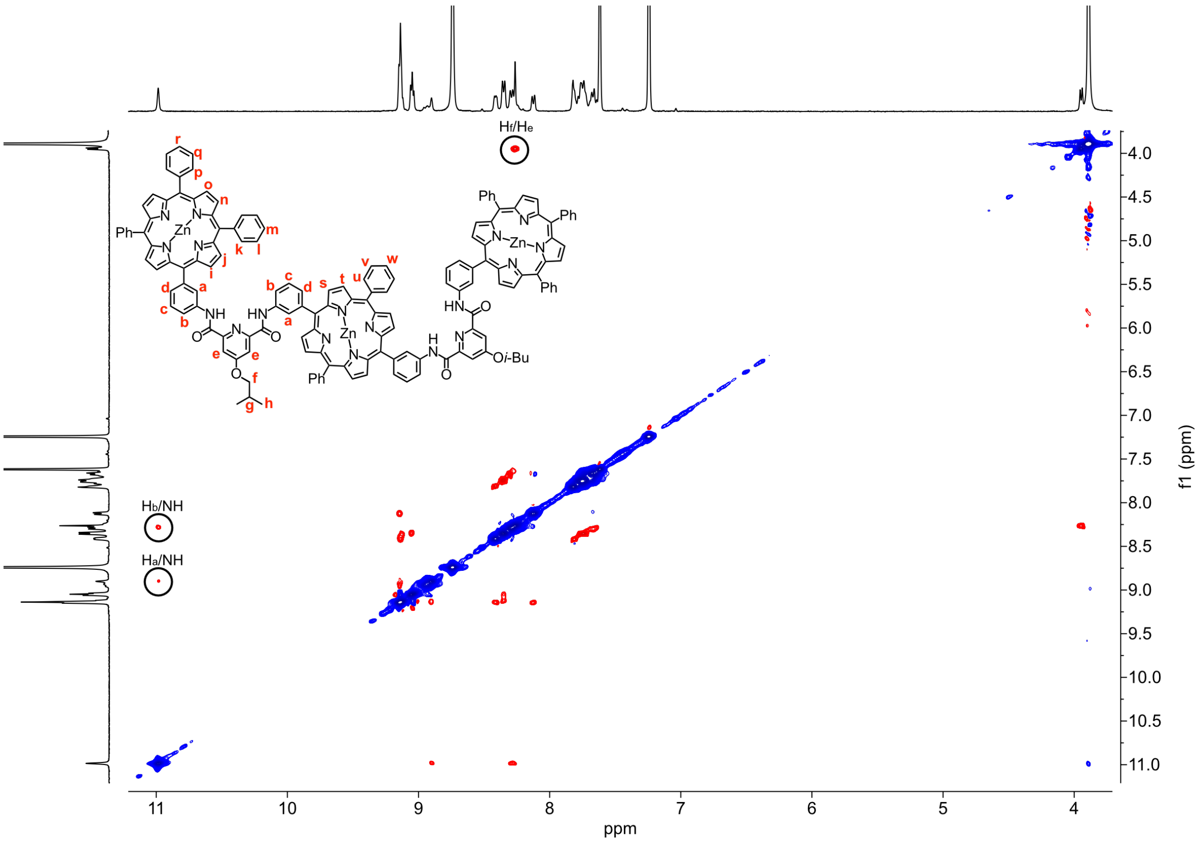

Figure S6. NOESY (400 MHz, 398 K) of **1Zn** in pyridine-d_5.

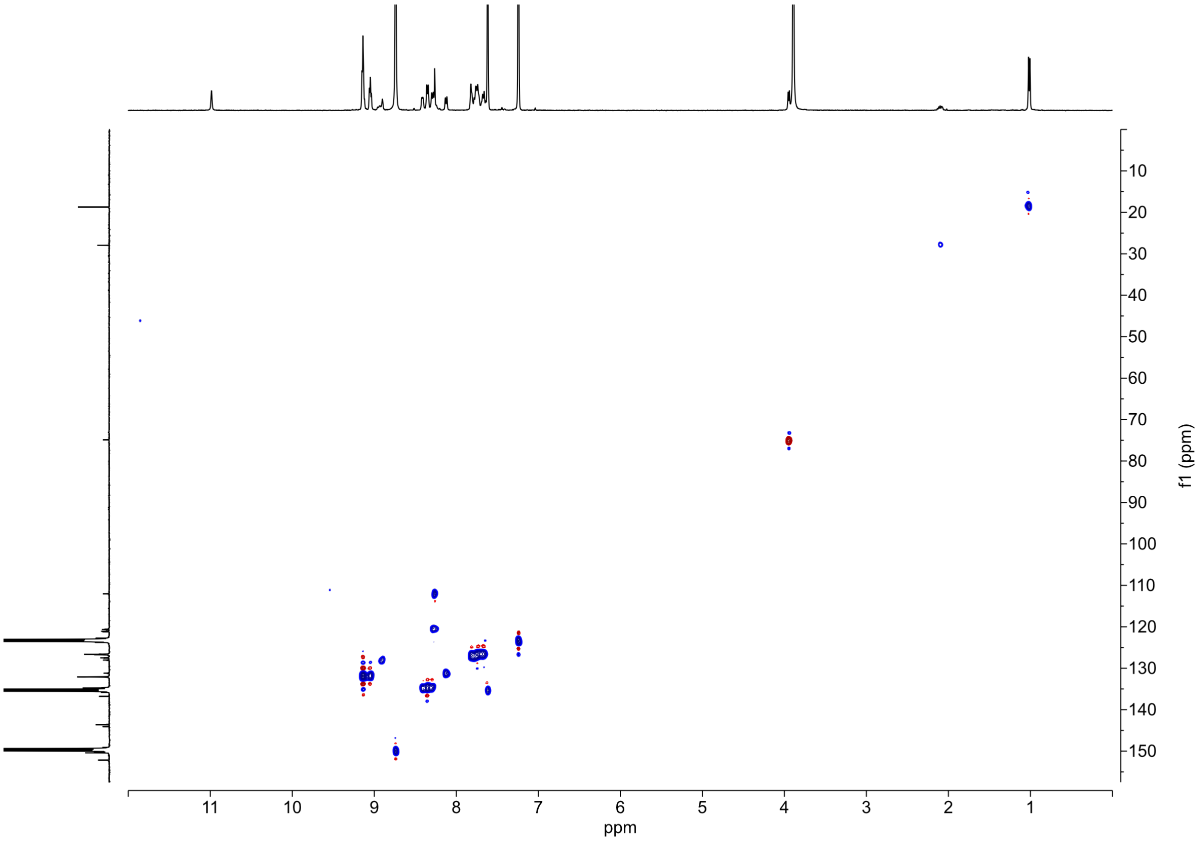

Figure S7. HSQC (400 MHz, 398 K) of **1Zn** in pyridine-d_5.

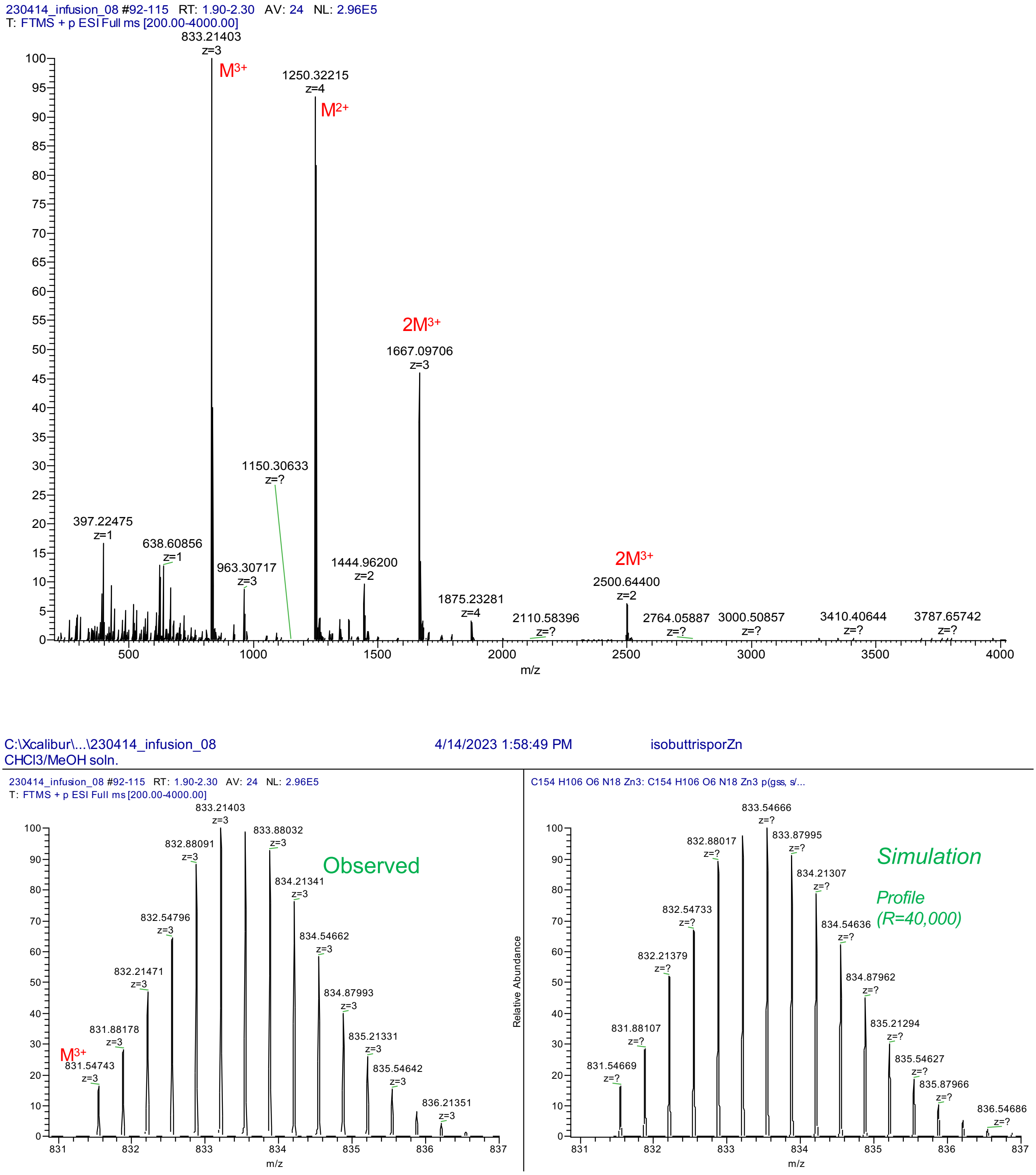

Figure S8. ESI-MS spectra of **1Zn**.

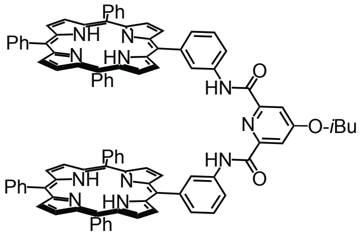

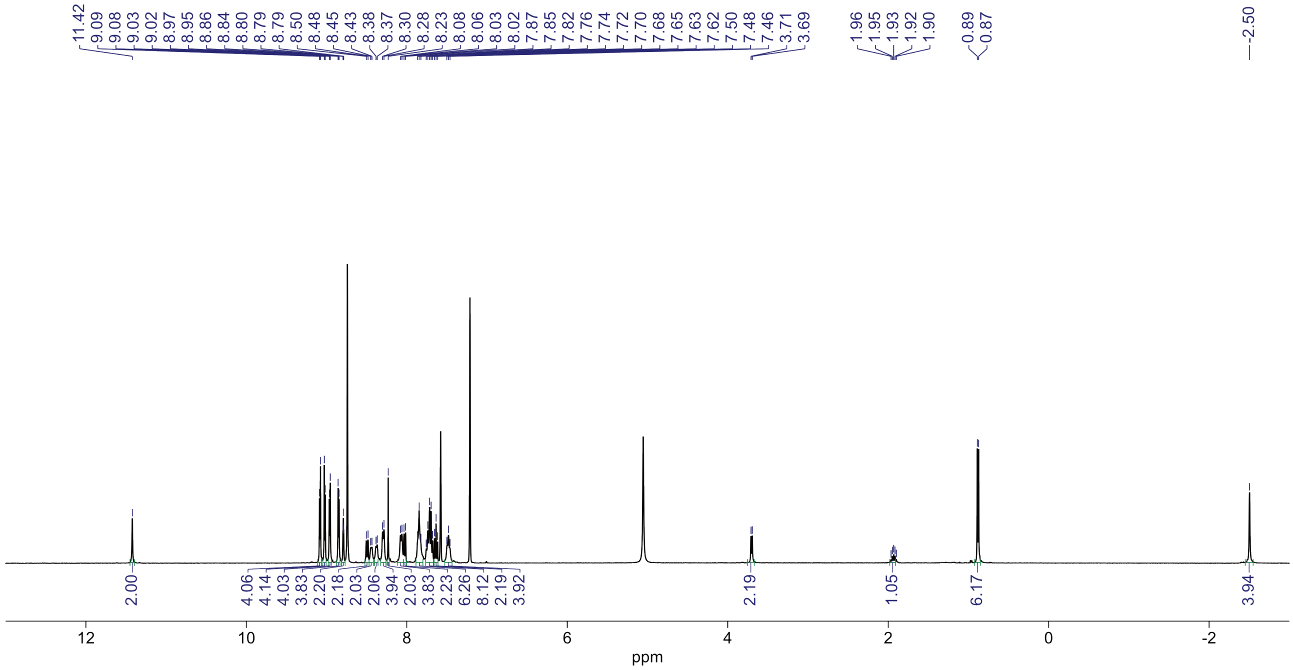

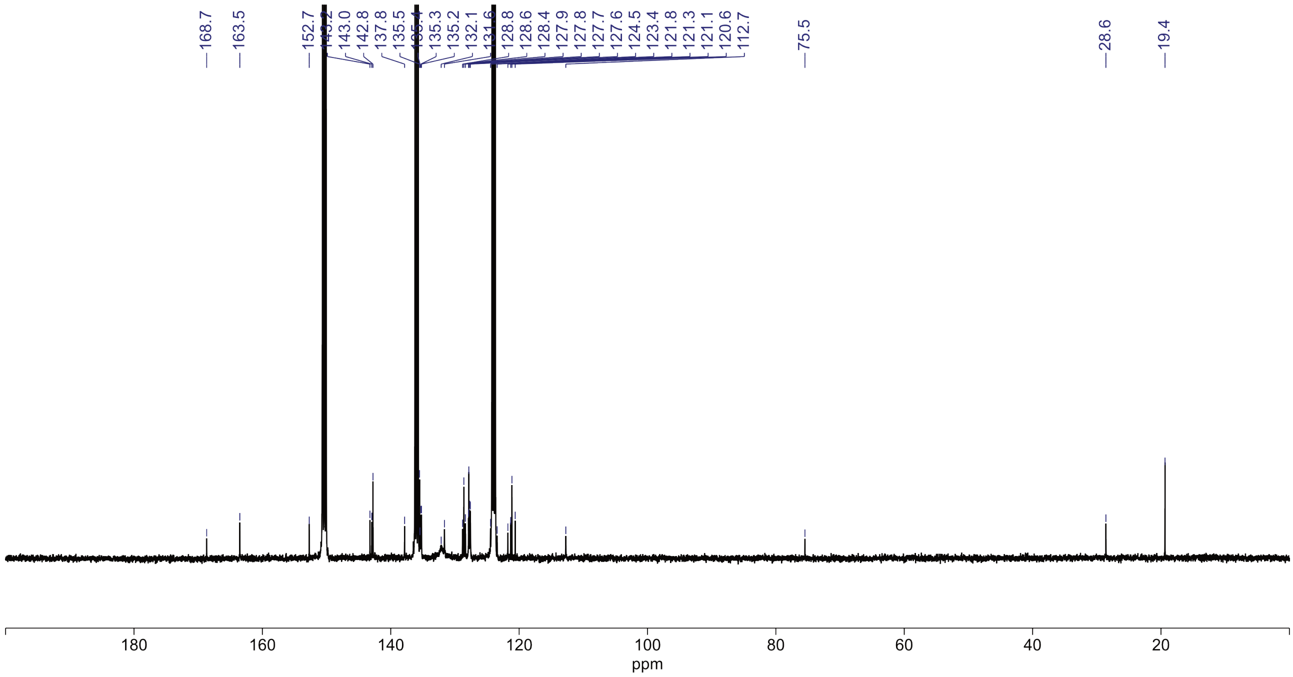

Figure S9. ^{1}H (400 MHz, 298 K) and $^{13}C\{^{1}H\}$ (100 MHz, 298 K) NMR spectra of **9** in pyridine-d_5.

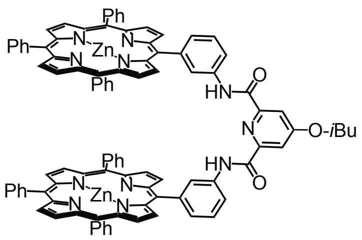

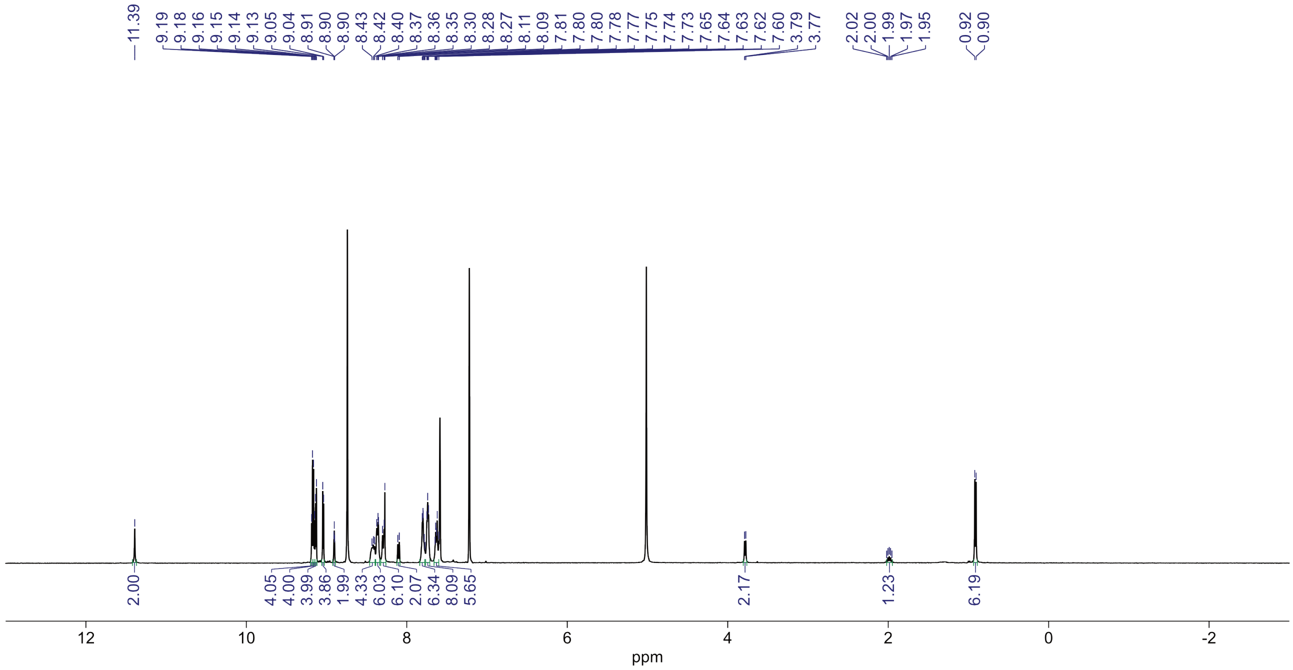

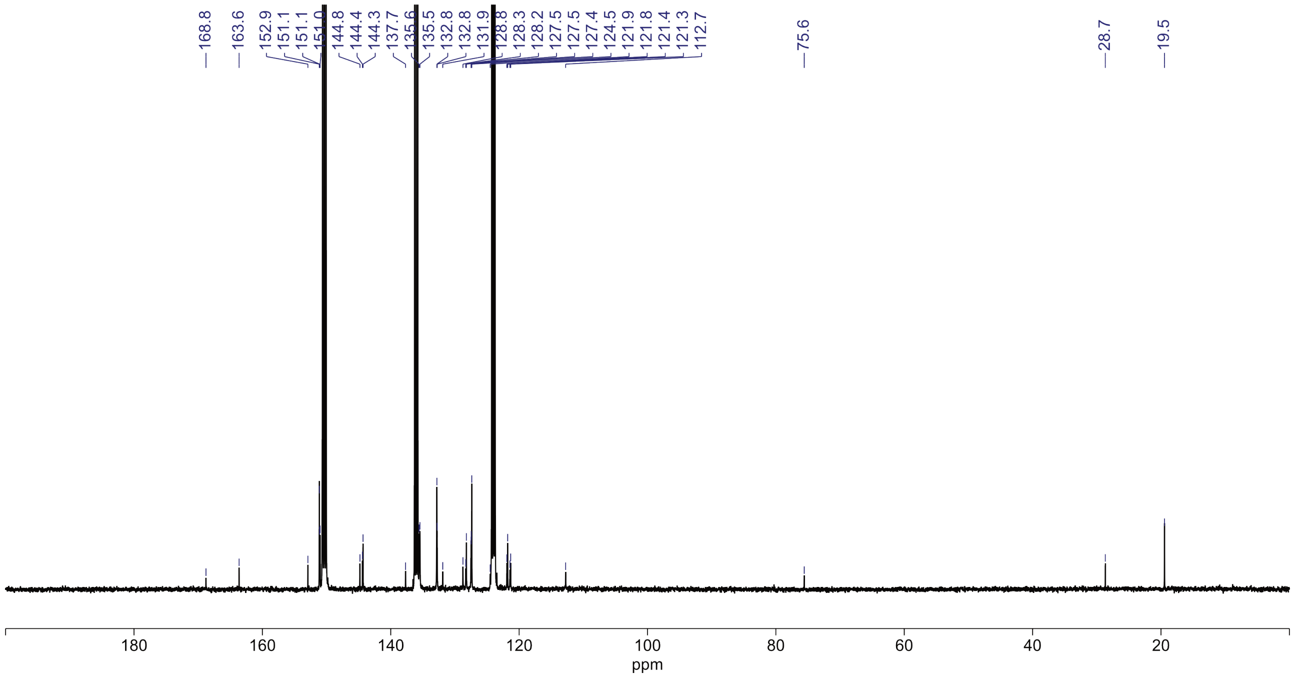

Figure S10. ^{1}H (400 MHz, 298 K) and ^{13}C{^{1}H } (100 MHz, 298 K) NMR spectra of **9Zn** in pyridine-d_5.

2 Titration experiments and binding constants

2.1 UV-Vis studies of binding behavior of **1Zn**

A standard titration technique was employed to determine the binding constants for the host–guest complexes of **1Zn** and the guest molecules. A titration was performed wherein the concentration of a host solution was fixed while varying the concentration of guests. During the course of the titration, UV-Vis absorption changes were measured from 350 nm to 900 nm. The plots of the absorption changes versus guest concentrations were fitted by non-linear analysis using a program of TitrationFit software[S7] to determine the binding constants.

The non-linear curve-fitting for a 1:1 complex was based on the following equations:[S8]

$$[HG] = \frac{1}{2}\left([G_0] + [H_0] + \frac{1}{K_a}\right) - \sqrt{\left(G_0 + H_0 + \frac{1}{K_a}\right)^2 - 4[H_0][G_0]}$$

$$\Delta A_{obs} = \varepsilon_{\Delta HG}([HG])$$

Where $[HG]$ represents the concentration of the host-guest complex, $[G_0]$ and $[H_0]$ are the initial concentrations of the guest and host, K_a is the binding constant for the1:1 complexation.

The non-linear curve-fitting for a 1:2 complex was based on the following equations:[S8]

$$\Delta A_{obs} = \frac{\varepsilon_{\Delta HG}[H_0]K_1[G] + \varepsilon_{\Delta HG_2}[H_0]K_1K_2[G]^2}{1 + K_1[G] + K_1K_2[G]^2}$$

Where K_1 and K_2 represent the first and second binding constants, the ε are the extinction coefficient differences, $[H_0]$ is the initial concentration of the host, and $[G]$ is the concentration of free guest. [G] is not directly observable but can be determined indirectly as the roots of the cubic equation:

$$A[G]^3 + B[G]^2 + C[G] - [G_0] = 0$$

Where $A = K_1K_2$, $B = K_1(2K_2[H_0] - K_2[G_0] + 1)$, and $C = K_1([H_0] - [G_0]) + 1$.

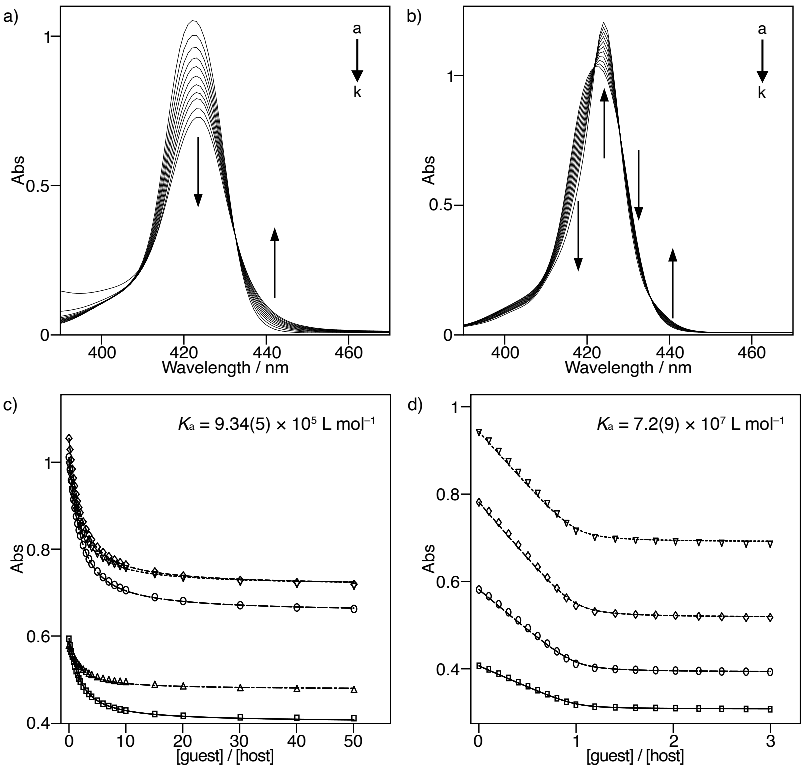

Figure S11. UV-Vis absorption spectra of **1Zn** (8.1×10^{-7} mol L^{-1}) in the presence of (a) **G1** (a-k: 0.0, 0.31, 0.65, 1.0, 1.4, 2.0, 2.8, 4.0, 5.7, 12, and 41 × 10^{-6} mol L^{-1}) and (b) **G2** (a-k: 0.0, 0.16, 0.31, 0.48, 0.65, 0.82, 1.0, 1.2, 1.4, 1.6, and 12 × 10^{-6} mol L^{-1}) in chloroform at 295 K. Plots of Abs of (c) **G1** and (d) **G2** against [guest] / [host] and the fitting curves obtained by 1:1 fitting model.

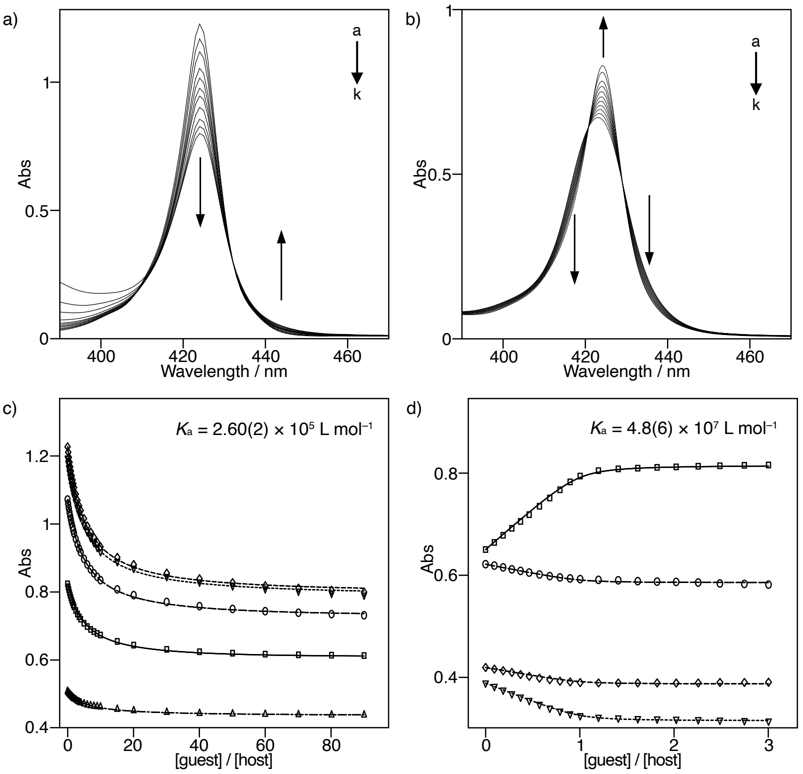

Figure S12. UV-Vis absorption spectra of **1Zn** (8.1×10^{-7} mol L^{-1}) with (a) **G2** (8.1×10^{-7} mol L^{-1}) in the presence of **G1** (a-k: 0.0, 0.65, 1.4, 2.8, 4.0, 5.6, 7.2, 12, 24, 41, and 97 × 10^{-6} mol L^{-1}) and (b) **1Zn** (8.0×10^{-7} mol L^{-1}) with **G1** (1.6×10^{-5} mol L^{-1}) in the presence of **G2** (a-k: 0.0, 0.75, 1.5, 2.2, 3.0, 3.7, 4.6, 5.5, 6.3, 8.0, and 24 × 10^{-7} mol L^{-1}) in chloroform at 295 K. Plots of Abs of (c) **G1** and (d) **G2** against [guest] / [host] and the fitting curves obtained by 1:1 fitting model.

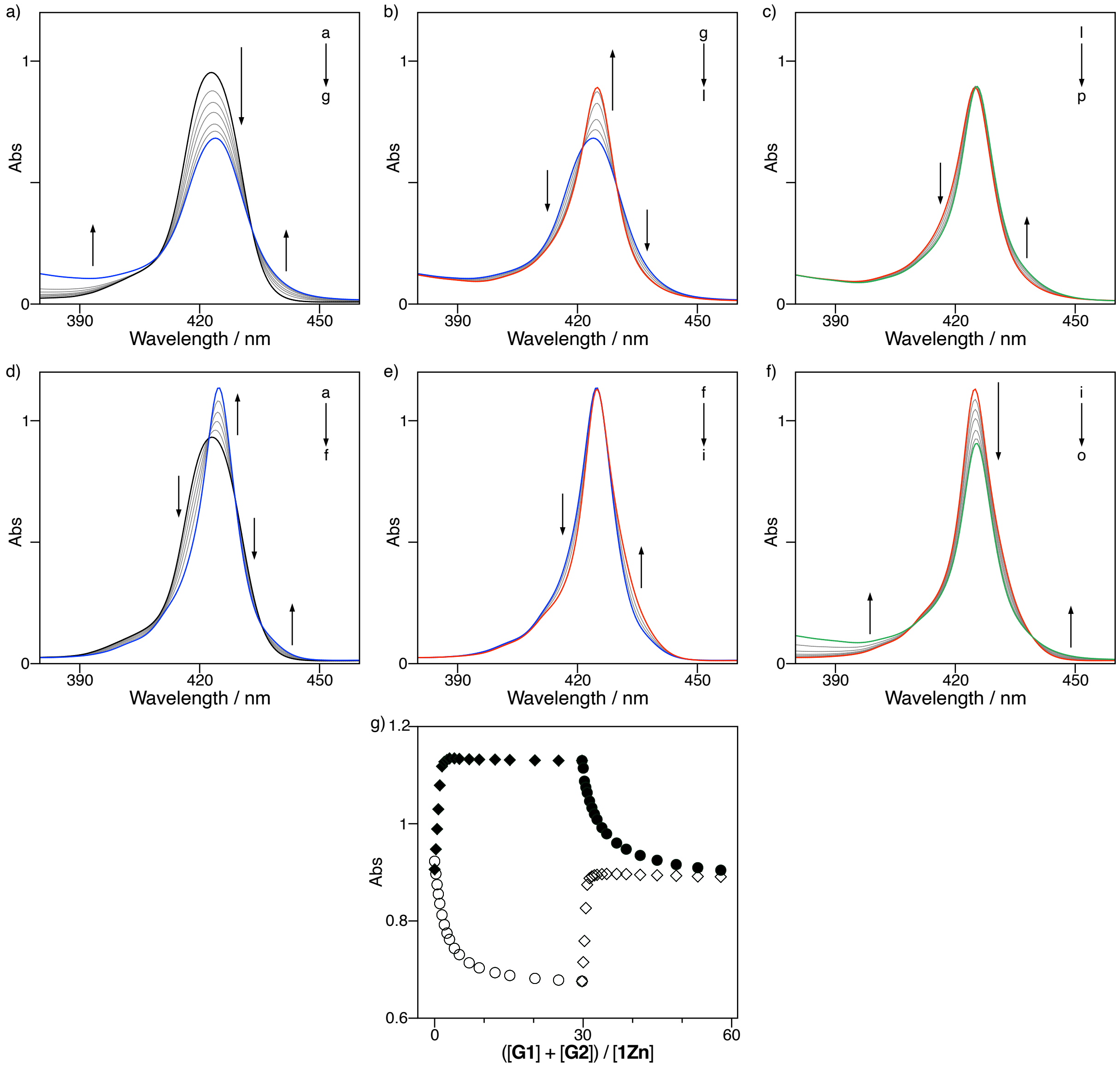

Figure S13. (a–c) UV-Vis absorption spectral changes of **1Zn** (8.0×10^{-7} mol L^{-1}) upon addition of **G1** (a–g: 0.0, 0.60, 1.2, 2.0, 4.0, 7.2, and 24 × 10^{-6} mol L^{-1}) and then **G2** (g–p: 0.0, 0.20, 0.39, 0.61, 0.83, 1.6, 4.0, 7.1, 12 and 22 × 10^{-6} mol L^{-1}) at 295 K in chloroform. (d–f) UV-Vis absorption spectral changes of **1Zn** (8.0×10^{-7} mol L^{-1}) upon addition of **G2** (a–i: 0.0, 0.20, 0.40, 0.60, 0.83, 3.2, 5.6, 12, and 24 × 10^{-6} mol L^{-1}) and then **G1** (i–o: 0.0, 0.39, 1.2, 2.4, 5.6, 12, and 22 × 10^{-5} mol L^{-1}) at 295 K in chloroform. (g) Plots of Abs at 425 nm of **1Zn** upon adding **G1** (open circle) and then **G2** (open rhombus) and upon adding **G2** (filled rhombus) and then **G1** (filled circle) against ([**G1**] + [**G2**]) / [**1Zn**].

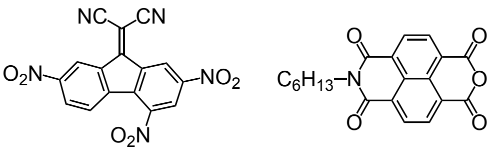

G4 **G5**

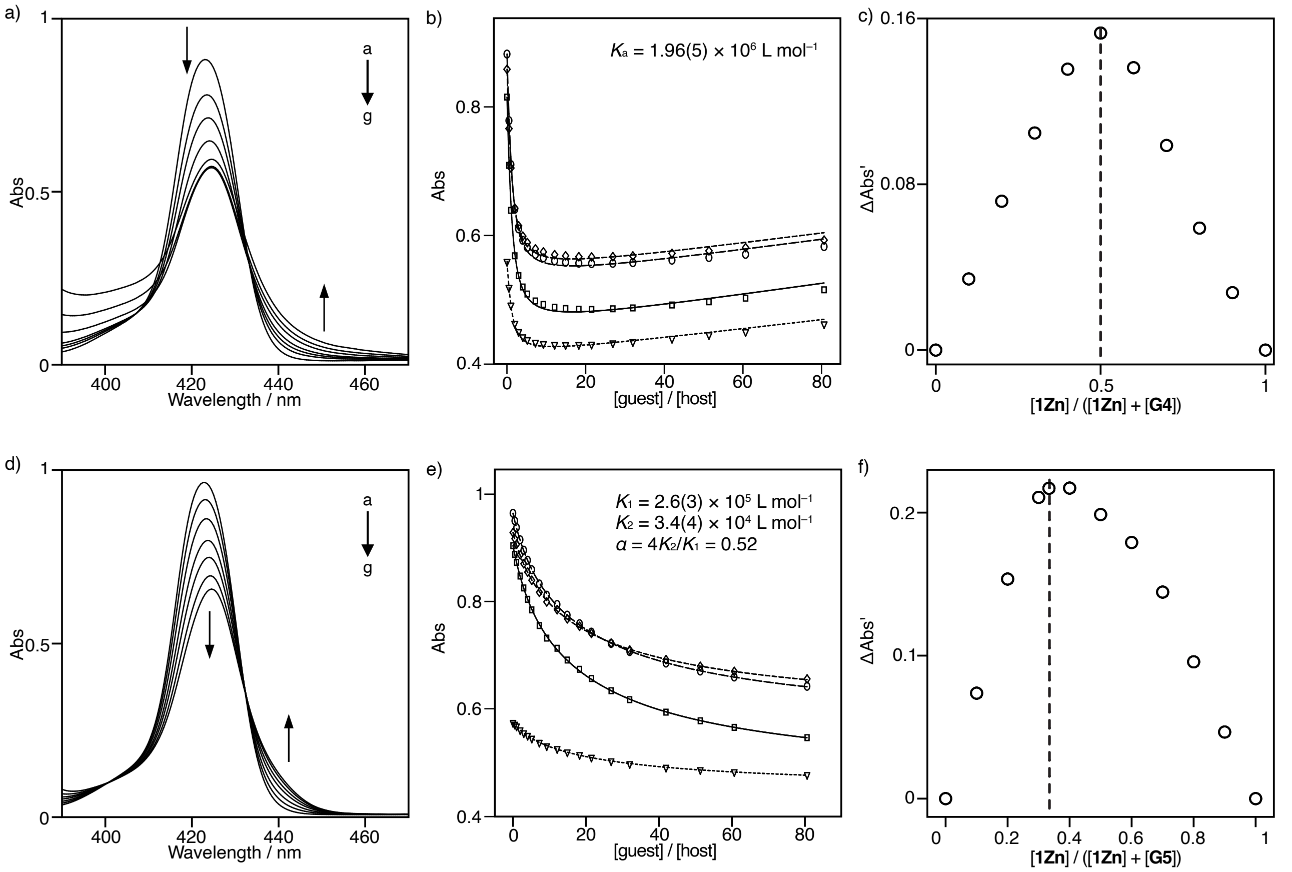

Figure S14. UV-Vis absorption spectra of **1Zn** (8.0×10^{-7} mol L^{-1}) in the presence of (a) **G4** (a-g: 0.0, 0.40, 0.80, 1.6, 12, 34 and 65 × 10^{-6} mol L^{-1}) and (b) **G5** (a-g: 0.0, 1.6, 4.1, 9.7, 17, 34, and 65 × 10^{-6} mol L^{-1}) in chloroform at 295 K. Plots of Abs of (b) **G4** and (e) **G5** against [guest] / [host] and the fitting curves obtained by 1:1 fitting model, 1:2 fitting model, respectively. Job plots **1Zn** with (c) **G4** and (f) **G5** at 295K in chloroform. The total concentration of a mixture of host and guest was maintained at a constant value (**1Zn** + guests: 2.0×10^{-4} mol L^{-1}). ΔAbs' indicates $|\mathrm{Abs} - \mathrm{Abs_H}{\cdot}X - \mathrm{Abs_G}{\cdot}X|$, where Abs, $\mathrm{Abs_H}$, and $\mathrm{Abs_G}$ indicate the observed absorbance, absorbance of the host, and absorbance of the guest, respectively.

2.2 UV-Vis studies of binding behavior of **9Zn**

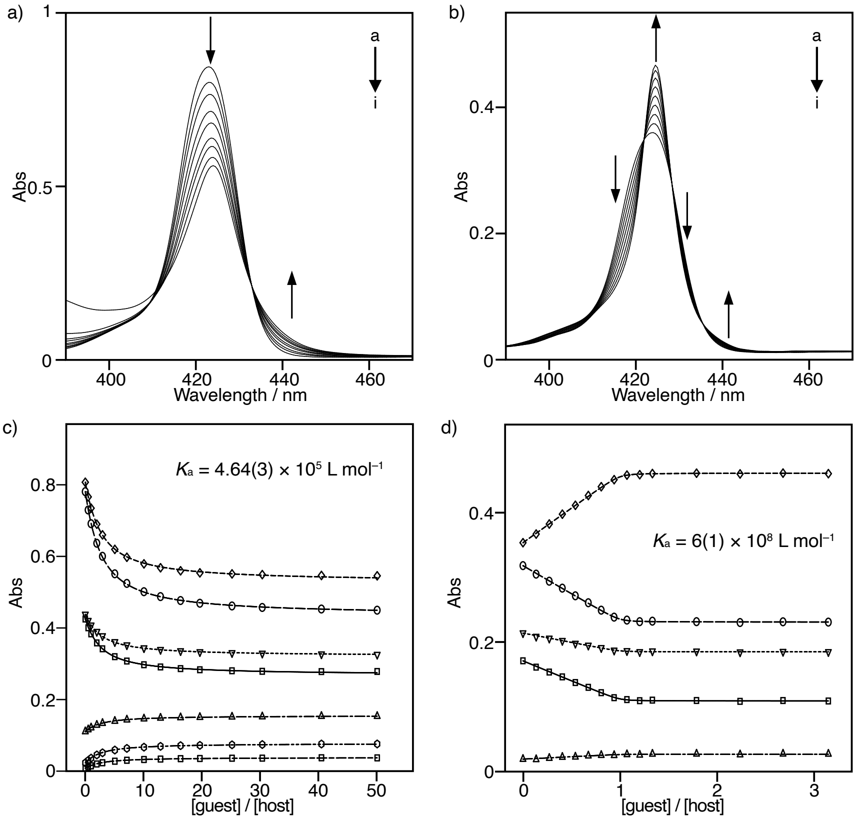

Figure S15. (a) UV-Vis absorption spectra of **9Zn** (1.0 × 10^{-6} mol L^{-1}) in the presence of **G1** (a-i: 0.0, 0.50, 1.0, 2.0, 3.0, 5.1, 7.1, 13, and 50 × 10^{-6} mol L^{-1}) in chloroform at 295 K. (b) UV-Vis absorption spectra of **9Zn** (5.0 × 10^{-7} mol L^{-1}) in the presence of **G2** (a-i: 0.0, 0.07, 0.14, 0.20, 0.27, 0.34, 0.40, 0.47, 0.55, and 1.6 × 10^{-6} mol L^{-1}) in chloroform at 295 K. Plots of Abs of (c) **G1** and (d) **G2** against [guest] / [host] and the fitting curves obtained by 1:1 fitting model.

2.3 FL studies of binding behavior of **1Zn**

A titration was performed wherein the concentration of a host solution was fixed while varying the concentration of guests. During the course of the titration, fluorescence changes were measured from 500 nm to 800 nm at a fixed excitation wavelength of 520 nm.

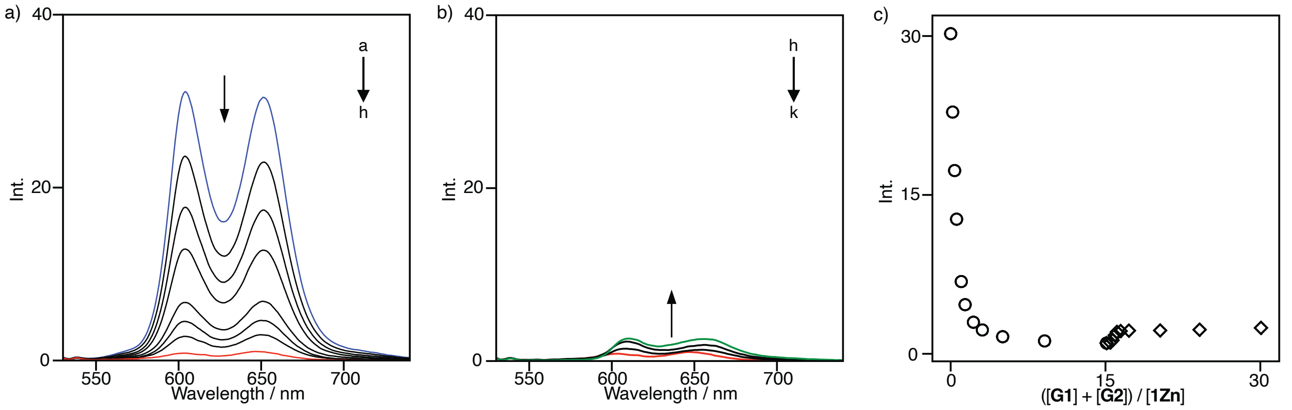

Figure S16. (a, b) Emission spectral changes (λ_{ex} = 520 nm) of **1Zn** (1.0×10^{-6} mol L^{-1}) upon addition of **G1** (a–h: 0.0, 0.19, 0.38, 0.57, 1.0, 1.4, 2.2, and 15 × 10^{-5} mol L^{-1}) and then **G2** (h–k: 0.0, 0.58, 1.0 and 15 × 10^{-5} mol L^{-1}) at 295 K in chloroform. (c) Plots of Intensity at 650 nm of **1Zn** versus ([**G1**] + [**G2**]) / [**1Zn**] upon the stepwise addition of **G1** (circle) and **G2** (rhombus).

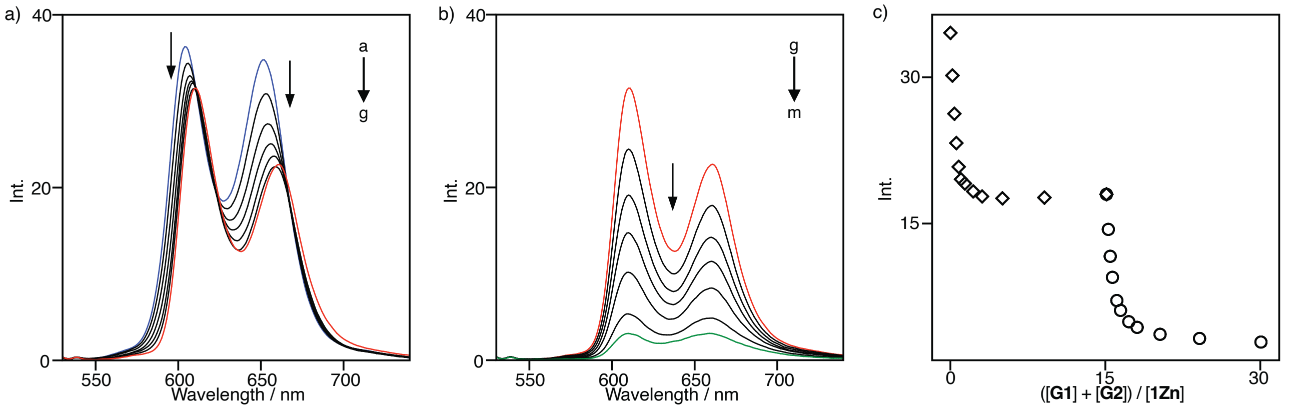

Figure S17. (a, b) Emission spectral changes (λ_{ex} = 520 nm) of **1Zn** (1.0×10^{-6} mol L^{-1}) upon addition of **G2** (a–g: 0.0, 0.19, 0.38, 0.57, 1.0, 2.2, and 15 × 10^{-5} mol L^{-1}) and then **G1** (g–m: 0.0, 0.19, 0.37, 0.58, 1.0, 3.0 and 15 × 10^{-5} mol L^{-1}) at 295 K in chloroform. (c) Plots of Intensity at 650 nm of **1Zn** versus ([**G1**] + [**G2**]) / [**1Zn**] upon the stepwise addition of **G1** (circle) and **G2** (rhombus).

3 Formation of ternary and septenary supramolecular complexes

3.1 NMR studies of ternary complex

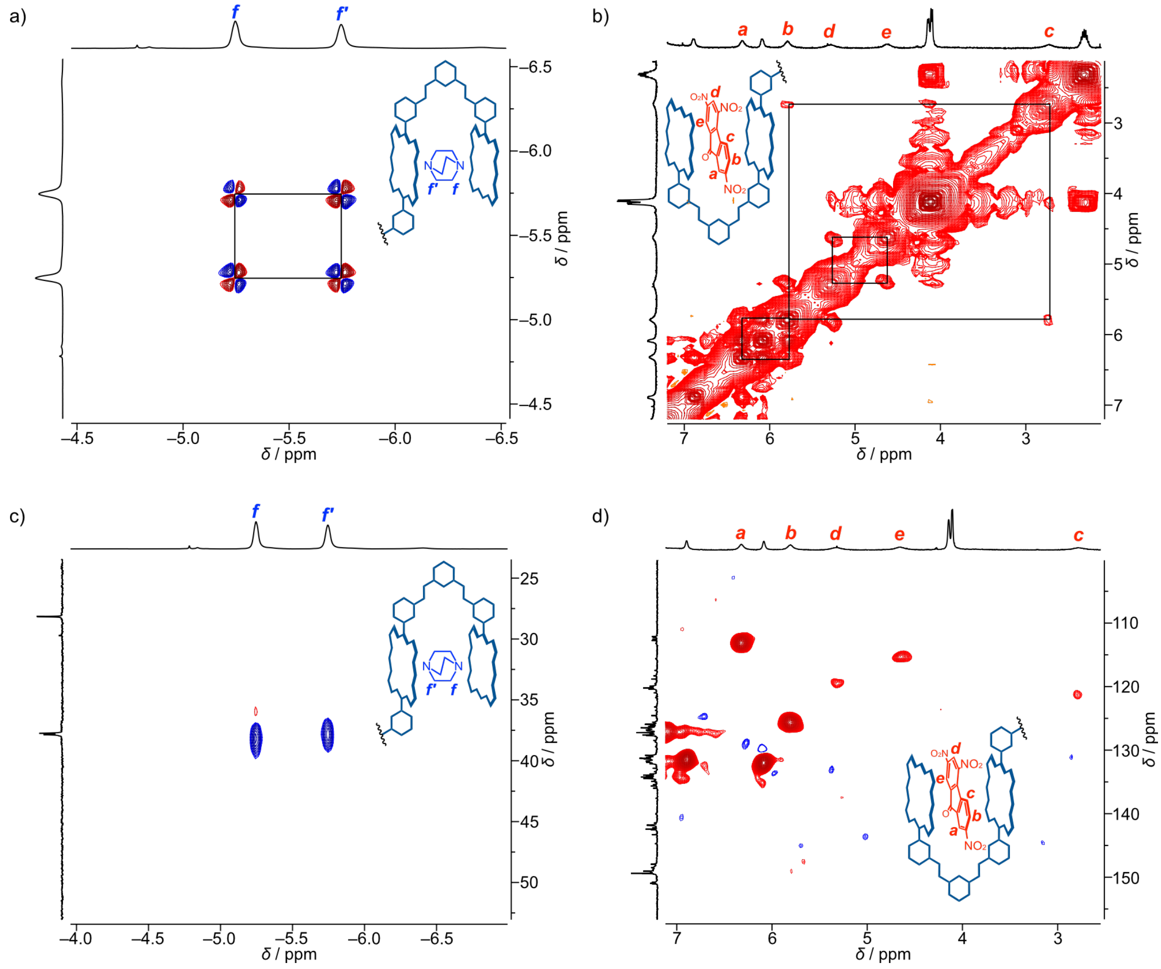

Figure S18. Selected regions of (a) DQF COSY (700MHz, 298 K), (b) gCOSY (400 MHz, 298 K), and (c,d) HSQC (700 MHz, 298K) spectra of a 1:1:1 mixture of **1Zn** (10 mmol L^{-1}), **G1** (10 mmol L^{-1}), and **G2** (10 mmol L^{-1}) in chloroform-*d*.

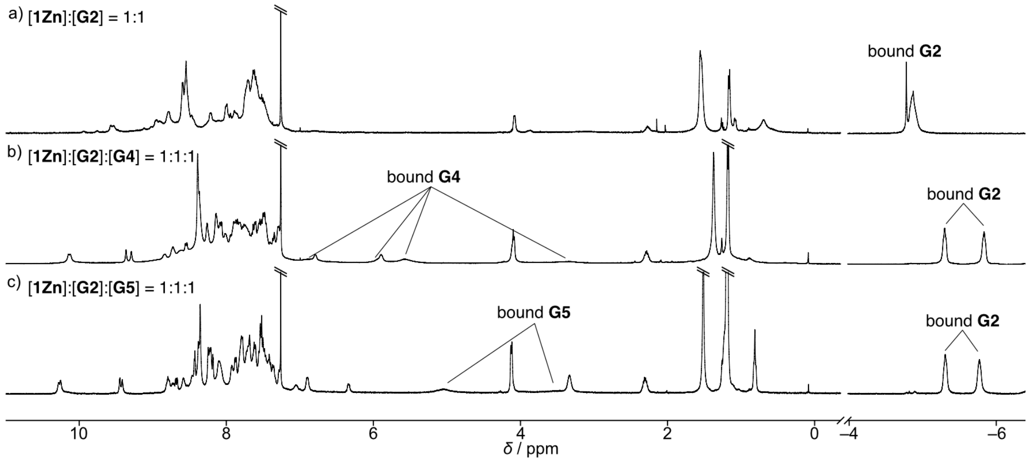

Figure S19. ^{1}H NMR spectra of (a) a 1:1 mixture of **1Zn** (10 mmol L^{-1}) and **G2** (10 mmol L^{-1}), (b) a 1:1:1 mixture of **1Zn** (10 mmol L^{-1}), **G2** (10 mmol L^{-1}), and **G4** (10 mmol L^{-1}), and (c) a 1:1:1 mixture of **1Zn** (10 mmol L^{-1}), **G2** (10 mmol L^{-1}), and **G5** (10 mmol L^{-1}) in chloroform-*d*.

3.2 DOSY experiments of ternary complex

^{1}H DOSY spectra were measured on a Bruker Ascend 400 spectrometer. Compounds were dissolved in chloroform-*d*, and the sample solutions were placed in a 3 mm NMR sample tube. The pulse-field gradient diffusion NMR spectra were collected using a ledbpgp2s pulse sequence. The pulsed-field gradient strength was arrayed from 0.963 to 47.187 mT m^{-1} with a pulse gradient time of 1.4 ms and a diffusion time of 100 ms. The data were processed using the MestReNova program. The signal intensity as a function of the pulse-field gradient strength was fitted to the Stejskal-Tanner equation to determine the diffusion coefficients (*D*s). The 2D DOSY maps were generated using the Bayesian transformation.

The relative molecular sizes of the septenary complexes were estimated by comparing the diffusion coefficients of the ternary complex and its aggregate based on the following equation: $(D_{\text{ternary}}/D_{\text{septenary}})^3$.

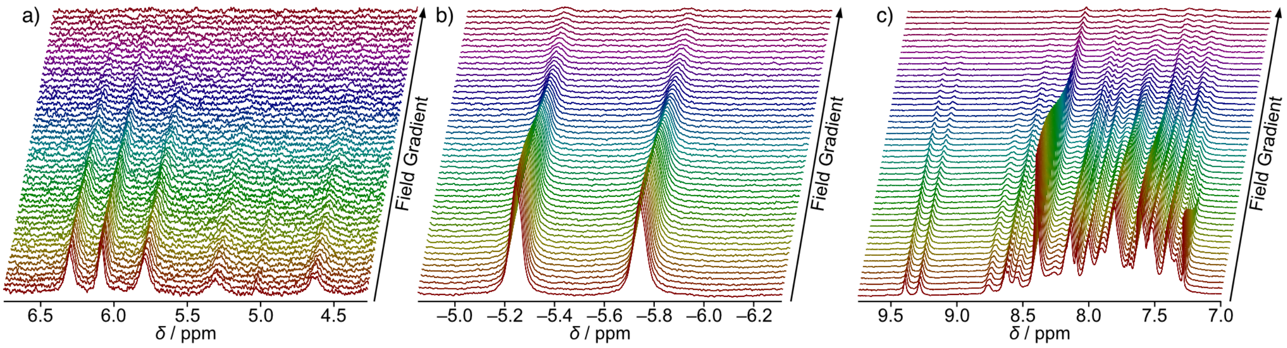

Figure S20. Stacked ^{1}H NMR spectra obtained from the DOSY experiments (400 MHz) of a 1:1:1 mixture of **1Zn** (10 mmol L^{-1}), **G1** (10 mmol L^{-1}), and **G2** (10 mmol L^{-1}) in chloroform-*d*, showing (a) the aromatic protons of **G1**, (b) the methylene protons of **G2**, and the aromatic protons of **1Zn**.

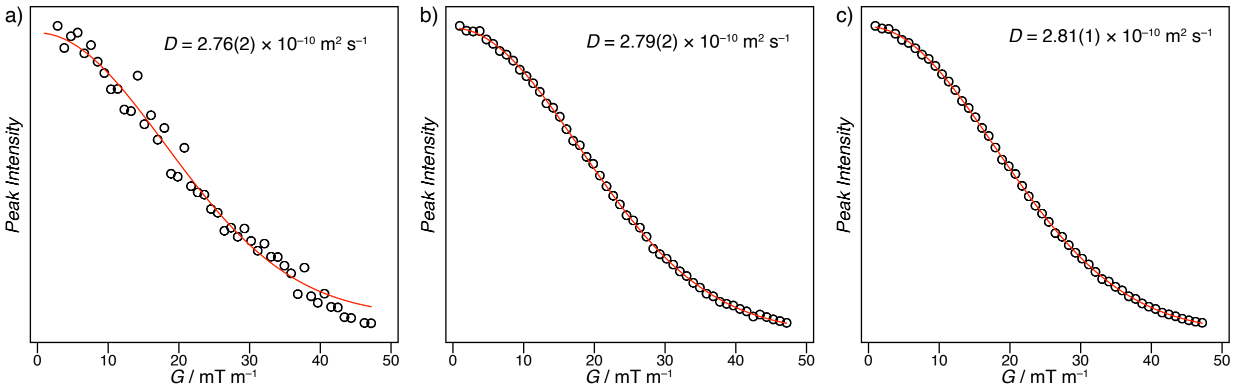

Figure S21. Diffusion decay curves of DOSY spectra obtained from the solutions of a 1:1:1 mixture of **1Zn** (10 mmol L^{-1}), **G1** (10 mmol L^{-1}), and **G2** (10 mmol L^{-1}) in chloroform-*d*, showing (a) the aromatic protons of **G1** at 6.28 ppm, (b) the methylene protons of **G2** at –5.27 ppm, and the aromatic protons of **1Zn** at 8.40 ppm.

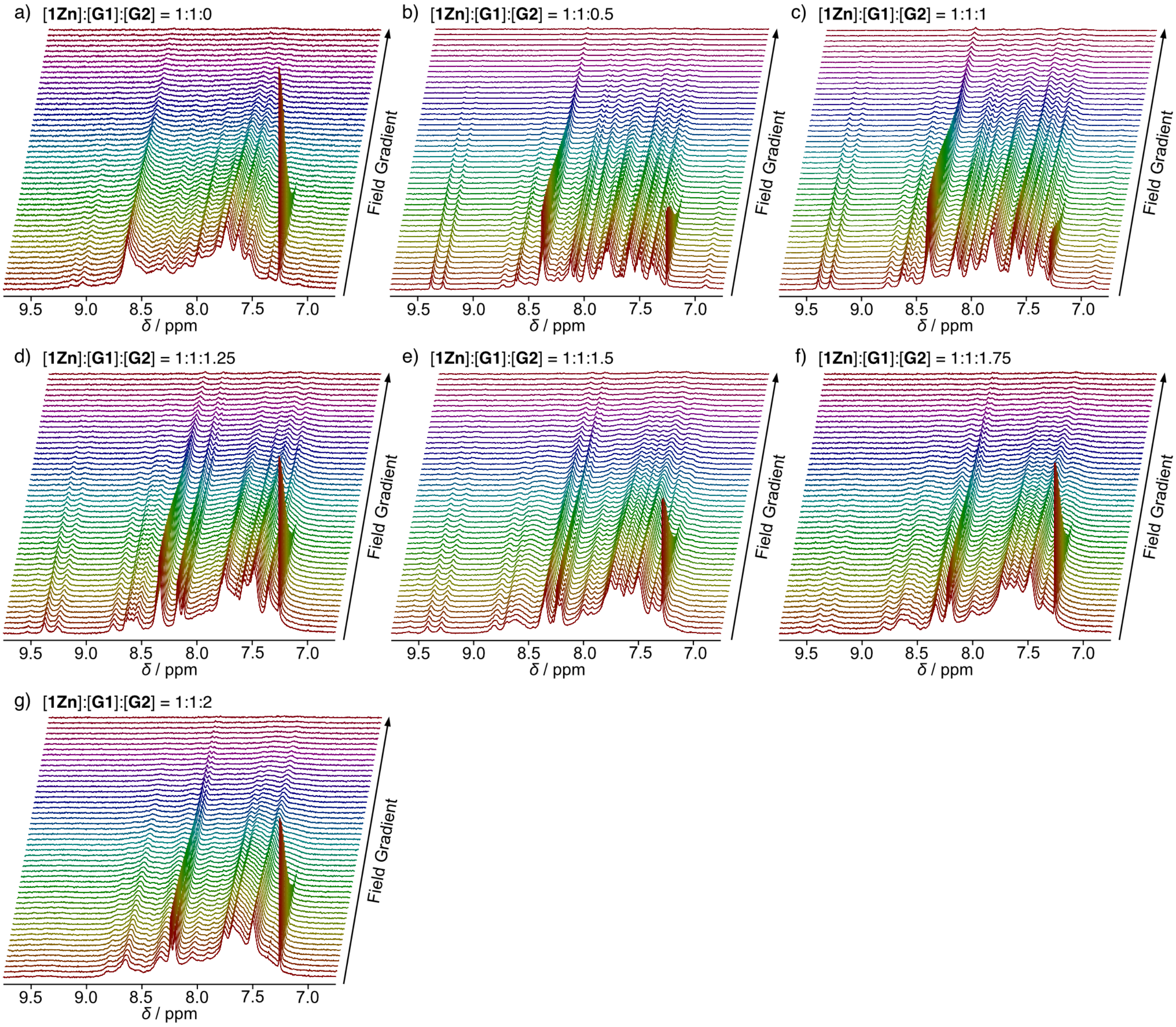

Figure S22. Stacked ^{1}H NMR spectra obtained from the DOSY experiments (400 MHz) of a 1:1 mixture of **1Zn** (10 mmol L^{-1}) and **G1** (10 mmol L^{-1}) in the presence of **G2** (0, 5.0, 10.0, 12.5, 15.0, 17.5, 20.0 mmol L^{-1}) in chloroform-*d*, showing the aromatic protons of **1Zn**.

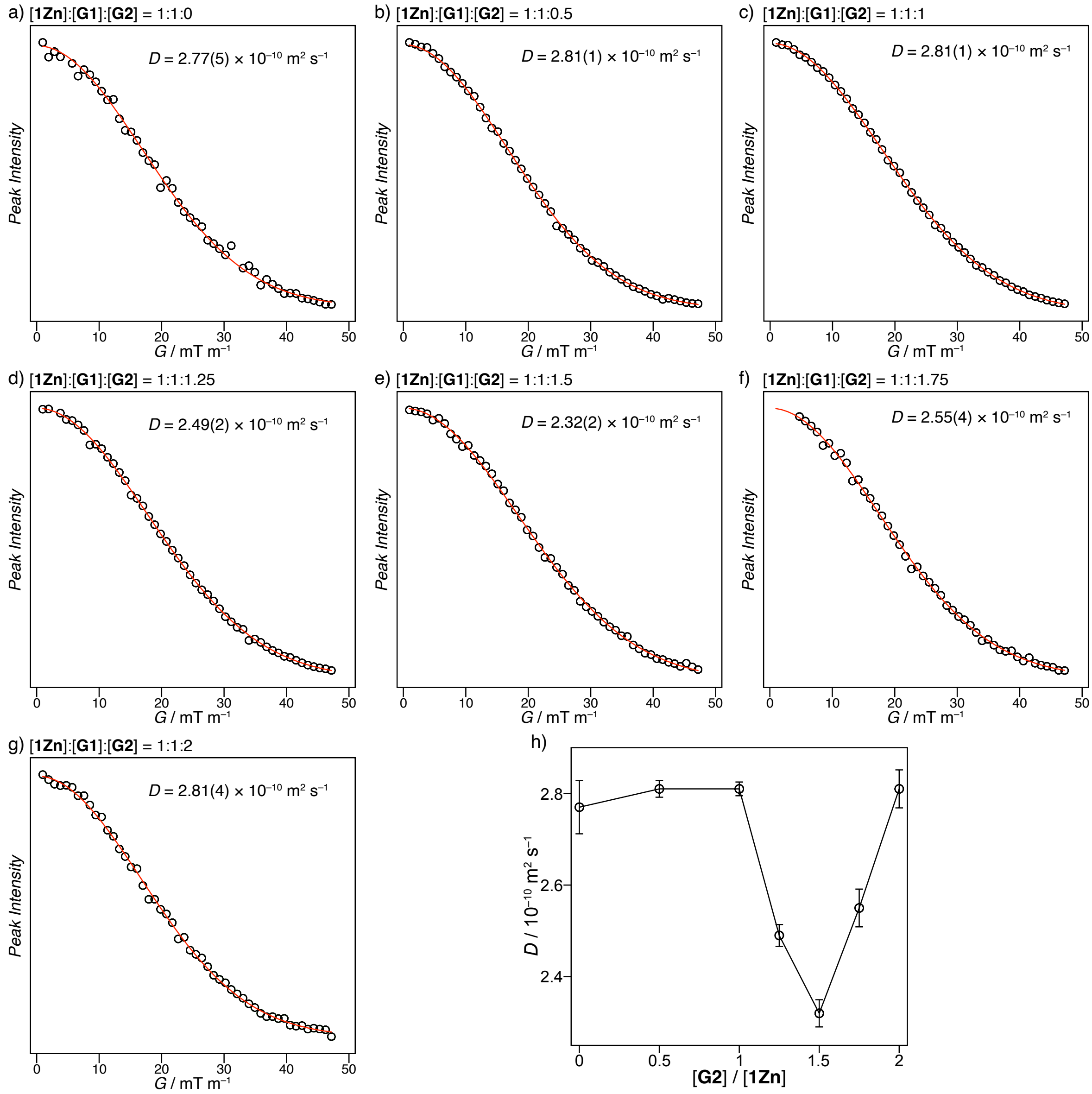

Figure S23. Diffusion decay curves of DOSY spectra obtained from the solutions of a 1:1 mixture of **1Zn** (10 mmol L^{-1}) and **G1** (10 mmol L^{-1}) in the presence of **G2** (0, 5.0, 10.0, 12.5, 15.0, 17.5, 20.0 mmol L^{-1}) in chloroform-*d*, showing the aromatic protons of **1Zn** at (a) 8.71, (b) 8.40, (c) 8.40, (d) 8.40, (e) 8.41, (f) 8.23, and (g) 8.26 ppm. (h) Plots of diffusion coefficients against [**G2**].

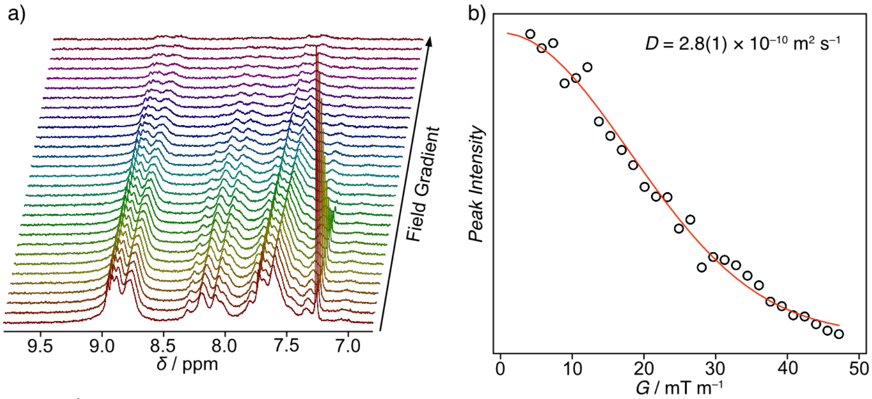

Figure S24. (a) Stacked ^{1}H NMR spectra obtained from the DOSY experiments (400 MHz) of **1Zn** (10 mmol L^{-1}) in chloroform-*d*, showing the aromatic protons of **1Zn**. (b) Diffusion decay curves of DOSY spectra at 7.64 ppm.

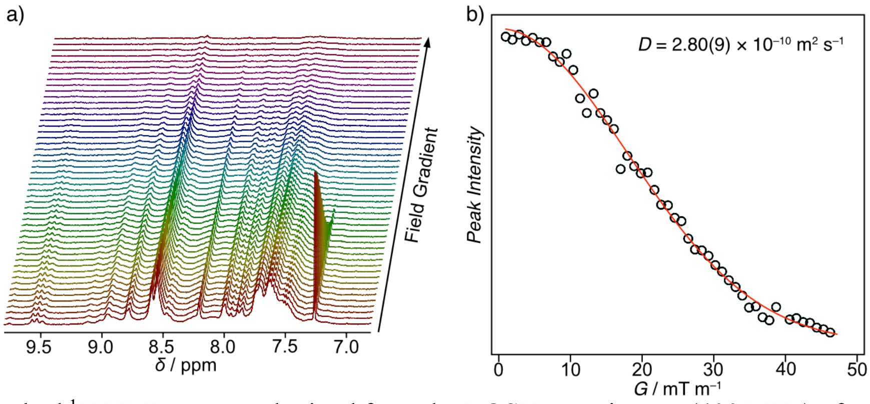

Figure S25. (a) Stacked ^{1}H NMR spectra obtained from the DOSY experiments (400 MHz) of a 1:1 mixture of **1Zn** (10 mmol L^{-1}) and **G2** (10 mmol L^{-1}) in chloroform-*d*, showing the aromatic protons of **1Zn**. (b) Diffusion decay curves of DOSY spectra at 8.79 ppm.

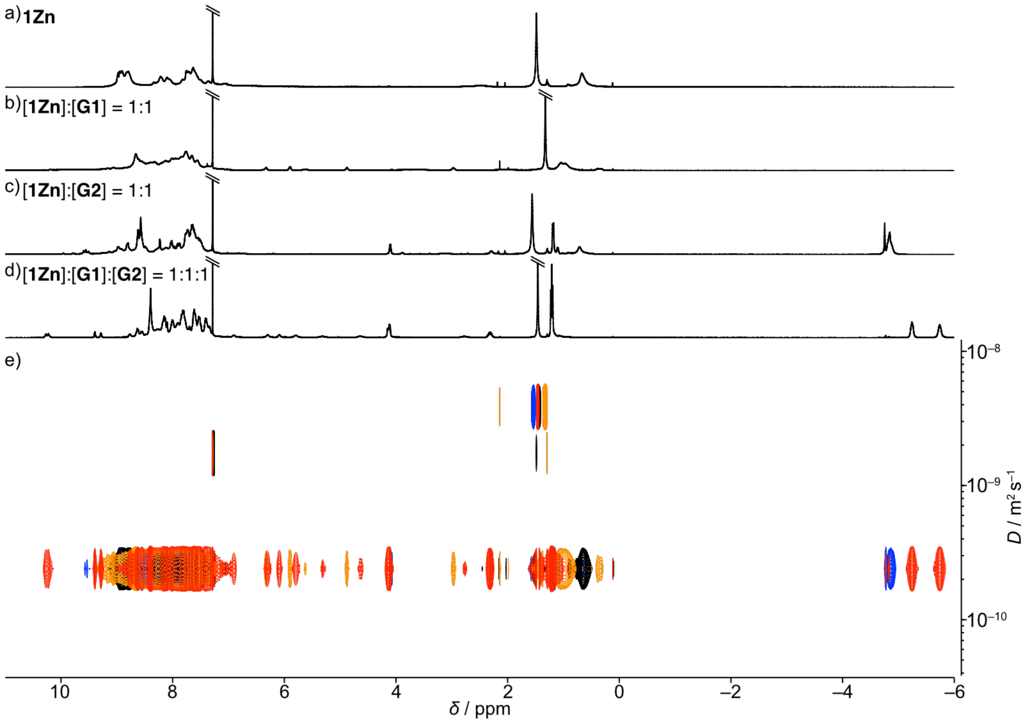

Figure S26. ^{1}H NMR spectra of (a) **1Zn** (10 mmol L^{-1}), (b) a 1:1 mixture of **1Zn** (10 mmol L^{-1}) and **G1** (10 mmol L^{-1}), (c) a 1:1 mixture of **1Zn** (10 mmol L^{-1}) and **G2** (10 mmol L^{-1}), and (d) a 1:1:1 mixture of **1Zn** (10 mmol L^{-1}), **G1** (10 mmol L^{-1}), and **G2** (10 mmol L^{-1}) in chloroform-*d*. (e) 2D DOSY spectra of **1Zn** (black line), a 1:1 mixture of **1Zn** and **G1** (orange line), a 1:1 mixture of **1Zn** and **G2** (blue line), and a 1:1:1 mixture of **1Zn**, **G1**, and **G2** (red line).

3.3 ESI-MS study of ternary and septenary supramolecular complex

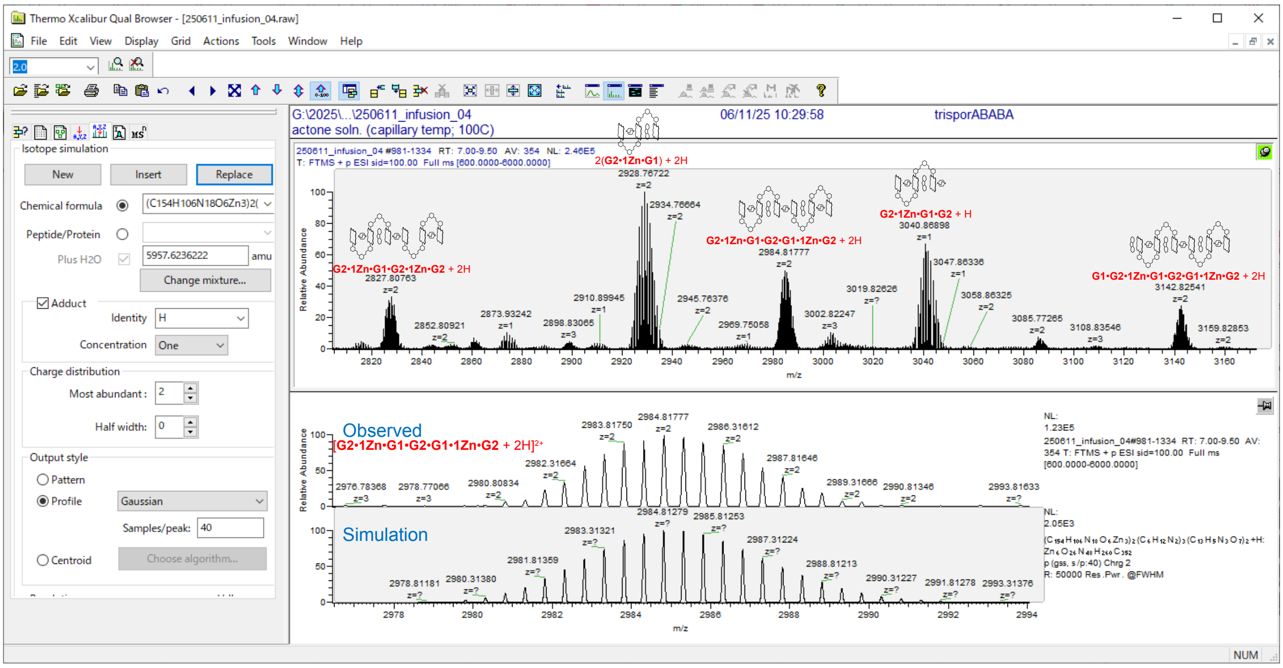

Figure S27. ESI-MS spectrum of a mixture of **1Zn**, **G1**, and **G2**.

3.4 X-ray Crystallography

X-ray-quality single crystals were grown via liquid-liquid diffusion from hexane into chloroform solution containing **1Zn**, **G1**, and **G2** in a 1:4:4 molar ratio. X-ray crystallographic data were obtained at 100 K with a Rigaku XtaLAB Synergy-S Dual Source diffractometer equipped with a PhotonJet Cu-microfocus source (λ = 1.5418 Å) and a HyPix-6000HE detector. Data reduction and processing were performed with CrysAlisPro. The crystals were solved by intrinsic phasing with the SHELXT. Successive differential Fourier syntheses and full-matrix least-squares procedures were applied for refinement with the SHELXL in the Olex2 graphical user interface. Anisotropic thermal factors were evaluated for all atoms except for the hydrogen atoms. The hydrogen atoms were geometrically produced.

Crystal data. **G2•1Zn•G1•G2•G1•1Zn•G2**: Formula $C_{183}H_{136}Cl_{21}N_{24}O_{13}Zn_3$, F.W.= 3819.71, monoclinic, $P2_1/n$ (#14), a = 20.2148(5) Å, b = 40.5922(7) Å, c = 24.9002(7) Å, α = 90°, β = 107.445(3)°, γ = 90°, V = 19492.4(9) Å^3, d_{calc} = 1.302 g cm^{-3}, Z = 4, crystal size = 0.08 × 0.04 × 0.04 mm^3, dark red block, Temp. = 100 K, $2\theta_{max}$ = 103.67°, μ = 3.582 mm^{-1}, total/unique/observed reflections = 93515/21307/9693, R_{int} = 0.1642, $R1(F_o)$ = 0.1172, $wR2(F_o^2)$ = 0.3167, GoF = 1.059, $\Delta\rho_{max,min}$ = 0.923, –0.615 eÅ^{-3}, CCDC = 2440096

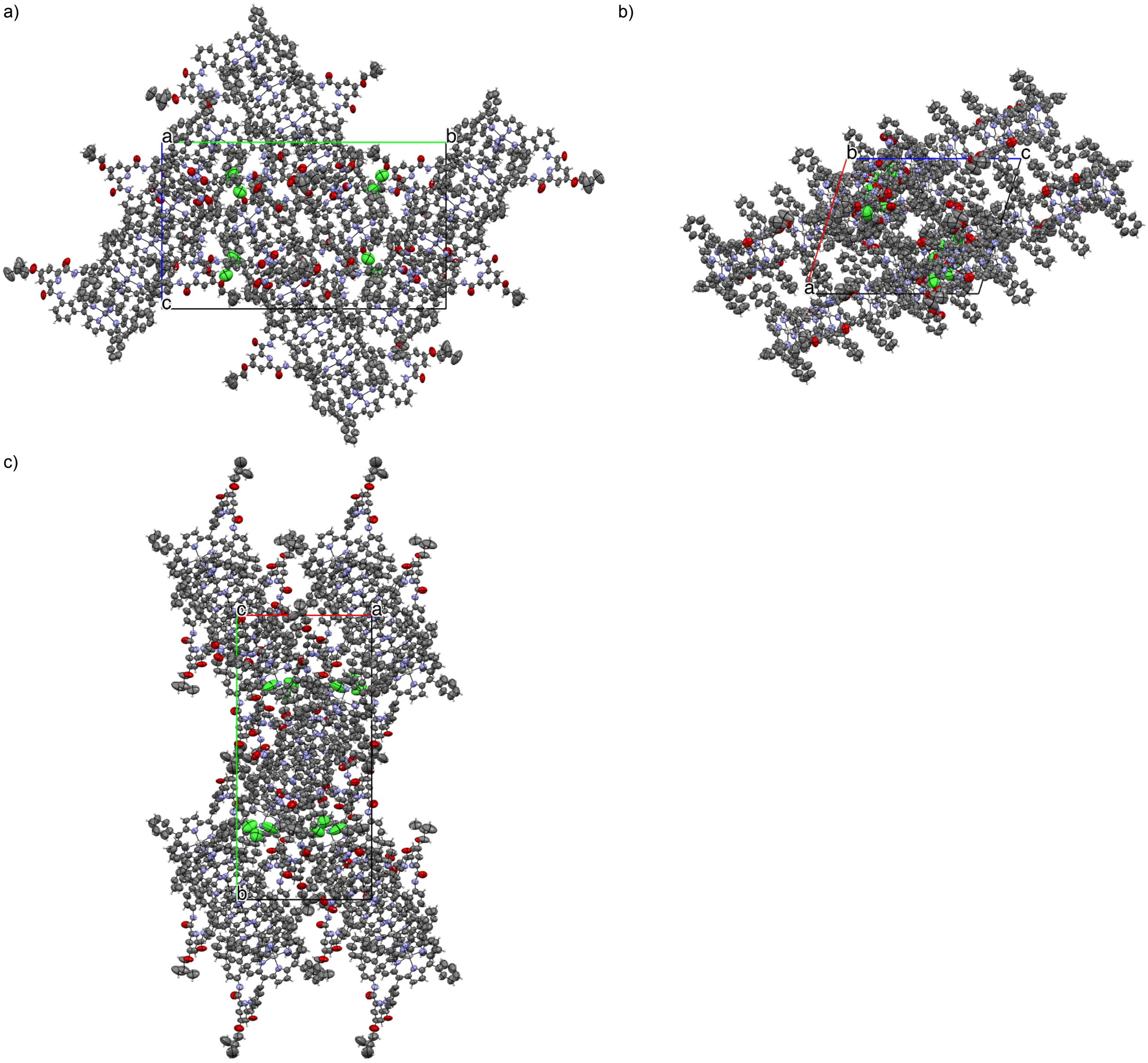

Figure S28. ORTEP drawing of the X-ray crystal structure of **G2•1Zn•G1•G2•G1•1Zn•G2**. Views along (a) a-, (b) b-, and (c) c-axes. Ellipsoids are shown at 50%.

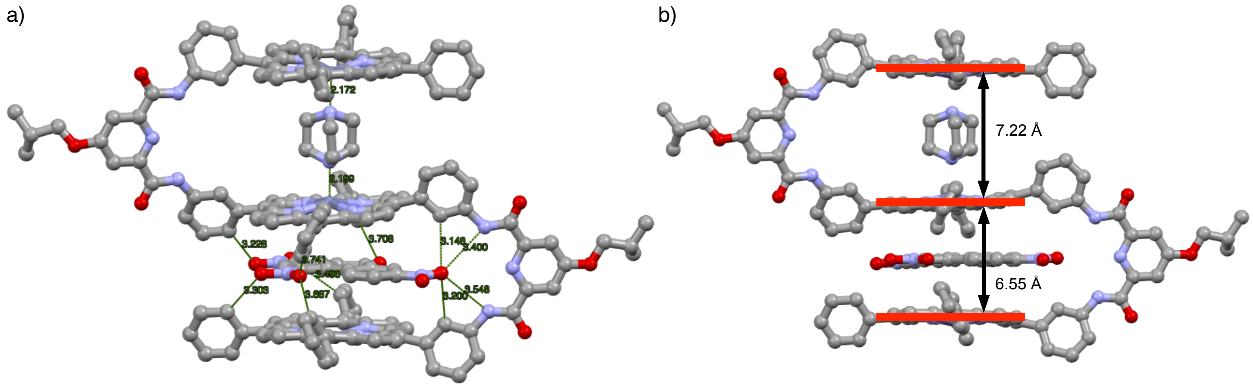

Figure S29. The partial X-ray crystal structure of **G2•1Zn•G1•G2•G1•1Zn•G2**, showing (a) the intermolecular N···O and C···O distances between **1Zn** and **G1** and the interatomic Zn···N distances between **1Zn** and **G2** and (b) the interlayer porphyrin distances.

4 Formation of septenary supramolecular complex G2•1Zn•G1•G3•G1•1Zn•G2

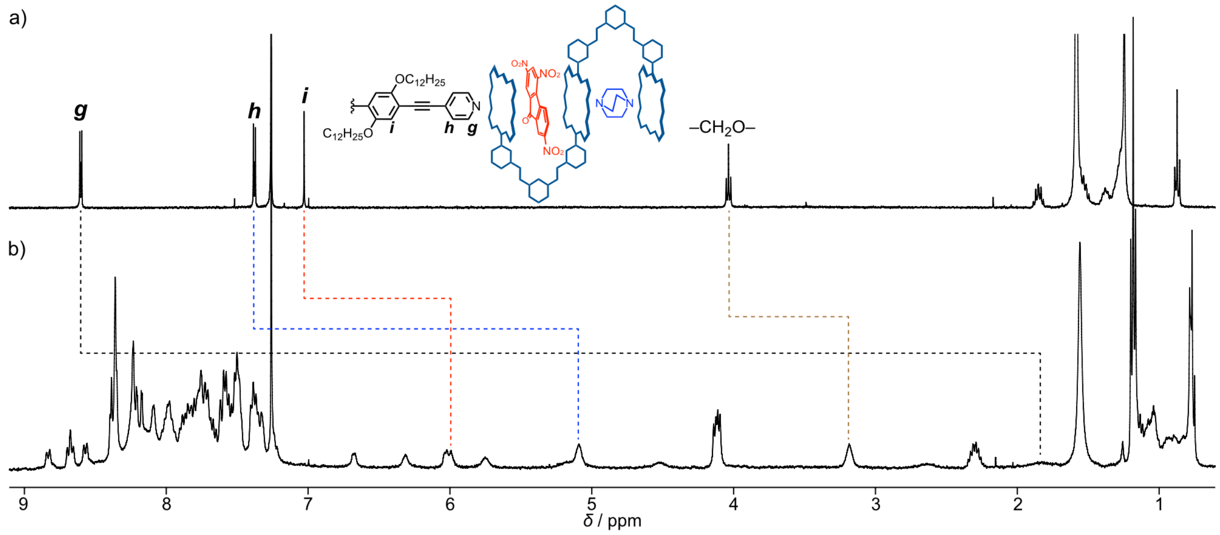

Figure S30. ^{1}H NMR spectra of (a) **G3** and (b) a 1:1:1:0.5 mixture of **1Zn**, **G1**, **G2**, and **G3**.

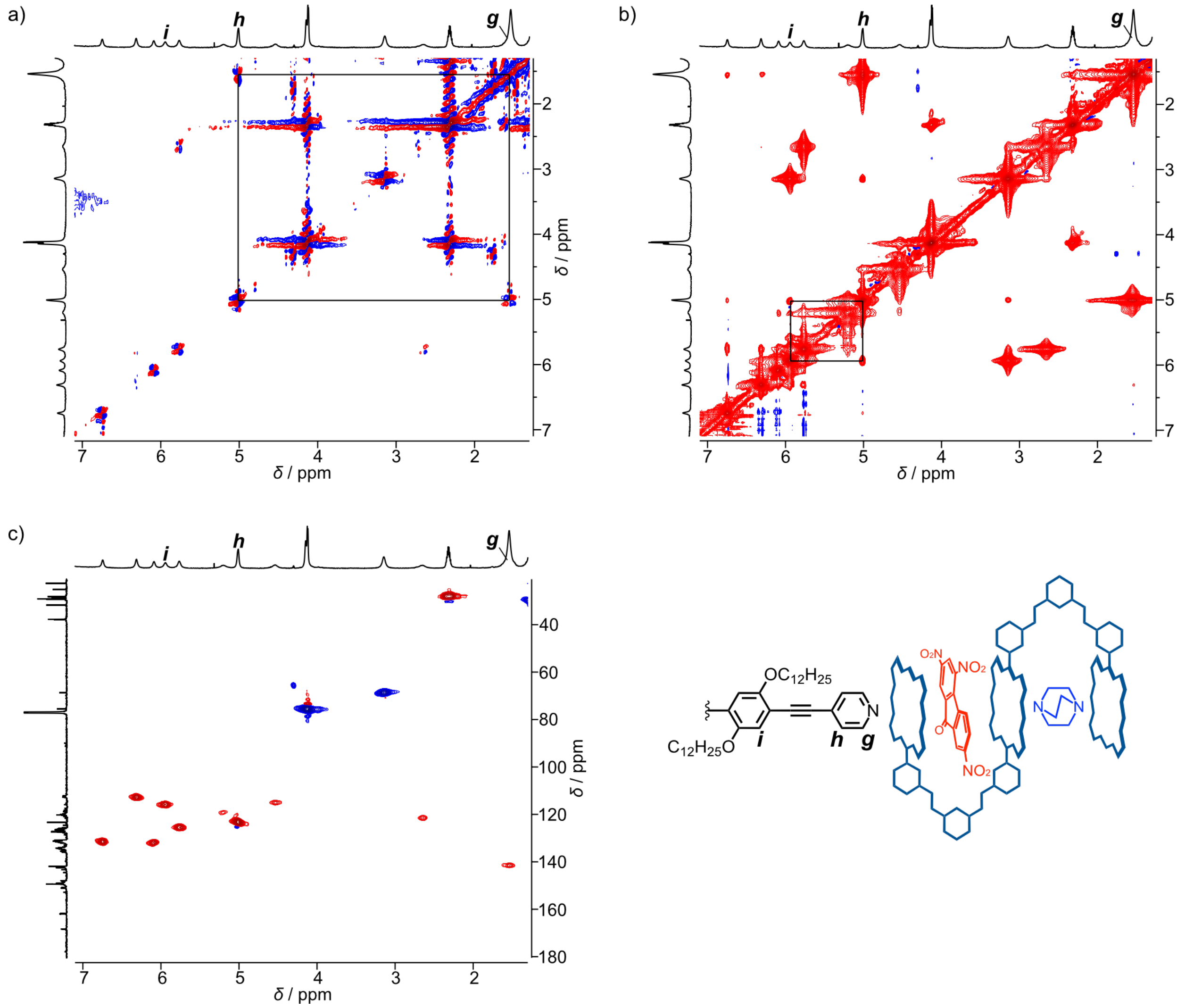

Figure S31. Selected regions of (a) DQF COSY (700MHz, 298 K), (b) NOESY (700 MHz, 298 K), and (c) HSQC (700 MHz, 298K) spectra of a 1:1:1:0.5 mixture of **1Zn**, **G1**, **G2**, and **G3** in chloroform-*d*.

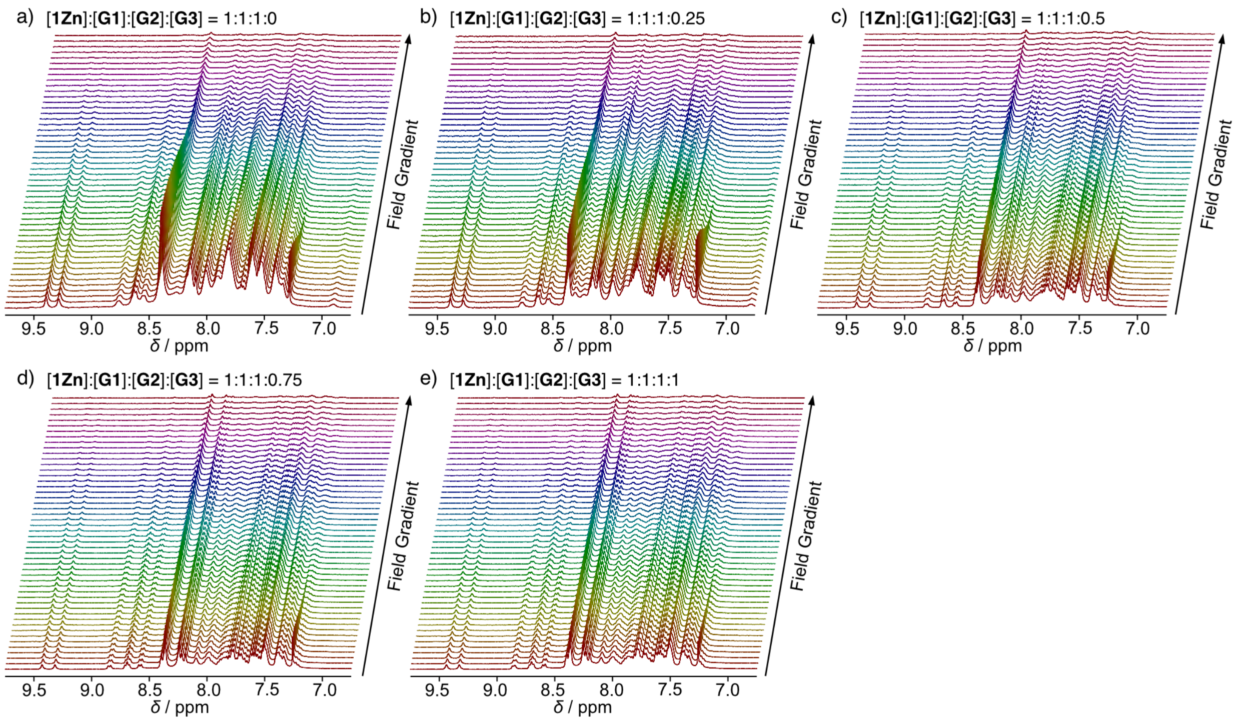

Figure S32. Stacked ^{1}H NMR spectra obtained from the DOSY experiments (400 MHz) of a 1:1:1 mixture of **1Zn** (10 mmol L^{-1}),**G1** (10 mmol L^{-1}), and **G2** (10 mmol L^{-1}) in the presence of **G3** (0, 2.5, 5.0, 7.5 10.0 mmol L^{-1}) in chloroform-*d*, showing the aromatic protons of **1Zn**.

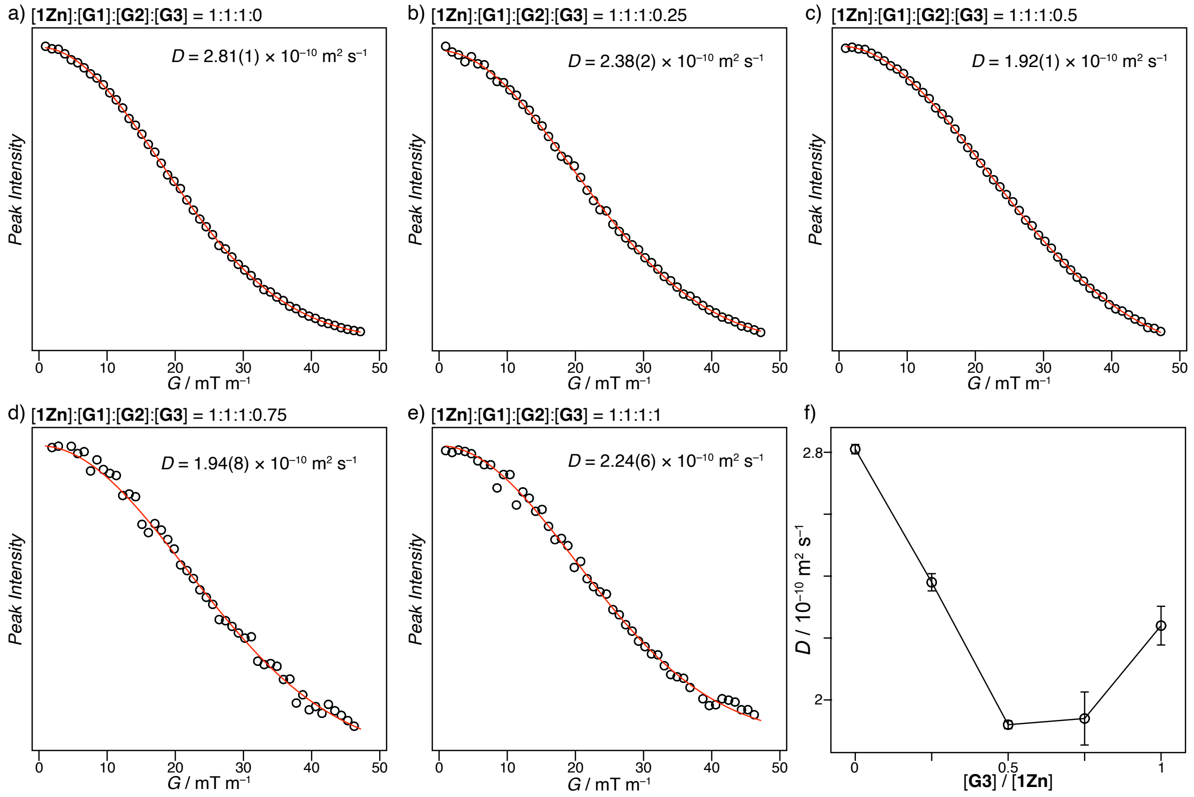

Figure S33. Diffusion decay curves of DOSY spectra obtained from the solutions of a 1:1:1 mixture of **1Zn** (10 mmol L^{-1}), **G1** (10 mmol L^{-1}), and **G2** (10 mmol L^{-1}) in the presence of **G3** (0, 2.5, 5.0, 7.5 10.0 mmol L^{-1}) in chloroform-*d*, showing the aromatic protons of **1Zn** at (a) 8.40, (b) 8.38, (c) 8.36, (d) 8.84, and (e) 8.20 ppm. (f) Plots of diffusion coefficients against [**G3**].

5 DFT calculations

DFT calculations were performed using Gaussian 16 Rev. C01 program.[S9] Molecular geometries were optimized at the M06-2X/6-31G(d,p) level for **G1•1Zn•G2** and the M06-2X/6-31G level for **G2•1Zn•G1•G2•G1•1Zn•G2**. Frequency calculations were performed at the same level to identify the minima. The GaussView 6 program was used to visualize the molecular structures.

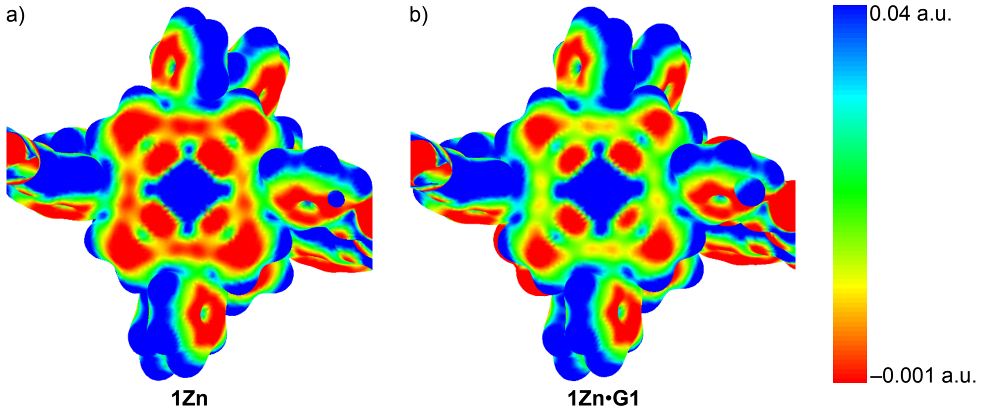

Figure S34. Molecular electrostatic potential surfaces of the central porphyrin planes in (a) **1Zn** and (b) **1Zn•G1**.

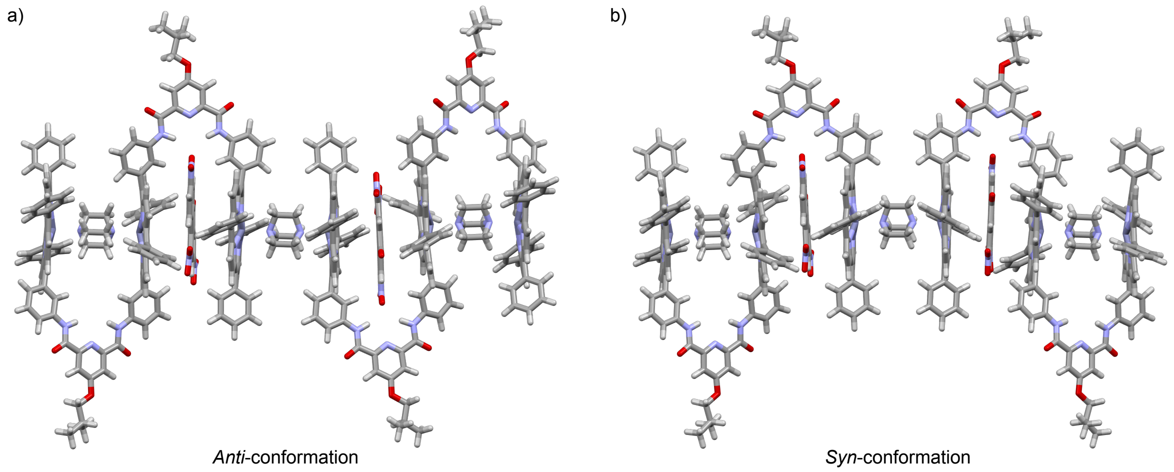

Figure S35. Optimized geometric structures of (a) *anti* and (b) *syn* conformations of **G2•1Zn•G1•G2•G1•1Zn•G2**. The optimizations were carried out by DFT calculations at M06-2X/6-31G level.

Table S1. Cartesian coordinates of **G1•1Zn•G2**.

Number of imaginary frequencies = 0

Total energy (hartree) = –14235.085966

Center Number	Atomic Number	Atomic Type	Coordinates (Angstroms) X	Y	Z
1	30	0	0.427739	-0.245237	0.043461
2	30	0	-4.128128	-5.270651	0.391868
3	30	0	4.000750	5.645938	-0.242930
4	7	0	-5.365555	-4.247897	1.662055
5	7	0	-2.882545	-6.277111	-0.884625
6	7	0	1.061769	-1.364930	-1.585709
7	7	0	-2.804628	-5.561179	1.910908
8	7	0	-0.621397	0.288431	1.772257
9	7	0	-1.268756	0.299977	-1.050356
10	7	0	-5.348847	-4.830818	-1.173386
11	7	0	5.113979	5.491590	-1.989445
12	7	0	5.700402	5.129392	0.833235
13	7	0	10.191337	-2.071092	-0.493778
14	6	0	10.201799	-3.376718	-0.186014
15	6	0	11.342554	-4.087073	0.145112
16	1	0	11.272370	-5.141554	0.380386
17	6	0	12.570163	-3.417697	0.153864
18	6	0	12.585745	-2.065748	-0.187184
19	1	0	13.482922	-1.463166	-0.232766
20	6	0	11.373426	-1.454452	-0.495706
21	7	0	1.639439	-1.429444	1.247784
22	7	0	3.185222	6.414994	1.513053
23	7	0	-6.866415	3.710173	0.593717
24	1	0	-7.225318	2.770787	0.450327
25	7	0	-9.423331	2.938780	0.081134
26	6	0	-9.187227	4.244091	0.195714
27	6	0	-10.153993	5.235806	0.046940
28	1	0	-9.847766	6.266417	0.166298
29	6	0	-11.458644	4.838241	-0.244647
30	6	0	-11.725928	3.470935	-0.366651
31	1	0	-12.720871	3.107223	-0.591025
32	6	0	-10.684095	2.576172	-0.193729
33	6	0	-6.586283	-3.715197	1.344175
34	7	0	-9.956832	0.280620	-0.079410
35	1	0	-9.092580	0.742138	0.190087
36	7	0	2.599435	6.764104	-1.305378
37	8	0	-7.550480	5.899593	0.676858
38	7	0	7.813308	-3.424613	-0.573678
39	1	0	7.976982	-2.434134	-0.695712
40	6	0	-3.041275	-6.434838	-2.234702
41	6	0	0.568672	-1.297046	-2.856170

42	6	0	-5.137451	-5.119666	-2.496191
43	8	0	12.524640	0.558043	-1.008177
44	6	0	-7.164837	-3.704297	0.066450
45	8	0	-12.493793	5.675865	-0.417082
46	6	0	-0.354595	-0.080207	3.063649
47	8	0	8.906629	-5.354166	0.007030
48	6	0	2.961137	-2.530234	-0.522281
49	7	0	10.232344	0.600345	-1.051628
50	1	0	9.434538	0.009096	-0.859204
51	6	0	-0.547356	-0.542030	-3.267557
52	6	0	-1.765576	-6.985237	-0.530648
53	6	0	1.681292	-1.448305	2.612032
54	6	0	-6.202903	-4.551481	-3.292486
55	1	0	-6.257227	-4.584658	-4.370120
56	6	0	4.639821	5.692609	-3.254056
57	6	0	6.838058	4.577950	0.318960
58	8	0	-12.132434	0.745269	-0.643859
59	6	0	5.823219	5.106707	2.192642
60	6	0	-1.371222	0.217552	-2.412693
61	6	0	-2.182912	1.233583	-0.636984
62	6	0	-4.105224	-6.195362	-4.471656
63	6	0	-3.074394	-5.786000	-5.323632
64	1	0	-2.225202	-5.244422	-4.919959
65	6	0	-3.119221	-6.082748	-6.682954
66	1	0	-2.310909	-5.753262	-7.327686
67	6	0	-4.192729	-6.794712	-7.210368
68	1	0	-4.226666	-7.025851	-8.270131
69	6	0	-5.221259	-7.213457	-6.369732
70	1	0	-6.057714	-7.777200	-6.770247
71	6	0	-5.177303	-6.916674	-5.011507
72	1	0	-5.975281	-7.247358	-4.353128
73	6	0	-2.919679	-5.086134	3.188100
74	6	0	2.147997	-2.194208	-1.620616
75	6	0	-1.697364	-6.371857	1.883070
76	6	0	7.724171	4.193619	1.398659
77	1	0	8.704289	3.756431	1.276933
78	6	0	-0.025471	-7.957829	0.925077
79	6	0	-0.172021	-9.336678	0.733439
80	1	0	-1.153674	-9.734418	0.492733
81	6	0	0.922067	-10.188357	0.846464
82	1	0	0.790975	-11.255465	0.698192
83	6	0	2.180617	-9.673103	1.147890
84	1	0	3.035334	-10.336529	1.231345
85	6	0	2.336247	-8.303486	1.341271
86	1	0	3.313964	-7.891436	1.570627
87	6	0	1.240644	-7.450177	1.234605
88	1	0	1.366205	-6.383797	1.391809
89	6	0	2.712744	-2.154472	0.810222
90	6	0	-4.078768	-5.883564	-3.010804
91	6	0	-6.541140	-4.163595	-1.102827
92	6	0	-3.308914	2.786597	0.974147
93	6	0	-2.807848	3.913510	1.640793
94	1	0	-1.761624	3.943068	1.930831
95	6	0	-3.643602	4.986290	1.925992
96	1	0	-3.241016	5.858261	2.431989
97	6	0	-4.990494	4.963997	1.578652
98	1	0	-5.640596	5.795842	1.808943
99	6	0	-5.505249	3.839353	0.922681
100	6	0	-4.655890	2.770187	0.608891
101	1	0	-5.054837	1.916335	0.071458
102	6	0	-7.077274	-3.976000	-2.430784
103	1	0	-7.994132	-3.452021	-2.658466
104	6	0	-7.781820	4.711504	0.517079
105	6	0	-8.527776	-3.108545	-0.086888
106	6	0	-8.679457	-1.729852	-0.023889
107	1	0	-7.805723	-1.111688	0.124673
108	6	0	-9.929071	-1.120574	-0.171252
109	6	0	-11.055547	-1.919360	-0.399950
110	1	0	-12.026097	-1.461162	-0.521737
111	6	0	-10.895219	-3.301586	-0.474741
112	1	0	-11.770131	-3.918371	-0.654357
113	6	0	-9.651008	-3.906029	-0.322128
114	1	0	-9.543476	-4.984271	-0.382644
115	6	0	-3.960606	-4.282612	3.687338
116	6	0	2.522040	6.878104	-2.663179
117	6	0	7.127273	4.417670	-1.047809
118	6	0	-5.125178	-3.942259	2.973224
119	6	0	-2.408283	1.115720	-2.876998
120	1	0	-2.698226	1.251676	-3.908395
121	6	0	-1.093100	-6.409852	3.195038
122	1	0	-0.215787	-6.982838	3.456389
123	6	0	-2.380253	1.652814	0.691043
124	6	0	6.301929	4.831915	-2.109277
125	6	0	1.359742	-2.141949	-3.729999
126	1	0	1.166224	-2.312802	-4.778855
127	6	0	1.984921	7.055283	1.636186
128	6	0	7.090064	4.505439	2.556563
129	1	0	7.442968	4.355102	3.566168
130	6	0	3.441008	6.349533	-3.588425
131	6	0	8.907958	-4.159549	-0.233051
132	6	0	-7.154100	-3.072031	2.507189
133	1	0	-8.114016	-2.576654	2.538596
134	6	0	-1.702374	1.116172	1.808629
135	6	0	-1.211496	-7.061808	0.759252
136	6	0	-11.008770	1.103224	-0.331422
137	6	0	-1.980801	-7.266961	-2.755869
138	1	0	-1.860190	-7.549960	-3.791155
139	6	0	3.125181	6.512732	-5.037629
140	6	0	3.968001	7.253658	-5.873834
141	1	0	4.855500	7.715139	-5.450809
142	6	0	3.671420	7.406404	-7.224734
143	1	0	4.332517	7.988921	-7.858409
144	6	0	2.526479	6.821739	-7.760278
145	1	0	2.294781	6.941763	-8.813603
146	6	0	1.679221	6.083967	-6.937424
147	1	0	0.786441	5.622941	-7.347724
148	6	0	1.976951	5.931078	-5.586792
149	1	0	1.319132	5.354162	-4.942821
150	6	0	3.645917	6.184856	2.777406
151	6	0	8.445152	3.815053	-1.406665
152	6	0	8.748196	2.492494	-1.083032
153	1	0	8.004017	1.886067	-0.571493
154	6	0	9.998411	1.947843	-1.390345
155	6	0	10.960590	2.730994	-2.035968
156	1	0	11.929916	2.313014	-2.264767
157	6	0	10.651338	4.047063	-2.361542
158	1	0	11.398845	4.655956	-2.859669
159	6	0	9.410341	4.592323	-2.055537
160	1	0	9.179755	5.623582	-2.302781
161	6	0	-0.864184	-0.537213	-4.726359
162	6	0	0.068753	-0.054159	-5.651869
163	1	0	1.019240	0.330002	-5.292092
164	6	0	-0.213333	-0.061255	-7.013979
165	1	0	0.519485	0.320400	-7.717733
166	6	0	-1.433574	-0.553106	-7.471585
167	1	0	-1.653469	-0.561264	-8.534264
168	6	0	-2.369560	-1.032760	-6.559407
169	1	0	-3.321344	-1.421101	-6.907494
170	6	0	-2.090812	-1.023473	-5.195073
171	1	0	-2.825988	-1.397178	-4.486835
172	6	0	4.116026	-3.454513	-0.750581
173	6	0	3.873899	-4.797713	-1.050250
174	1	0	2.855995	-5.146068	-1.178976
175	6	0	4.936519	-5.682825	-1.188070
176	1	0	4.737391	-6.723840	-1.421236
177	6	0	6.249756	-5.259395	-1.027229
178	1	0	7.073170	-5.952188	-1.120150
179	6	0	6.500107	-3.916067	-0.726823
180	6	0	5.433778	-3.020461	-0.595166
181	1	0	5.628627	-1.979960	-0.344895
182	6	0	0.721771	-0.886710	3.475773
183	6	0	-1.188941	-7.602161	-1.704246
184	1	0	-0.286169	-8.194811	-1.717428
185	6	0	-1.833457	-5.601851	3.994758
186	1	0	-1.684387	-5.386901	5.042990
187	6	0	4.866721	5.571627	3.112712
188	6	0	11.443960	0.008270	-0.879347
189	6	0	2.343328	-2.686264	-2.967247
190	1	0	3.116524	-3.376495	-3.273469
191	6	0	-1.336871	0.510058	3.947584
192	1	0	-1.402203	0.345032	5.013283
193	6	0	-6.237614	-3.181163	3.502989
194	1	0	-6.301450	-2.774429	4.501107
195	6	0	0.823997	-1.251697	4.918155
196	6	0	1.045607	-2.007332	7.609487
197	1	0	1.133052	-2.299396	8.651007
198	6	0	2.855180	-2.169242	3.054783
199	1	0	3.140110	-2.324569	4.084665
200	6	0	3.494384	-2.604272	1.940830
201	1	0	4.391288	-3.202757	1.881759
202	6	0	-0.113440	8.170076	0.914522
203	6	0	-0.140279	9.400491	1.579623
204	1	0	0.799300	9.874172	1.849145
205	6	0	-1.351595	10.014693	1.884165
206	1	0	-1.355910	10.971560	2.396405
207	6	0	-2.553556	9.409613	1.524446
208	1	0	-3.497731	9.890830	1.757926
209	6	0	-2.539105	8.185002	0.861093
210	1	0	-3.470488	7.702438	0.579578
211	6	0	-1.326728	7.568484	0.561764
212	1	0	-1.315926	6.609159	0.050430
213	6	0	1.506484	7.397973	-0.790266
214	6	0	1.183015	7.510605	0.574099
215	6	0	5.554524	5.111521	-4.215192
216	1	0	5.413918	5.103390	-5.285980
217	6	0	1.344987	7.642711	-3.022720
218	1	0	1.062570	7.916915	-4.028442
219	6	0	6.585293	4.584679	-3.507185
220	1	0	7.452970	4.064071	-3.885392
221	6	0	-2.894083	1.757452	-1.784582
222	1	0	-3.651166	2.526619	-1.756720
223	6	0	-2.146272	1.282259	3.175545
224	1	0	-3.001308	1.867391	3.484335
225	6	0	0.716164	7.962236	-1.864343
226	1	0	-0.183762	8.545549	-1.735118
227	6	0	-3.792361	-3.727045	5.062645
228	6	0	-4.704953	-4.000722	6.087599
229	1	0	-5.540709	-4.665283	5.888697
230	6	0	-4.540860	-3.438491	7.350145
231	1	0	-5.255753	-3.663798	8.135092
232	6	0	-3.461271	-2.596713	7.609272
233	1	0	-3.337931	-2.157593	8.593977
234	6	0	-2.535274	-2.331935	6.603502
235	1	0	-1.676104	-1.694056	6.795598
236	6	0	-2.697339	-2.897022	5.341832
237	1	0	-1.954458	-2.705885	4.571150
238	6	0	1.659103	7.215502	3.038200
239	1	0	0.763329	7.681653	3.421395
240	6	0	2.692889	6.691628	3.743449
241	1	0	2.814213	6.657358	4.816197
242	6	0	5.174112	5.409730	4.565299
243	6	0	4.411867	4.545832	5.358547
244	1	0	3.595263	3.993924	4.900793
245	6	0	4.693128	4.390897	6.712624
246	1	0	4.094614	3.713821	7.313816
247	6	0	5.743513	5.097451	7.292753
248	1	0	5.964293	4.976756	8.348326
249	6	0	6.508815	5.960386	6.512314
250	1	0	7.325544	6.518374	6.958995
251	6	0	6.225175	6.116494	5.158710
252	1	0	6.815308	6.793401	4.547792
253	6	0	-12.253989	7.075024	-0.301231
254	1	0	-11.494999	7.381373	-1.036498
255	1	0	-11.871778	7.299879	0.703657
256	6	0	-13.563380	7.802054	-0.561316
257	1	0	-14.288911	7.433960	0.174908
258	6	0	-14.085264	7.499011	-1.964498
259	1	0	-15.044255	7.995249	-2.135683
260	1	0	-13.379021	7.862762	-2.719722
261	1	0	-14.219298	6.425488	-2.111099
262	6	0	-13.359097	9.302064	-0.352685
263	1	0	-14.295174	9.842607	-0.513071
264	1	0	-13.007656	9.527132	0.658589
265	1	0	-12.623162	9.694250	-1.063351
266	7	0	1.647776	1.535046	-0.132612
267	6	0	2.374309	1.739162	1.141728
268	1	0	3.091197	0.918295	1.243474
269	1	0	1.653070	1.649578	1.960007
270	7	0	2.978017	3.719288	-0.220897
271	6	0	3.069201	3.121835	1.130032
272	1	0	2.596636	3.821359	1.826401
273	1	0	4.127317	3.049709	1.398086
274	6	0	2.633628	1.480755	-1.234043
275	1	0	2.074849	1.386543	-2.171026
276	1	0	3.225984	0.567942	-1.112949
277	6	0	3.516490	2.758970	-1.208018
278	1	0	4.550770	2.541266	-0.920302
279	1	0	3.545139	3.252835	-2.184202
280	6	0	0.740077	2.683687	-0.356519
281	1	0	0.069539	2.736382	0.505683
282	1	0	0.130515	2.471912	-1.239347
283	6	0	1.556008	3.979455	-0.529298
284	1	0	1.504987	4.364386	-1.552086
285	1	0	1.205140	4.772673	0.138042
286	6	0	0.970281	-0.284927	5.919196
287	1	0	1.014010	0.764030	5.639879
288	6	0	1.079429	-0.660193	7.256089
289	1	0	1.201557	0.100583	8.020567
290	6	0	0.894662	-2.977402	6.620488
291	1	0	0.854393	-4.028016	6.890270
292	6	0	0.783547	-2.605611	5.284138
293	1	0	0.649540	-3.353070	4.505173
294	8	0	13.651870	-4.137310	0.484182
295	6	0	15.976008	-4.510316	0.870933
296	1	0	15.699060	-4.906598	1.855888
297	6	0	14.920265	-3.486250	0.487700
298	1	0	14.901503	-2.652908	1.202771
299	1	0	15.127771	-3.079832	-0.513227
300	6	0	17.334987	-3.819176	0.975868
301	1	0	17.323341	-3.009753	1.711714
302	1	0	18.106349	-4.534074	1.272420
303	1	0	17.628850	-3.395603	0.009132
304	6	0	16.009953	-5.660218	-0.133922
305	1	0	15.034406	-6.144060	-0.212410
306	1	0	16.290206	-5.289841	-1.126720
307	1	0	16.746427	-6.411313	0.163664
308	8	0	-4.143310	-2.012583	-2.751343
309	8	0	-0.868215	-3.163334	2.466835
310	7	0	-0.238160	-3.703797	1.579677
311	8	0	-6.994036	0.754224	0.788581
312	6	0	-3.557034	-2.183652	-1.707189
313	6	0	-3.933905	-1.688622	-0.360919
314	6	0	-2.972065	-2.093633	0.592447
315	6	0	-3.139498	-1.689590	1.911883
316	1	0	-2.425136	-1.919797	2.680685
317	6	0	-4.263274	-0.936703	2.256990
318	1	0	-4.415559	-0.632002	3.285744
319	6	0	-5.177509	-0.562132	1.285893
320	6	0	-5.029594	-0.914254	-0.057136
321	1	0	-5.730758	-0.607913	-0.825926
322	8	0	-0.053939	-5.005368	-4.211095
323	8	0	-6.533901	0.417715	2.868721
324	8	0	0.810690	-4.294668	1.759844
325	8	0	1.244813	-5.740376	-2.655574
326	7	0	-6.314438	0.258139	1.690598
327	7	0	0.301328	-5.081653	-3.049498
328	6	0	-2.295718	-2.951293	-1.490713
329	6	0	-1.590849	-3.615678	-2.470788
330	1	0	-1.904029	-3.580843	-3.508829
331	6	0	-0.467944	-4.323669	-2.059312
332	6	0	-0.055794	-4.343283	-0.740479
333	1	0	0.811358	-4.916740	-0.440371
334	6	0	-0.775036	-3.627476	0.217065
335	6	0	-1.933469	-2.914080	-0.125240

Table S2. Cartesian coordinates of *anti*-conformation of **G2•1Zn•G1•G2•G1•1Zn•G2**.

Number of imaginary frequencies = 0

Total energy (hartree) = –28817.175489

Center Number	Atomic Number	Atomic Type	Coordinates (Angstroms) X	Y	Z
1	30	0	11.892059	0.468547	-0.126978
2	30	0	3.495359	0.607951	-0.586249
3	30	0	18.940478	0.764081	0.207930
4	7	0	3.841274	2.241840	0.680621
5	7	0	3.961846	-0.967216	-1.891223
6	7	0	11.587259	-0.652536	-1.864220
7	7	0	4.096714	-0.637880	0.986259
8	7	0	11.394629	1.569579	1.579247
9	7	0	11.463154	2.195693	-1.243388
10	7	0	3.713584	1.906144	-2.202819
11	7	0	19.507994	-1.101369	-0.546414

12	7	0	19.251284	0.002382	2.130547
13	7	0	15.835368	-8.620073	0.906697
14	6	0	14.913253	-9.420775	0.308382
15	6	0	14.931259	-10.810200	0.406426
16	1	0	14.175579	-11.393588	-0.100764
17	6	0	15.930818	-11.424544	1.172772
18	6	0	16.881872	-10.616680	1.803886
19	1	0	17.685648	-11.001935	2.414751
20	6	0	16.799318	-9.230455	1.633936
21	7	0	11.473568	-1.271566	0.958271
22	7	0	19.109240	2.686691	1.018783
23	7	0	9.918640	8.564231	0.641112
24	1	0	9.010346	8.188990	0.374376
25	7	0	7.672554	10.029153	-0.101264
26	6	0	8.799109	10.691795	0.238373
27	6	0	8.910155	12.087529	0.220872
28	1	0	9.855310	12.521718	0.513693
29	6	0	7.799144	12.839509	-0.172471
30	6	0	6.622528	12.167139	-0.528889
31	1	0	5.739705	12.707699	-0.840564
32	6	0	6.601613	10.774862	-0.477129
33	6	0	3.728756	3.582996	0.328566
34	7	0	5.297959	8.732982	-0.738196
35	1	0	6.149536	8.310343	-0.375109
36	7	0	19.398622	1.584245	-1.658179
37	8	0	11.055616	10.559382	0.997776
38	7	0	13.687363	-7.450294	-0.431314
39	1	0	14.404110	-6.989826	0.117823
40	6	0	3.971590	-0.917010	-3.280977
41	6	0	11.535307	-0.155619	-3.162797
42	6	0	3.795026	1.560689	-3.547649
43	8	0	18.705937	-8.973018	3.047344
44	6	0	3.608887	4.082285	-0.983189
45	8	0	7.767395	14.211056	-0.237041
46	6	0	11.137390	1.076682	2.853063
47	8	0	13.012538	-9.566654	-1.120753
48	6	0	11.642100	-2.949150	-0.882736
49	7	0	17.871621	-7.081054	1.982675
50	1	0	17.124550	-6.793381	1.360296
51	6	0	11.481120	1.204463	-3.532460
52	6	0	4.077666	-2.307736	-1.538033
53	6	0	11.241585	-1.363996	2.327049
54	6	0	3.817243	2.769954	-4.352236
55	1	0	3.919692	2.800545	-5.424601
56	6	0	19.716900	-1.423237	-1.881294
57	6	0	19.294128	-1.344087	2.467290
58	8	0	4.354102	10.789917	-1.271632
59	6	0	19.122120	0.709869	3.319077
60	6	0	11.472015	2.287781	-2.629898
61	6	0	11.529559	3.497828	-0.756444
62	6	0	3.848597	0.099009	-5.558832
63	6	0	4.934873	-0.454797	-6.264026
64	1	0	5.816596	-0.784384	-5.725304
65	6	0	4.889449	-0.588892	-7.655892
66	1	0	5.739200	-1.015895	-8.178906
67	6	0	3.759964	-0.173095	-8.369931
68	1	0	3.726814	-0.277799	-9.449750
69	6	0	2.672875	0.378064	-7.681695
70	1	0	1.789887	0.699661	-8.225360
71	6	0	2.717337	0.512607	-6.290557
72	1	0	1.869750	0.935704	-5.760865
73	6	0	4.185182	-0.277720	2.326452
74	6	0	11.633038	-2.040303	-1.964323
75	6	0	4.133196	-2.028923	0.943593
76	6	0	19.135114	-1.483570	3.904571
77	1	0	19.102979	-2.417886	4.441200
78	6	0	4.134997	-4.311991	-0.062386
79	6	0	3.042980	-5.087404	-0.500135
80	1	0	2.189398	-4.594063	-0.954286
81	6	0	3.045507	-6.478129	-0.352635
82	1	0	2.192778	-7.056867	-0.694211
83	6	0	4.142779	-7.120279	0.233267
84	1	0	4.147508	-8.199769	0.345382
85	6	0	5.234760	-6.361799	0.670566
86	1	0	6.093266	-6.851888	1.118770
87	6	0	5.232268	-4.969995	0.526710
88	1	0	6.085106	-4.391149	0.866486
89	6	0	11.531363	-2.574413	0.473736
90	6	0	3.876807	0.252384	-4.066207
91	6	0	3.661908	3.293823	-2.150133
92	6	0	11.699893	5.310433	0.967108
93	6	0	12.838141	5.697966	1.700962
94	1	0	13.571628	4.949810	1.981541
95	6	0	13.023479	7.035698	2.053667
96	1	0	13.909448	7.328402	2.608283
97	6	0	12.085949	8.011869	1.709885
98	1	0	12.234968	9.046099	1.977892
99	6	0	10.935442	7.638679	0.988579
100	6	0	10.762479	6.292921	0.610434
101	1	0	9.888431	6.014202	0.034328
102	6	0	3.727741	3.830285	-3.496975
103	1	0	3.739605	4.879003	-3.746409
104	6	0	10.021890	9.926913	0.661502
105	6	0	3.406311	5.560198	-1.166533
106	6	0	4.420941	6.484584	-0.879077
107	1	0	5.384775	6.132975	-0.533399
108	6	0	4.219765	7.868190	-1.050004
109	6	0	2.975615	8.331006	-1.522430
110	1	0	2.817770	9.389562	-1.656585
111	6	0	1.969502	7.406756	-1.814330
112	1	0	1.013453	7.769087	-2.179042
113	6	0	2.170263	6.035700	-1.643837
114	1	0	1.378020	5.331175	-1.873743
115	6	0	4.175311	1.036459	2.837213
116	6	0	19.573445	0.878371	-2.841399
117	6	0	19.469707	-2.416929	1.568526
118	6	0	4.013605	2.201892	2.059038
119	6	0	11.551689	3.684691	-3.018791
120	1	0	11.580617	4.046763	-4.033589
121	6	0	4.200411	-2.549020	2.296818
122	1	0	4.216018	-3.593013	2.563221
123	6	0	11.525745	3.864182	0.603776
124	6	0	19.601752	-2.285948	0.171514
125	6	0	11.550875	-1.264755	-4.099204
126	1	0	11.501065	-1.175322	-5.172190
127	6	0	19.212872	3.878310	0.312112
128	6	0	19.032514	-0.224946	4.426124
129	1	0	18.900148	0.045465	5.461279
130	6	0	19.715029	-0.517902	-2.959579
131	6	0	13.793085	-8.814252	-0.488339
132	6	0	3.804826	4.400989	1.525008
133	1	0	3.732072	5.476390	1.550213
134	6	0	11.381632	2.954523	1.673841
135	6	0	4.115136	-2.819921	-0.224542
136	6	0	5.319245	10.095374	-0.865219
137	6	0	4.090287	-2.264744	-3.808441
138	1	0	4.120896	-2.526383	-4.853235
139	6	0	19.919718	-1.076876	-4.338830
140	6	0	21.170952	-1.589633	-4.730071
141	1	0	21.994212	-1.572467	-4.023124
142	6	0	21.360955	-2.108071	-6.015584
143	1	0	22.333992	-2.495855	-6.300812
144	6	0	20.303755	-2.121848	-6.933049
145	1	0	20.451366	-2.524191	-7.930254
146	6	0	19.055402	-1.612610	-6.557078
147	1	0	18.229357	-1.621763	-7.261503
148	6	0	18.865793	-1.094490	-5.271297
149	1	0	17.895453	-0.703431	-4.981531
150	6	0	19.114714	3.020093	2.367539
151	6	0	19.595649	-3.791363	2.154452
152	6	0	18.679531	-4.807699	1.840620
153	1	0	17.854136	-4.588611	1.168659
154	6	0	18.818349	-6.102205	2.374574
155	6	0	19.882392	-6.383701	3.251893
156	1	0	19.987749	-7.375978	3.661861
157	6	0	20.791475	-5.370085	3.563787
158	1	0	21.617620	-5.591959	4.231770
159	6	0	20.662372	-4.089211	3.024941
160	1	0	21.386034	-3.317290	3.261089
161	6	0	11.445475	1.529044	-4.998061
162	6	0	12.544649	1.237261	-5.828564
163	1	0	13.427982	0.776500	-5.397520
164	6	0	12.512214	1.543079	-7.193032
165	1	0	13.371353	1.315701	-7.816596
166	6	0	11.378822	2.145695	-7.751337
167	1	0	11.352664	2.382831	-8.810239
168	6	0	10.280590	2.441781	-6.935686
169	1	0	9.396458	2.906705	-7.360520
170	6	0	10.311193	2.138647	-5.569824
171	1	0	9.453370	2.370107	-4.945723
172	6	0	11.658908	-4.418108	-1.196777
173	6	0	10.614231	-4.984243	-1.952802
174	1	0	9.802912	-4.362589	-2.308786
175	6	0	10.607474	-6.350706	-2.232384
176	1	0	9.795898	-6.771244	-2.816907
177	6	0	11.616126	-7.191901	-1.761330
178	1	0	11.603812	-8.249970	-1.969129
179	6	0	12.657226	-6.646691	-0.985777
180	6	0	12.677683	-5.263149	-0.721113
181	1	0	13.486556	-4.841000	-0.130567
182	6	0	11.069922	-0.283748	3.214637
183	6	0	4.169733	-3.114852	-2.741030
184	1	0	4.292774	-4.185395	-2.765770
185	6	0	4.243092	-1.477626	3.142272
186	1	0	4.296705	-1.496561	4.218456
187	6	0	19.074788	2.112616	3.445386
188	6	0	17.872135	-8.411372	2.294067
189	6	0	11.636354	-2.415816	-3.367540
190	1	0	11.683792	-3.424188	-3.744363
191	6	0	10.938237	2.188275	3.764820
192	1	0	10.693307	2.098861	4.810934
193	6	0	4.004983	3.557285	2.581783
194	1	0	4.137454	3.828561	3.616549
195	6	0	10.759745	-0.607143	4.647565
196	6	0	10.157451	-1.208696	7.338390
197	1	0	9.926991	-1.442533	8.373049
198	6	0	11.183403	-2.762555	2.709790
199	1	0	11.011958	-3.127377	3.709211
200	6	0	11.355064	-3.503032	1.575787
201	1	0	11.327945	-4.576506	1.487049
202	6	0	19.349175	5.399777	-1.648653
203	6	0	20.511890	5.892051	-2.272164
204	1	0	21.386588	5.253381	-2.340175
205	6	0	20.551032	7.191558	-2.788514
206	1	0	21.458277	7.555380	-3.260858
207	6	0	19.428818	8.022752	-2.691014
208	1	0	19.459473	9.030929	-3.091926
209	6	0	18.267493	7.546662	-2.071466
210	1	0	17.391652	8.183237	-1.993199
211	6	0	18.229063	6.247516	-1.554008
212	1	0	17.325325	5.881134	-1.077425
213	6	0	19.399382	2.932811	-1.992791
214	6	0	19.305129	4.008211	-1.088280
215	6	0	19.940694	-2.852906	-2.001405
216	1	0	20.131416	-3.377660	-2.923634
217	6	0	19.656415	1.811811	-3.950538
218	1	0	19.800826	1.538377	-4.983410
219	6	0	19.884781	-3.379462	-0.742692
220	1	0	20.038777	-4.407232	-0.456470
221	6	0	11.602995	4.424664	-1.871457
222	1	0	11.686877	5.496168	-1.787340
223	6	0	11.106845	3.339603	3.046236
224	1	0	11.019648	4.354191	3.401150
225	6	0	19.539017	3.069956	-3.432054
226	1	0	19.557897	4.004272	-3.969330
227	6	0	4.369269	1.201439	4.315565
228	6	0	3.369167	1.771370	5.126805
229	1	0	2.433182	2.084704	4.675444
230	6	0	3.566406	1.928105	6.502977
231	1	0	2.781701	2.366239	7.111900
232	6	0	4.767335	1.518163	7.094038
233	1	0	4.920567	1.642961	8.161280
234	6	0	5.768310	0.945157	6.300502
235	1	0	6.704877	0.624370	6.746270
236	6	0	5.570388	0.786109	4.925054
237	1	0	6.346272	0.334408	4.315349
238	6	0	19.282747	4.987185	1.246858
239	1	0	19.395984	6.023762	0.973089
240	6	0	19.223816	4.461517	2.505899
241	1	0	19.282859	4.993386	3.441764
242	6	0	19.007767	2.685594	4.829625
243	6	0	17.893361	3.439876	5.245042
244	1	0	17.073353	3.598511	4.551865
245	6	0	17.830653	3.976635	6.535201
246	1	0	16.960526	4.551109	6.837740
247	6	0	18.883346	3.771199	7.434612
248	1	0	18.835910	4.188631	8.435409
249	6	0	19.998678	3.025912	7.034239
250	1	0	20.823133	2.868056	7.722620
251	6	0	20.060361	2.488016	5.744552
252	1	0	20.930882	1.919727	5.433754
253	6	0	8.963535	14.984336	0.123281
254	1	0	9.796643	14.684074	-0.526772
255	1	0	9.229000	14.764442	1.165132
256	6	0	8.629876	16.465583	-0.061869
257	1	0	7.748296	16.675672	0.560346
258	6	0	8.282123	16.788073	-1.526159
259	1	0	7.997454	17.841075	-1.632216
260	1	0	9.145628	16.606044	-2.180118
261	1	0	7.451563	16.168498	-1.875544
262	6	0	9.805555	17.324078	0.445877
263	1	0	9.569635	18.389310	0.349688
264	1	0	10.029412	17.123112	1.500510
265	1	0	10.715315	17.131291	-0.137555
266	7	0	14.118322	0.542407	-0.044102
267	6	0	14.560833	0.653579	1.390028
268	1	0	14.159558	-0.211251	1.924417
269	1	0	14.094852	1.547317	1.809660
270	7	0	16.703978	0.622543	0.085934
271	6	0	16.113960	0.716058	1.466049
272	1	0	16.462769	1.652224	1.907371
273	1	0	16.524907	-0.103635	2.058619
274	6	0	14.706423	-0.710515	-0.635227
275	1	0	14.359870	-0.781037	-1.668368
276	1	0	14.290055	-1.558469	-0.086463
277	6	0	16.261347	-0.667400	-0.545344
278	1	0	16.657771	-1.484627	0.061620
279	1	0	16.728187	-0.732389	-1.530837
280	6	0	14.639772	1.730389	-0.808550
281	1	0	14.192692	2.626201	-0.372878
282	1	0	14.278707	1.647583	-1.836170
283	6	0	16.193482	1.769182	-0.741239
284	1	0	16.646849	1.688327	-1.731313
285	1	0	16.558415	2.691311	-0.283848
286	6	0	11.670660	-0.298296	5.675190
287	1	0	12.617043	0.169813	5.423193
288	6	0	11.372248	-0.596249	7.009406
289	1	0	12.089418	-0.355717	7.788093
290	6	0	9.243833	-1.522330	6.324299
291	1	0	8.300780	-2.001072	6.570257
292	6	0	9.540019	-1.224881	4.989078
293	1	0	8.828785	-1.462301	4.204438
294	8	0	15.896322	-12.792982	1.245495
295	6	0	16.622667	-15.005955	1.907541
296	1	0	15.589151	-15.162900	2.247548
297	6	0	16.911300	-13.509286	2.033172
298	1	0	16.848017	-13.178208	3.077352
299	1	0	17.908015	-13.265603	1.641214
300	6	0	17.570216	-15.790175	2.837327
301	1	0	17.463809	-15.477810	3.883170
302	1	0	17.355003	-16.862669	2.783788
303	1	0	18.618402	-15.646741	2.544305
304	6	0	16.735100	-15.483321	0.448569
305	1	0	16.062558	-14.916555	-0.201279
306	1	0	17.760603	-15.357470	0.075933
307	1	0	16.478152	-16.545704	0.369937
308	6	0	0.865236	-1.003248	-0.740747
309	1	0	1.404867	-1.668316	-0.063130
310	1	0	1.206178	-1.214814	-1.756374
311	6	0	-0.675458	-1.163240	-0.600888
312	1	0	-1.141202	-1.460315	-1.542593
313	1	0	-0.939854	-1.910436	0.150395
314	6	0	0.844301	0.714691	1.000852
315	1	0	1.171227	1.731499	1.228237
316	1	0	1.383464	0.034478	1.663730
317	6	0	-0.698200	0.556361	1.139276
318	1	0	-0.965262	-0.205493	1.874961
319	1	0	-1.178401	1.490339	1.438519
320	6	0	0.567538	1.350049	-1.345935
321	1	0	0.912101	1.129296	-2.358738
322	1	0	0.893647	2.363360	-1.099796
323	6	0	-0.976750	1.188366	-1.208581
324	1	0	-1.455573	2.118861	-0.895093
325	1	0	-1.438253	0.879658	-2.149496
326	7	0	-1.296038	0.140293	-0.177497
327	7	0	1.271946	0.406774	-0.408921
328	30	0	-11.904663	-0.520285	0.184474
329	30	0	-3.500089	-0.152468	0.070395
330	30	0	-18.930278	-1.222872	0.303773
331	7	0	-3.857906	-1.723414	-1.273638
332	7	0	-3.941380	1.303090	1.512204
333	7	0	-11.531947	0.500502	1.969925
334	7	0	-4.290505	1.150306	-1.363109
335	7	0	-11.476562	-1.474936	-1.625654
336	7	0	-11.298572	-2.288223	1.143878
337	7	0	-3.512117	-1.565562	1.607699
338	7	0	-19.539792	0.542222	1.245122
339	7	0	-19.430567	-0.338297	-1.523765
340	7	0	-16.416173	8.367594	0.082905
341	6	0	-15.490963	9.180330	0.659841
342	6	0	-15.588347	10.569944	0.656458
343	1	0	-14.824851	11.162317	1.141003
344	6	0	-16.677407	11.173388	0.013411
345	6	0	-17.634429	10.353938	-0.593196
346	1	0	-18.504644	10.730395	-1.111501
347	6	0	-17.466793	8.966718	-0.523682
348	7	0	-11.665548	1.309616	-0.802827
349	7	0	-19.053987	-3.084655	-0.644266
350	7	0	-9.530291	-8.408276	-1.285448
351	1	0	-8.629222	-7.994364	-1.053839
352	7	0	-7.153959	-9.773617	-0.802020
353	6	0	-8.260183	-10.482111	-1.114858
354	6	0	-8.286429	-11.879945	-1.193334
355	1	0	-9.222297	-12.352668	-1.454614
356	6	0	-7.107816	-12.584436	-0.930481
357	6	0	-5.951207	-11.864205	-0.603199
358	1	0	-5.018160	-12.367270	-0.390866
359	6	0	-6.016868	-10.473304	-0.553237

360	6	0	-3.652130	-3.076494	-1.022998
361	7	0	-4.822954	-8.374739	-0.228435
362	1	0	-5.721646	-7.984457	-0.503405
363	7	0	-19.195379	-2.207816	2.126921
364	8	0	-10.568795	-10.443372	-1.710197
365	7	0	-14.108636	7.240580	1.170913
366	1	0	-14.841322	6.774731	0.647999
367	6	0	-3.852940	1.161507	2.892745
368	6	0	-11.357437	-0.076839	3.223713
369	6	0	-3.521503	-1.313686	2.976207
370	8	0	-19.469114	8.691726	-1.793960
371	6	0	-3.402591	-3.651410	0.238315
372	8	0	-6.990046	-13.952202	-0.969704
373	6	0	-11.339555	-0.884120	-2.876385
374	8	0	-13.492230	9.345175	1.945612
375	6	0	-11.791761	2.848783	1.158499
376	7	0	-18.449117	6.785651	-0.936323
377	1	0	-17.638277	6.501645	-0.397703
378	6	0	-11.200027	-1.452419	3.492237
379	6	0	-4.149311	2.655078	1.260104
380	6	0	-11.538933	1.505899	-2.174390
381	6	0	-3.427686	-2.572031	3.695420
382	1	0	-3.455172	-2.677969	4.767520
383	6	0	-19.659257	0.749308	2.613266
384	6	0	-19.577849	1.024251	-1.747140
385	8	0	-3.722361	-10.397982	0.089854
386	6	0	-19.350689	-0.943564	-2.771589
387	6	0	-11.197478	-2.471372	2.517757
388	6	0	-11.324854	-3.557072	0.572915
389	6	0	-3.521524	0.008242	5.082607
390	6	0	-4.586431	0.451494	5.891039
391	1	0	-5.518562	0.763566	5.432763
392	6	0	-4.455547	0.497418	7.283185
393	1	0	-5.290265	0.839568	7.886633
394	6	0	-3.260300	0.102273	7.894573
395	1	0	-3.160947	0.138332	8.974817
396	6	0	-2.193548	-0.339256	7.103174
397	1	0	-1.260210	-0.643749	7.566634
398	6	0	-2.323469	-0.385860	5.711643
399	1	0	-1.491415	-0.724288	5.102445
400	6	0	-4.446278	0.874440	-2.716700
401	6	0	-11.650364	1.872927	2.170344
402	6	0	-4.392190	2.531296	-1.223083
403	6	0	-19.538845	1.282365	-3.176244
404	1	0	-19.603829	2.254820	-3.636761
405	6	0	-4.441241	4.740808	-0.066461
406	6	0	-3.369080	5.558206	0.342390
407	1	0	-2.452280	5.095059	0.693457
408	6	0	-3.471433	6.952124	0.294826
409	1	0	-2.632359	7.564526	0.610013
410	6	0	-4.649925	7.555102	-0.160078
411	1	0	-4.731418	8.636806	-0.194477
412	6	0	-5.722810	6.754445	-0.568410
413	1	0	-6.643121	7.213094	-0.915935
414	6	0	-5.620833	5.359774	-0.524533
415	1	0	-6.459319	4.747731	-0.840987
416	6	0	-11.760581	2.571753	-0.225346
417	6	0	-3.641285	-0.048921	3.587726
418	6	0	-3.399997	-2.942925	1.457135
419	6	0	-11.518230	-5.259744	-1.257225
420	6	0	-12.682610	-5.669287	-1.936126
421	1	0	-13.477027	-4.952146	-2.112497
422	6	0	-12.814744	-6.989868	-2.368416
423	1	0	-13.720317	-7.300810	-2.879757
424	6	0	-11.799087	-7.925315	-2.159892
425	1	0	-11.906523	-8.946879	-2.489309
426	6	0	-10.622505	-7.528374	-1.495559
427	6	0	-10.501368	-6.202308	-1.036379
428	1	0	-9.605035	-5.907383	-0.504285
429	6	0	-3.344492	-3.568161	2.765525
430	1	0	-3.288273	-4.629992	2.943016
431	6	0	-9.553288	-9.770147	-1.398404
432	6	0	-3.101977	-5.122208	0.304457
433	6	0	-4.075801	-6.089283	0.016794
434	1	0	-5.080526	-5.778939	-0.240774
435	6	0	-3.779942	-7.465174	0.075045
436	6	0	-2.480809	-7.877155	0.432731
437	1	0	-2.250815	-8.929939	0.480285
438	6	0	-1.514965	-6.911122	0.725818
439	1	0	-0.515857	-7.232961	1.003121
440	6	0	-1.809749	-5.547763	0.667280
441	1	0	-1.047270	-4.811069	0.896924
442	6	0	-4.394748	-0.399941	-3.317299
443	6	0	-19.316580	-1.605760	3.372603
444	6	0	-19.743200	2.012878	-0.754930
445	6	0	-4.119832	-1.602687	-2.633021
446	6	0	-11.166300	-3.893071	2.812491
447	1	0	-11.097510	-4.321922	3.798964
448	6	0	-4.580846	3.133922	-2.529779
449	1	0	-4.668712	4.190568	-2.721934
450	6	0	-11.402132	-3.832448	-0.806510
451	6	0	-19.755064	1.769682	0.633058
452	6	0	-11.368208	0.964848	4.234661
453	1	0	-11.235683	0.807858	5.292765
454	6	0	-19.039554	-4.330577	-0.029736
455	6	0	-19.401540	0.076384	-3.803369
456	6	0	-19.523598	-0.233346	3.612571
457	6	0	-14.280497	8.590125	1.326028
458	6	0	-3.764796	-3.816990	-2.265979
459	1	0	-3.637030	-4.882420	-2.370567
460	6	0	-11.391519	-2.847187	-1.817712
461	6	0	-4.319451	3.246174	-0.009355
462	6	0	-4.753439	-9.741127	-0.200690
463	6	0	-4.001941	2.463413	3.518137
464	1	0	-3.973202	2.654150	4.578230
465	6	0	-19.649799	0.205863	5.043554
466	6	0	-20.891419	0.616545	5.564520
467	1	0	-21.764501	0.608015	4.919957
468	6	0	-21.008136	1.022900	6.898184
469	1	0	-21.974619	1.332718	7.283479
470	6	0	-19.885929	1.024620	7.734991
471	1	0	-19.976739	1.339945	8.769596
472	6	0	-18.646206	0.616127	7.229755
473	1	0	-17.770206	0.616822	7.871060
474	6	0	-18.529910	0.210125	5.895876
475	1	0	-17.566306	-0.102858	5.506056
476	6	0	-19.139765	-3.314308	-2.011699
477	6	0	-19.995431	3.416254	-1.219022
478	6	0	-19.114396	4.462253	-0.902537
479	1	0	-18.223590	4.247012	-0.318616
480	6	0	-19.370386	5.780657	-1.322749
481	6	0	-20.519801	6.057898	-2.086385
482	1	0	-20.714984	7.068614	-2.409047
483	6	0	-21.393936	5.014827	-2.400938
484	1	0	-22.285196	5.232426	-2.980809
485	6	0	-21.147385	3.708901	-1.975054
486	1	0	-21.843525	2.912144	-2.211527
487	6	0	-11.037159	-1.869867	4.925435
488	6	0	-12.088038	-1.706290	5.848383
489	1	0	-13.027145	-1.277441	5.512904
490	6	0	-11.936652	-2.098196	7.182513
491	1	0	-12.759819	-1.969331	7.878538
492	6	0	-10.730565	-2.660120	7.617289
493	1	0	-10.612133	-2.964003	8.652579
494	6	0	-9.679290	-2.828986	6.708788
495	1	0	-8.739555	-3.261480	7.037694
496	6	0	-9.828783	-2.439520	5.372965
497	1	0	-9.006706	-2.572239	4.676175
498	6	0	-11.866515	4.290076	1.575454
499	6	0	-10.800909	4.866141	2.293808
500	1	0	-9.932874	4.271520	2.547259
501	6	0	-10.847524	6.209073	2.667457
502	1	0	-10.018723	6.637376	3.221360
503	6	0	-11.932682	7.018139	2.328847
504	1	0	-11.962203	8.059162	2.608486
505	6	0	-12.997557	6.464481	1.592543
506	6	0	-12.962432	5.102638	1.233429
507	1	0	-13.788810	4.673158	0.673156
508	6	0	-11.372781	0.498867	-3.145244
509	6	0	-4.196647	3.376001	2.519282
510	1	0	-4.371747	4.434192	2.625575
511	6	0	-4.623387	2.119539	-3.442637
512	1	0	-4.749540	2.205518	-4.509456
513	6	0	-19.231128	-2.326790	-3.013388
514	6	0	-18.546464	8.131346	-1.151423
515	6	0	-11.572694	2.154163	3.593292
516	1	0	-11.650722	3.130546	4.042340
517	6	0	-11.145112	-1.919004	-3.875318
518	1	0	-10.982001	-1.746087	-4.926905
519	6	0	-4.075344	-2.918085	-3.247863
520	1	0	-4.260126	-3.127772	-4.288891
521	6	0	-11.184806	0.934427	-4.569714
522	6	0	-10.811786	1.747955	-7.246392
523	1	0	-10.669604	2.063153	-8.275346
524	6	0	-11.587149	2.927624	-2.461328
525	1	0	-11.509515	3.367251	-3.442164
526	6	0	-11.717315	3.579979	-1.269138
527	1	0	-11.744126	4.644945	-1.107631
528	6	0	-18.944422	-6.002068	1.807298
529	6	0	-20.028979	-6.609883	2.468517
530	1	0	-20.930055	-6.032453	2.648163
531	6	0	-19.958276	-7.944928	2.880317
532	1	0	-20.806680	-8.397996	3.383811
533	6	0	-18.802329	-8.696697	2.638692
534	1	0	-18.747735	-9.732470	2.958518
535	6	0	-17.717936	-8.105288	1.980266
536	1	0	-16.816589	-8.679774	1.790557
537	6	0	-17.789427	-6.770579	1.567380
538	1	0	-16.945012	-6.314721	1.060181
539	6	0	-19.099588	-3.575794	2.351084
540	6	0	-19.018320	-4.571720	1.358642
541	6	0	-19.949325	2.149924	2.863610
542	1	0	-20.093293	2.591500	3.836495
543	6	0	-19.265165	-2.624440	4.406093
544	1	0	-19.344710	-2.439657	5.465320
545	6	0	-20.022719	2.772262	1.650297
546	1	0	-20.253879	3.807699	1.459411
547	6	0	-11.260228	-4.557875	1.622622
548	1	0	-11.287574	-5.624694	1.469725
549	6	0	-11.196120	-3.123353	-3.229391
550	1	0	-11.078072	-4.105014	-3.659936
551	6	0	-19.121606	-3.830306	3.780971
552	1	0	-19.050383	-4.802457	4.241228
553	6	0	-4.676097	-0.479218	-4.788606
554	6	0	-3.702070	-0.934291	-5.698053
555	1	0	-2.722210	-1.219502	-5.328535
556	6	0	-3.981652	-1.013331	-7.066588
557	1	0	-3.216243	-1.363324	-7.752286
558	6	0	-5.240332	-0.639050	-7.551636
559	1	0	-5.457150	-0.703517	-8.613180
560	6	0	-6.216620	-0.179897	-6.659440
561	1	0	-7.197357	0.111851	-7.022506
562	6	0	-5.936596	-0.098546	-5.291510
563	1	0	-6.694121	0.264757	-4.604494
564	6	0	-19.116951	-5.367405	-1.043312
565	6	0	-19.178917	-4.744994	-2.257315
566	6	0	-19.233772	-2.787644	-4.440445
567	6	0	-18.111836	-3.437171	-4.990702
568	1	0	-17.235494	-3.596637	-4.370470
569	6	0	-18.113642	-3.869307	-6.321047
570	1	0	-17.236838	-4.363686	-6.727832
571	6	0	-19.239522	-3.662210	-7.126532
572	1	0	-19.242050	-3.998504	-8.158518
573	6	0	-20.363076	-3.020804	-6.591801
574	1	0	-21.243556	-2.862968	-7.206907
575	6	0	-20.360353	-2.587446	-5.261911
576	1	0	-21.236637	-2.100030	-4.847307
577	6	0	-8.160206	-14.774042	-1.307234
578	1	0	-8.962495	-14.573781	-0.584037
579	1	0	-8.510055	-14.500001	-2.310618
580	6	0	-7.726784	-16.239976	-1.253499
581	1	0	-6.879472	-16.349159	-1.945311
582	6	0	-7.259828	-16.640064	0.157517
583	1	0	-6.906065	-17.677294	0.167238
584	1	0	-8.085366	-16.559954	0.877590
585	1	0	-6.445610	-15.993847	0.496964
586	6	0	-8.881476	-17.135230	-1.745685
587	1	0	-8.576231	-18.187108	-1.743741
588	1	0	-9.189546	-16.876367	-2.765896
589	1	0	-9.758473	-17.042598	-1.091835
590	7	0	-14.122845	-0.723277	0.247765
591	6	0	-14.665992	-0.665268	-1.154530
592	1	0	-14.355506	0.289425	-1.586400
593	1	0	-14.183740	-1.460357	-1.726704
594	7	0	-16.702164	-0.953840	0.282455
595	6	0	-16.214685	-0.819298	-1.133846
596	1	0	-16.542698	-1.707626	-1.678145
597	1	0	-16.715005	0.044128	-1.576921
598	6	0	-14.732507	0.395624	1.048672
599	1	0	-14.312475	0.347115	2.055529
600	1	0	-14.407054	1.336484	0.598736
601	6	0	-16.284914	0.263300	1.058943
602	1	0	-16.771445	1.128511	0.603008
603	1	0	-16.678146	0.160700	2.072991
604	6	0	-14.517966	-2.036737	0.868987
605	1	0	-14.056743	-2.832120	0.280065
606	1	0	-14.084138	-2.073945	1.870717
607	6	0	-16.067589	-2.166920	0.903166
608	1	0	-16.447350	-2.250777	1.923485
609	1	0	-16.415637	-3.039762	0.346809
610	6	0	-12.147869	0.638441	-5.552637
611	1	0	-13.045628	0.097284	-5.270805
612	6	0	-11.963128	1.041493	-6.879807
613	1	0	-12.719349	0.809062	-7.623211
614	6	0	-9.847258	2.049733	-6.276855
615	1	0	-8.952961	2.600812	-6.551804
616	6	0	-10.029742	1.647305	-4.948801
617	1	0	-9.278832	1.876158	-4.199363
618	8	0	-16.719006	12.543479	0.029031
619	6	0	-17.606026	14.748939	-0.431845
620	1	0	-16.613360	14.981528	-0.842841
621	6	0	-17.829914	13.248831	-0.628295
622	1	0	-17.837246	12.983922	-1.693044
623	1	0	-18.777429	12.928653	-0.174253
624	6	0	-18.665083	15.533937	-1.231567
625	1	0	-18.630614	15.289987	-2.300207
626	1	0	-18.499357	16.611518	-1.127740
627	1	0	-19.676958	15.316750	-0.865429
628	6	0	-17.620138	15.132842	1.058642
629	1	0	-16.868692	14.566286	1.615586
630	1	0	-18.603598	14.929708	1.503504
631	1	0	-17.410353	16.201171	1.183277
632	8	0	7.688195	2.727657	-3.251589
633	8	0	7.621011	-0.788019	2.063633
634	7	0	7.748823	-1.581956	1.090256
635	8	0	7.493109	6.594867	0.396151
636	6	0	7.667942	2.113732	-2.172963
637	6	0	7.629664	2.677982	-0.808839
638	6	0	7.661250	1.628924	0.153009
639	6	0	7.660850	1.967244	1.510668
640	1	0	7.706220	1.213607	2.274727
641	6	0	7.602468	3.316988	1.880720
642	1	0	7.593669	3.595788	2.925382
643	6	0	7.553523	4.315149	0.904051
644	6	0	7.577878	4.013101	-0.464985
645	1	0	7.555499	4.786318	-1.221065
646	8	0	7.615830	-2.303536	-4.855847
647	8	0	7.421332	5.984799	2.540908
648	8	0	7.900989	-2.826902	1.241560
649	8	0	7.713868	-3.886403	-3.287830
650	7	0	7.482971	5.703615	1.317105
651	7	0	7.677138	-2.675113	-3.649209
652	6	0	7.691459	0.640271	-1.984951
653	6	0	7.698670	-0.297317	-2.996858
654	1	0	7.701625	-0.000426	-4.037017
655	6	0	7.701544	-1.642649	-2.617610
656	6	0	7.720783	-2.020616	-1.282835
657	1	0	7.731241	-3.066023	-1.012141
658	6	0	7.721847	-1.045669	-0.272384
659	6	0	7.691814	0.327128	-0.593865
660	8	0	-7.356838	-2.700938	2.849362
661	8	0	-7.880375	1.153510	-2.198694
662	7	0	-7.979070	1.872812	-1.165761
663	8	0	-7.216147	-6.301774	-1.064608
664	6	0	-7.450352	-2.017554	1.817335
665	6	0	-7.484222	-2.487797	0.418008
666	6	0	-7.644749	-1.381587	-0.463681
667	6	0	-7.727074	-1.629711	-1.838265
668	1	0	-7.870907	-0.831795	-2.542922
669	6	0	-7.621288	-2.946298	-2.304709
670	1	0	-7.674462	-3.155130	-3.364268
671	6	0	-7.444440	-4.001565	-1.405953
672	6	0	-7.384420	-3.791985	-0.020932
673	1	0	-7.263008	-4.610318	0.675912
674	8	0	-7.450317	2.208351	4.793006
675	8	0	-7.340762	-5.548703	-3.160951
676	8	0	-8.211457	3.113418	-1.218708
677	8	0	-7.750480	3.882261	3.350201
678	7	0	-7.326157	-5.352972	-1.919143
679	7	0	-7.618873	2.654337	3.622257
680	6	0	-7.567914	-0.538909	1.734807
681	6	0	-7.552045	0.327899	2.807875
682	1	0	-7.460765	-0.036319	3.822313
683	6	0	-7.659234	1.692549	2.524894
684	6	0	-7.799027	2.155603	1.224518
685	1	0	-7.888958	3.213954	1.029261
686	6	0	-7.820527	1.250958	0.150836
687	6	0	-7.690280	-0.135649	0.372544
688	1	0	-19.332890	-0.105443	-4.863742
689	1	0	-19.276728	-5.206841	-3.226513
690	1	0	-19.152698	-6.426746	-0.846375

Table S3. Cartesian coordinates of *syn*-conformation of **G2•1Zn•G1•G2•G1•1Zn•G2**.

Number of imaginary frequencies = 0

Total energy (hartree) = –28817.175020

Center Number	Atomic Number	Atomic Type	Coordinates (Angstroms) X	Y	Z
1	6	0	0.749835	-1.833418	-0.606549
2	1	0	1.213164	-2.523610	0.102048
3	1	0	1.078535	-2.115620	-1.608967
4	6	0	-0.801450	-1.830681	-0.482674
5	1	0	-1.285507	-2.106746	-1.421670
6	1	0	-1.149434	-2.522988	0.287051
7	6	0	0.890606	-0.060472	1.072679
8	1	0	1.311415	0.928007	1.268326
9	1	0	1.358686	-0.764795	1.763886
10	6	0	-0.660976	-0.063708	1.202362
11	1	0	-1.006098	-0.774138	1.957163
12	1	0	-1.048371	0.920816	1.472246
13	6	0	0.695788	0.513641	-1.299069
14	1	0	1.019969	0.219734	-2.300027
15	1	0	1.122732	1.496926	-1.089105
16	6	0	-0.856804	0.517469	-1.168703
17	1	0	-1.237606	1.502379	-0.889505
18	1	0	-1.343845	0.227645	-2.102699
19	7	0	-1.288882	-0.457599	-0.107289
20	7	0	1.294579	-0.460126	-0.320846
21	30	0	-11.899188	0.248034	0.141197
22	30	0	-3.516150	-0.497053	0.110467
23	30	0	-18.958484	0.462368	0.151098
24	7	0	-4.040612	-1.907841	-1.350013
25	7	0	-3.801548	0.896433	1.651280
26	7	0	-11.434105	1.078715	2.001324
27	7	0	-4.115478	0.996089	-1.229954
28	7	0	-11.562471	-0.619172	-1.731470
29	7	0	-11.545899	-1.649096	0.970947
30	7	0	-3.733592	-2.002720	1.539130
31	7	0	-19.355323	2.234052	1.188177
32	7	0	-19.297275	1.510124	-1.626774
33	7	0	-15.217728	9.623586	0.626002
34	6	0	-14.216933	10.267187	1.284476
35	6	0	-14.140141	11.654266	1.387199
36	1	0	-13.324418	12.107383	1.933006
37	6	0	-15.124486	12.437036	0.769011
38	6	0	-16.155896	11.793028	0.078484
39	1	0	-16.954794	12.314347	-0.429082
40	6	0	-16.165128	10.394437	0.043891
41	7	0	-11.408306	2.098501	-0.704664
42	7	0	-19.302268	-1.308982	-0.909835
43	7	0	-10.553395	-7.756287	-1.897191
44	1	0	-9.609897	-7.484943	-1.627019
45	7	0	-8.388243	-9.456173	-1.496625
46	6	0	-9.571992	-9.987261	-1.871302
47	6	0	-9.780794	-11.359818	-2.053287
48	1	0	-10.765641	-11.684082	-2.357442
49	6	0	-8.710458	-12.231674	-1.832248
50	6	0	-7.475595	-11.696861	-1.441662
51	1	0	-6.621194	-12.333270	-1.257980
52	6	0	-7.358273	-10.316762	-1.289552
53	6	0	-4.016249	-3.290737	-1.196924
54	7	0	-5.901843	-8.425057	-0.801537
55	1	0	-6.734323	-7.898813	-1.057401
56	7	0	-19.392118	-0.584099	1.906688
57	8	0	-11.843590	-9.598139	-2.483541
58	7	0	-13.105450	8.134762	1.671450
59	1	0	-13.876120	7.806632	1.100719
60	6	0	-3.752949	0.646267	3.018237
61	6	0	-11.359040	0.393347	3.209849
62	6	0	-3.737975	-1.850511	2.922193
63	8	0	-18.141375	10.469884	-1.293235
64	6	0	-3.869700	-3.981962	0.021366
65	8	0	-8.773147	-13.596522	-1.971394
66	6	0	-11.329752	0.038471	-2.933553
67	8	0	-12.251464	10.080027	2.618157
68	6	0	-11.370634	3.493612	1.365235
69	7	0	-17.401154	8.392794	-0.553753
70	1	0	-16.651766	7.969523	-0.017593
71	6	0	-11.385018	-1.007050	3.376043
72	6	0	-3.830440	2.278564	1.497481
73	6	0	-11.233016	2.376395	-2.056702
74	6	0	-3.823042	-3.158517	3.547757
75	1	0	-3.886453	-3.336674	4.608684
76	6	0	-19.481429	2.374745	2.564207
77	6	0	-19.255972	2.890781	-1.769363
78	8	0	-5.088433	-10.595430	-0.617477
79	6	0	-19.269879	0.972874	-2.907564
80	6	0	-11.495614	-1.943811	2.327984
81	6	0	-11.726595	-2.858314	0.306187
82	6	0	-3.613334	-0.694401	5.120326
83	6	0	-4.627426	-0.172145	5.946762
84	1	0	-5.499606	0.294130	5.501699
85	6	0	-4.522262	-0.244766	7.339910
86	1	0	-5.317294	0.161612	7.956933
87	6	0	-3.403847	-0.839674	7.934612
88	1	0	-3.324323	-0.895541	9.015634
89	6	0	-2.388119	-1.361767	7.125376
90	1	0	-1.514245	-1.821587	7.576568
91	6	0	-2.492311	-1.289744	5.732836
92	1	0	-1.699701	-1.691709	5.109796
93	6	0	-4.281681	0.840471	-2.601289
94	6	0	-11.376900	2.436599	2.302664
95	6	0	-4.044951	2.365251	-0.989723
96	6	0	-19.147820	3.223489	-3.179376
97	1	0	-19.070031	4.220663	-3.581273
98	6	0	-3.830196	4.474138	0.325066
99	6	0	-2.668654	5.114368	0.800155
100	1	0	-1.824949	4.512403	1.122634
101	6	0	-2.590232	6.509586	0.854530
102	1	0	-1.684662	6.983675	1.220179
103	6	0	-3.673717	7.291278	0.436958
104	1	0	-3.615276	8.374129	0.482034
105	6	0	-4.833634	6.667669	-0.036917
106	1	0	-5.681549	7.265301	-0.356282
107	6	0	-4.912529	5.272007	-0.094884
108	1	0	-5.817372	4.798106	-0.461504
109	6	0	-11.349182	3.316575	-0.035147
110	6	0	-3.708184	-0.627964	3.624211
111	6	0	-3.798668	-3.368619	1.288705
112	6	0	-12.107547	-4.381797	-1.648103
113	6	0	-13.304373	-4.582049	-2.363572
114	1	0	-13.994213	-3.754755	-2.490791
115	6	0	-13.602493	-5.838057	-2.894380
116	1	0	-14.532571	-5.987635	-3.433639
117	6	0	-12.722861	-6.912887	-2.749478
118	1	0	-12.958657	-7.884259	-3.155046
119	6	0	-11.515480	-6.725725	-2.049100
120	6	0	-11.227839	-5.464691	-1.491269
121	1	0	-10.309368	-5.331343	-0.932475
122	6	0	-3.851725	-4.087727	2.548018
123	1	0	-3.938113	-5.157657	2.647350
124	6	0	-10.754107	-9.091041	-2.112717
125	6	0	-3.770467	-5.480862	-0.016451
126	6	0	-4.859257	-6.284239	-0.385015
127	1	0	-5.807116	-5.823050	-0.632143
128	6	0	-4.752796	-7.688294	-0.421531
129	6	0	-3.529141	-8.296661	-0.078001
130	1	0	-3.443502	-9.371602	-0.103145
131	6	0	-2.448705	-7.493658	0.295609
132	1	0	-1.509115	-7.967728	0.561888
133	6	0	-2.555714	-6.102131	0.331119
134	1	0	-1.706387	-5.493324	0.622380
135	6	0	-4.378167	-0.383541	-3.294066
136	6	0	-19.463236	-0.044863	3.184556
137	6	0	-19.313978	3.833215	-0.721445
138	6	0	-4.264498	-1.657554	-2.698425
139	6	0	-11.654964	-3.375062	2.515598
140	1	0	-11.661040	-3.880308	3.467676
141	6	0	-4.134400	3.079372	-2.250073
142	1	0	-4.085548	4.149698	-2.364831
143	6	0	-11.812558	-3.019403	-1.090653
144	6	0	-19.394349	3.512694	0.648667
145	6	0	-11.253682	1.350942	4.295715
146	1	0	-11.162258	1.101224	5.340250
147	6	0	-19.462161	-2.579861	-0.371912
148	6	0	-19.159093	2.048365	-3.876259
149	6	0	-19.497529	1.326412	3.503971
150	6	0	-13.110365	9.479826	1.927167
151	6	0	-4.202808	-3.919709	-2.491437
152	1	0	-4.212151	-4.982561	-2.672114
153	6	0	-11.653630	-1.972968	-2.024953
154	6	0	-3.902392	2.976031	0.273569
155	6	0	-6.016149	-9.787076	-0.871321
156	6	0	-3.743571	1.908190	3.736272
157	1	0	-3.705818	2.017214	4.807565
158	6	0	-19.599667	1.694511	4.956799
159	6	0	-20.791136	2.228469	5.482985
160	1	0	-21.643760	2.367903	4.826166
161	6	0	-20.885509	2.568798	6.836758
162	1	0	-21.813732	2.975891	7.225621
163	6	0	-19.790856	2.380049	7.688649
164	1	0	-19.864326	2.644164	8.738856
165	6	0	-18.601334	1.847540	7.178218
166	1	0	-17.746687	1.700221	7.831306
167	6	0	-18.507325	1.507652	5.824244
168	1	0	-17.582267	1.098085	5.430579
169	6	0	-19.389273	-1.445147	-2.289736
170	6	0	-19.363595	5.282689	-1.101706
171	6	0	-18.364525	6.180783	-0.694148
172	1	0	-17.530918	5.813516	-0.101692
173	6	0	-18.430805	7.544998	-1.032555
174	6	0	-19.505809	8.019386	-1.807385
175	1	0	-19.555595	9.065181	-2.067419
176	6	0	-20.497420	7.123253	-2.213337
177	1	0	-21.331425	7.492865	-2.801356
178	6	0	-20.439899	5.772464	-1.867276
179	1	0	-21.226559	5.092095	-2.173182
180	6	0	-11.302946	-1.545996	4.775144
181	6	0	-12.338492	-1.312716	5.700464
182	1	0	-13.207265	-0.739819	5.391561
183	6	0	-12.262358	-1.817927	7.002528
184	1	0	-13.072994	-1.632572	7.700506
185	6	0	-11.148193	-2.565065	7.402356
186	1	0	-11.088196	-2.957085	8.412757
187	6	0	-10.113221	-2.804752	6.491059
188	1	0	-9.244333	-3.381048	6.793120
189	6	0	-10.187748	-2.302084	5.187154
190	1	0	-9.378927	-2.491444	4.487930
191	6	0	-11.268680	4.898380	1.887576
192	6	0	-10.154539	5.279140	2.660390
193	1	0	-9.375141	4.561025	2.880132
194	6	0	-10.037956	6.586656	3.131834
195	1	0	-9.173927	6.863720	3.726723
196	6	0	-11.002963	7.551275	2.839932
197	1	0	-10.905786	8.564582	3.195780
198	6	0	-12.112842	7.193815	2.050639
199	6	0	-12.243847	5.868079	1.592543
200	1	0	-13.105537	5.590163	0.991311
201	6	0	-11.181080	1.430078	-3.098771
202	6	0	-3.804291	2.907487	2.805671
203	1	0	-3.843261	3.969053	2.988137
204	6	0	-4.289107	2.146881	-3.235184
205	1	0	-4.384862	2.324634	-4.293775
206	6	0	-19.329422	-0.397518	-3.230907
207	6	0	-17.319657	9.751511	-0.671253
208	6	0	-11.290435	2.600441	3.743376
209	1	0	-11.249609	3.543093	4.263747
210	6	0	-11.253948	-0.937021	-4.005605
211	1	0	-11.052305	-0.710338	-5.040153
212	6	0	-4.378951	-2.920321	-3.407290
213	1	0	-4.573168	-3.029265	-4.461938
214	6	0	-10.915620	1.940334	-4.485663
215	6	0	-10.397786	2.889679	-7.092519
216	1	0	-10.199527	3.257091	-8.094527
217	6	0	-11.091046	3.809269	-2.236809
218	1	0	-10.939172	4.305379	-3.181356
219	6	0	-11.156482	4.384028	-1.000233
220	1	0	-11.047214	5.428593	-0.759700
221	6	0	-19.623768	-4.353768	1.361615
222	6	0	-20.791120	-4.852301	1.971309
223	1	0	-21.613207	-4.173623	2.174283
224	6	0	-20.902355	-6.206836	2.302577
225	1	0	-21.812332	-6.573815	2.767159
226	6	0	-19.848942	-7.087747	2.030388
227	1	0	-19.935510	-8.138636	2.287730
228	6	0	-18.683996	-6.605638	1.422385
229	1	0	-17.861079	-7.280863	1.209499
230	6	0	-18.573552	-5.251258	1.090014
231	1	0	-17.667114	-4.880809	0.621809
232	6	0	-19.477649	-1.963622	2.049096
233	6	0	-19.502594	-2.902321	0.999508
234	6	0	-19.595579	3.784057	2.894541
235	1	0	-19.707067	4.182997	3.889859
236	6	0	-19.566343	-1.119855	4.155207
237	1	0	-19.645456	-0.988073	5.222363
238	6	0	-19.557167	4.480382	1.720494
239	1	0	-19.649042	5.546349	1.589362
240	6	0	-11.812349	-3.933042	1.278478
241	1	0	-11.975150	-4.973036	1.045785
242	6	0	-11.471453	-2.168381	-3.451822
243	1	0	-11.474267	-3.123020	-3.953444
244	6	0	-19.564385	-2.295801	3.460479
245	1	0	-19.628796	-3.294064	3.862105
246	6	0	-4.645593	-0.320188	-4.768664
247	6	0	-3.723490	-0.827965	-5.703955
248	1	0	-2.792870	-1.260791	-5.350970
249	6	0	-3.990072	-0.770612	-7.076105
250	1	0	-3.264424	-1.163039	-7.781747
251	6	0	-5.183768	-0.204823	-7.539283
252	1	0	-5.390627	-0.163052	-8.603928
253	6	0	-6.107479	0.308449	-6.621020
254	1	0	-7.037554	0.748920	-6.967014
255	6	0	-5.840058	0.253395	-5.249278
256	1	0	-6.555633	0.659662	-4.541690
257	6	0	-19.652640	-3.536679	-1.447309
258	6	0	-19.608954	-2.841602	-2.622022
259	6	0	-19.362324	-0.769916	-4.683158
260	6	0	-18.324638	-1.530049	-5.256625
261	1	0	-17.490478	-1.840660	-4.635512
262	6	0	-18.355168	-1.879974	-6.610569
263	1	0	-17.542964	-2.462291	-7.034708
264	6	0	-19.426299	-1.478128	-7.417105
265	1	0	-19.451281	-1.750548	-8.467464
266	6	0	-20.466229	-0.724743	-6.859723
267	1	0	-21.304733	-0.415393	-7.475943
268	6	0	-20.434614	-0.373686	-5.506141
269	1	0	-21.247424	0.201253	-5.074697
270	6	0	-10.035048	-14.229599	-2.377874
271	1	0	-10.816815	-13.976913	-1.648614
272	1	0	-10.327818	-13.840635	-3.361388
273	6	0	-9.799457	-15.740146	-2.427989
274	1	0	-8.962157	-15.911093	-3.119480
275	6	0	-9.413507	-16.298251	-1.046456
276	1	0	-9.200133	-17.371353	-1.109718
277	1	0	-10.233315	-16.160476	-0.328551
278	1	0	-8.526895	-15.791969	-0.654884
279	6	0	-11.053407	-16.436926	-2.992910
280	1	0	-10.889286	-17.517372	-3.065916
281	1	0	-11.307467	-16.067010	-3.993604
282	1	0	-11.921496	-16.276103	-2.340380
283	7	0	-14.126534	0.330870	0.172396
284	6	0	-14.637449	0.479433	-1.235269
285	1	0	-14.197053	1.390102	-1.649072
286	1	0	-14.256978	-0.364659	-1.813872
287	7	0	-16.714201	0.438789	0.171564
288	6	0	-16.192736	0.532625	-1.235807
289	1	0	-16.628374	-0.293960	-1.801312
290	1	0	-16.566955	1.463196	-1.666976
291	6	0	-14.596636	1.507886	0.983944
292	1	0	-14.199990	1.390582	1.994567
293	1	0	-14.145953	2.404599	0.552278
294	6	0	-16.153157	1.578053	0.975043
295	1	0	-16.517308	2.506688	0.529686
296	1	0	-16.569706	1.510334	1.982682
297	6	0	-14.698228	-0.928321	0.768182
298	1	0	-14.334045	-1.769550	0.175046
299	1	0	-14.289400	-1.033341	1.775681
300	6	0	-16.252324	-0.857163	0.777300
301	1	0	-16.657207	-0.909812	1.790035
302	1	0	-16.700586	-1.667483	0.198418
303	6	0	-11.893955	1.846326	-5.493226
304	1	0	-12.859099	1.409168	-5.257317
305	6	0	-11.637489	2.316844	-6.785820
306	1	0	-12.406106	2.240907	-7.548712
307	6	0	-9.417054	2.990191	-6.097810
308	1	0	-8.453888	3.436509	-6.326212
309	6	0	-9.671294	2.520117	-4.804213
310	1	0	-8.908503	2.592649	-4.035606
311	8	0	-14.995783	13.796372	0.890961
312	6	0	-15.589238	16.124432	0.589115
313	1	0	-14.562335	16.261965	0.221738
314	6	0	-15.989156	14.683811	0.266957
315	1	0	-15.993681	14.504818	-0.815572
316	1	0	-16.983399	14.450724	0.671441
317	6	0	-16.516470	17.095251	-0.169241

318	1	0	-16.476559	16.932239	-1.252990
319	1	0	-16.223159	18.132351	0.025233
320	1	0	-17.559025	16.978753	0.154206
321	6	0	-15.605849	16.390677	2.105013
322	1	0	-14.948928	15.692979	2.631815
323	1	0	-16.621160	16.278612	2.508668
324	1	0	-15.270459	17.411414	2.320619
325	8	0	-7.731513	-2.707562	2.664567
326	8	0	-7.655716	1.545175	-2.081407
327	7	0	-7.682928	2.192406	-0.997774
328	8	0	-7.980978	-5.996291	-1.510673
329	6	0	-7.715074	-1.943510	1.686427
330	6	0	-7.780072	-2.300638	0.255093
331	6	0	-7.777838	-1.120770	-0.541627
332	6	0	-7.861254	-1.253539	-1.931994
333	1	0	-7.884868	-0.393556	-2.575195
334	6	0	-7.917494	-2.534441	-2.496152
335	1	0	-7.974309	-2.655562	-3.569098
336	6	0	-7.899064	-3.667313	-1.678369
337	6	0	-7.840957	-3.570595	-0.280829
338	1	0	-7.841221	-4.447180	0.352905
339	8	0	-7.221489	2.015110	4.973288
340	8	0	-7.963268	-5.080274	-3.544891
341	8	0	-7.756709	3.453073	-0.958545
342	8	0	-7.280710	3.815837	3.658952
343	7	0	-7.948389	-4.980958	-2.291657
344	7	0	-7.312357	2.564406	3.838374
345	6	0	-7.639420	-0.460076	1.715245
346	6	0	-7.532976	0.315613	2.851035
347	1	0	-7.509824	-0.131391	3.835808
348	6	0	-7.457324	1.699797	2.671260
349	6	0	-7.512167	2.271996	1.408498
350	1	0	-7.462016	3.344622	1.293331
351	6	0	-7.628986	1.459662	0.269180
352	6	0	-7.681094	0.055235	0.386281
353	1	0	-19.090120	1.920607	-4.944480
354	1	0	-19.746334	-3.229247	-3.618547
355	1	0	-19.829394	-4.591951	-1.315298
356	30	0	11.906048	0.229986	-0.114666
357	30	0	3.527379	-0.502650	-0.484245
358	30	0	18.957106	0.446942	0.224697
359	7	0	3.968222	-1.886667	1.029705
360	7	0	3.890212	0.863977	-2.032343
361	7	0	11.537032	1.024490	-2.012124
362	7	0	4.054489	1.014245	0.858816
363	7	0	11.473320	-0.600469	1.754836
364	7	0	11.592357	-1.682800	-0.924102
365	7	0	3.829662	-2.031944	-1.870299
366	7	0	19.404004	2.203399	-0.818188
367	7	0	19.208054	1.522053	2.001047
368	7	0	15.248109	9.594257	-0.588907
369	6	0	14.284316	10.225878	-1.311084
370	6	0	14.212001	11.611073	-1.439226
371	1	0	13.427063	12.054354	-2.035884
372	6	0	15.160175	12.404966	-0.780034
373	6	0	16.152902	11.773452	-0.024300
374	1	0	16.922434	12.303909	0.518002
375	6	0	16.161597	10.375573	0.032130
376	7	0	11.373813	2.096758	0.668341
377	7	0	19.249663	-1.307550	1.327780
378	7	0	10.441453	-7.733082	1.998086
379	1	0	9.513712	-7.465478	1.674758
380	7	0	8.297402	-9.438158	1.514164
381	6	0	9.459812	-9.963772	1.957117
382	6	0	9.657394	-11.333446	2.170650
383	1	0	10.624889	-11.653346	2.530094
384	6	0	8.598829	-12.208226	1.908275
385	6	0	7.386181	-11.679075	1.446755
386	1	0	6.541523	-12.317943	1.229580
387	6	0	7.278335	-10.301425	1.267918
388	6	0	3.951680	-3.271952	0.900016
389	7	0	5.851254	-8.416438	0.678603
390	1	0	6.671470	-7.887221	0.966056
391	7	0	19.475592	-0.625229	-1.491701
392	8	0	11.697005	-9.565722	2.680474
393	7	0	13.196850	8.086029	-1.723917
394	1	0	13.935013	7.768338	-1.106245
395	6	0	3.906310	0.589329	-3.395231
396	6	0	11.523241	0.315598	-3.209252
397	6	0	3.893066	-1.904955	-3.254115
398	8	0	18.062824	10.474313	1.472678
399	6	0	3.872335	-3.984061	-0.313137
400	8	0	8.652727	-13.570786	2.071440
401	6	0	11.179638	0.080692	2.930236
402	8	0	12.395692	10.014378	-2.748234
403	6	0	11.444383	3.451325	-1.427581
404	7	0	17.365112	8.385236	0.725534
405	1	0	16.645690	7.952946	0.156516
406	6	0	11.555301	-1.087762	-3.346419
407	6	0	3.914029	2.248436	-1.902315
408	6	0	11.129348	2.400959	2.003882
409	6	0	4.001718	-3.224389	-3.851655
410	1	0	4.110233	-3.421932	-4.905488
411	6	0	19.598325	2.323383	-2.188225
412	6	0	19.158659	2.904638	2.120538
413	8	0	5.044145	-10.588579	0.488003
414	6	0	19.117365	1.004095	3.286826
415	6	0	11.610714	-2.003852	-2.276005
416	6	0	11.736844	-2.879022	-0.227727
417	6	0	3.859456	-0.789630	-5.476482
418	6	0	4.910156	-0.286950	-6.268495
419	1	0	5.765056	0.183873	-5.795430
420	6	0	4.863422	-0.384843	-7.663317
421	1	0	5.685713	0.006709	-8.253635
422	6	0	3.768012	-0.985805	-8.293706
423	1	0	3.733794	-1.061141	-9.375931
424	6	0	2.716367	-1.488534	-7.518782
425	1	0	1.859848	-1.952570	-7.998046
426	6	0	2.762183	-1.391351	-6.124598
427	1	0	1.942086	-1.778008	-5.528024
428	6	0	4.161682	0.883243	2.238704
429	6	0	11.498032	2.376210	-2.342623
430	6	0	3.991366	2.378777	0.591008
431	6	0	18.980240	3.258343	3.518252
432	1	0	18.881812	4.261317	3.900718
433	6	0	3.852868	4.464612	-0.770216

434	6	0	2.721128	5.099121	-1.319147
435	1	0	1.894196	4.493771	-1.676911
436	6	0	2.650255	6.493335	-1.402111
437	1	0	1.767515	6.962977	-1.824883
438	6	0	3.711851	7.279843	-0.939793
439	1	0	3.659434	8.361858	-1.006944
440	6	0	4.842216	6.662031	-0.392616
441	1	0	5.673468	7.263250	-0.038115
442	6	0	4.913304	5.267437	-0.306023
443	1	0	5.795515	4.797943	0.117015
444	6	0	11.349791	3.301450	-0.027029
445	6	0	3.890555	-0.695851	-3.979079
446	6	0	3.875742	-3.393188	-1.592409
447	6	0	12.013134	-4.364934	1.772545
448	6	0	13.168828	-4.553098	2.555715
449	1	0	13.852215	-3.724194	2.705261
450	6	0	13.435383	-5.799544	3.124560
451	1	0	14.334505	-5.940055	3.716216
452	6	0	12.563080	-6.876182	2.951377
453	1	0	12.774915	-7.840285	3.386458
454	6	0	11.395833	-6.700748	2.183234
455	6	0	11.141267	-5.449830	1.587928
456	1	0	10.254847	-5.325847	0.977641
457	6	0	3.983372	-4.135288	-2.834813
458	1	0	4.069266	-5.207183	-2.910897
459	6	0	10.628934	-9.064261	2.245291
460	6	0	3.766771	-5.482099	-0.254021
461	6	0	4.835334	-6.281291	0.176923
462	1	0	5.772474	-5.818025	0.458467
463	6	0	4.723058	-7.684250	0.233129
464	6	0	3.514339	-8.295690	-0.154860
465	1	0	3.424516	-9.369875	-0.115431
466	6	0	2.454256	-7.496789	-0.590573
467	1	0	1.526321	-7.973408	-0.891031
468	6	0	2.566858	-6.106227	-0.645061
469	1	0	1.733904	-5.500240	-0.985371
470	6	0	4.232225	-0.327600	2.957311
471	6	0	19.608994	-0.105207	-2.772537
472	6	0	19.268039	3.831232	1.062632
473	6	0	4.139694	-1.612542	2.381524
474	6	0	11.777138	-3.438551	-2.427509
475	1	0	11.831146	-3.962092	-3.368117
476	6	0	4.017731	3.114920	1.841499
477	1	0	3.958644	4.186788	1.935213
478	6	0	11.751080	-3.013095	1.174572
479	6	0	19.416425	3.490065	-0.296785
480	6	0	11.476501	1.251696	-4.317722
481	1	0	11.439182	0.981451	-5.360487
482	6	0	19.436636	-2.586145	0.817554
483	6	0	18.957726	2.093846	4.232489
484	1	0	18.835850	1.982085	5.297778
485	6	0	19.659973	1.261123	-3.110256
486	6	0	13.215365	9.426713	-2.001124
487	6	0	4.083851	-3.878137	2.212034
488	1	0	4.086133	-4.937705	2.411392
489	6	0	11.546664	-1.948367	2.078952
490	6	0	3.918617	2.967416	-0.688887
491	6	0	5.959579	-9.777320	0.774956
492	6	0	3.936144	1.838238	-4.135579
493	1	0	3.951225	1.927805	-5.209237
494	6	0	19.834423	1.607508	-4.561582
495	6	0	21.052216	2.130252	-5.036224
496	1	0	21.871979	2.277078	-4.340335
497	6	0	21.214293	2.450131	-6.388572
498	1	0	22.161937	2.848838	-6.737193
499	6	0	20.162240	2.251730	-7.290474
500	1	0	20.288243	2.499939	-8.339571
501	6	0	18.947150	1.730269	-6.831465
502	1	0	18.125160	1.575646	-7.523614
503	6	0	18.785410	1.410876	-5.478884
504	1	0	17.840614	1.009884	-5.125295
505	6	0	19.269119	-1.422782	2.712180
506	6	0	19.297679	5.286385	1.422592
507	6	0	18.320325	6.176798	0.950626
508	1	0	17.518204	5.799196	0.322301
509	6	0	18.368846	7.546261	1.270273
510	6	0	19.402731	8.034026	2.091379
511	1	0	19.438768	9.083841	2.337118
512	6	0	20.372666	7.145526	2.561436
513	1	0	21.175473	7.525311	3.185302
514	6	0	20.333397	5.789396	2.234194
515	1	0	21.103747	5.114869	2.590404
516	6	0	11.544421	-1.654022	-4.737113
517	6	0	12.627512	-1.441104	-5.611502
518	1	0	13.480655	-0.863858	-5.268969
519	6	0	12.617995	-1.971933	-6.905542
520	1	0	13.464519	-1.802036	-7.563713
521	6	0	11.524029	-2.724739	-7.348220
522	1	0	11.515711	-3.136649	-8.352434
523	6	0	10.442169	-2.944250	-6.487714
524	1	0	9.588508	-3.524712	-6.823342
525	6	0	10.450014	-2.415936	-5.191860
526	1	0	9.605124	-2.589858	-4.532425
527	6	0	11.372270	4.845849	-1.981295
528	6	0	10.302115	5.212288	-2.820369
529	1	0	9.534483	4.490583	-3.067937
530	6	0	10.212868	6.510783	-3.321814
531	1	0	9.382695	6.776808	-3.967760
532	6	0	11.161536	7.480665	-2.995722
533	1	0	11.084852	8.487213	-3.375012
534	6	0	12.226175	7.137992	-2.140332
535	6	0	12.330910	5.820897	-1.651675
536	1	0	13.158230	5.554210	-0.999267
537	6	0	11.023812	1.475233	3.060269
538	6	0	3.955502	2.853798	-3.221186
539	1	0	4.006748	3.911833	-3.420439
540	6	0	4.133320	2.200403	2.848990
541	1	0	4.180069	2.397149	3.907529
542	6	0	19.161949	-0.361231	3.633341
543	6	0	17.276335	9.745414	0.818255
544	6	0	11.487078	2.511784	-3.788828
545	1	0	11.475498	3.444045	-4.328998
546	6	0	11.046820	-0.873587	4.015702
547	1	0	10.792058	-0.626599	5.033754
548	6	0	4.224607	-2.862801	3.116341
549	1	0	4.376888	-2.953367	4.179588

550	6	0	10.688701	2.012785	4.421492
551	6	0	10.040900	3.015149	6.979054
552	1	0	9.792746	3.403087	7.962025
553	6	0	10.978473	3.836956	2.148304
554	1	0	10.778220	4.351465	3.073784
555	6	0	11.107534	4.387422	0.905704
556	1	0	11.010762	5.426948	0.639404
557	6	0	19.682787	-4.385886	-0.879033
558	6	0	20.878513	-4.891787	-1.424251
559	1	0	21.708843	-4.214982	-1.597773
560	6	0	21.006705	-6.251027	-1.729061
561	1	0	21.938277	-6.623588	-2.143715
562	6	0	19.942187	-7.129370	-1.494365
563	1	0	20.041933	-8.183918	-1.731150
564	6	0	18.748943	-6.639914	-0.950492
565	1	0	17.917325	-7.313134	-0.767111
566	6	0	18.621480	-5.280805	-0.644565
567	1	0	17.693269	-4.904711	-0.226161
568	6	0	19.567995	-2.006685	-1.608998
569	6	0	19.543166	-2.929323	-0.545266
570	6	0	19.729476	3.727539	-2.533707
571	1	0	19.890645	4.111395	-3.528153
572	6	0	19.758519	-1.194579	-3.720821
573	1	0	19.889080	-1.078747	-4.784772
574	6	0	19.632764	4.441516	-1.373706
575	1	0	19.718478	5.509373	-1.254320
576	6	0	11.870390	-3.972466	-1.173411
577	1	0	12.019805	-5.007799	-0.912709
578	6	0	11.290479	-2.115712	3.498003
579	1	0	11.265409	-3.060373	4.017555
580	6	0	19.722680	-2.359971	-3.009420
581	1	0	19.806113	-3.364113	-3.392451
582	6	0	4.441964	-0.236267	4.439670
583	6	0	3.486920	-0.731655	5.348243
584	1	0	2.572173	-1.175257	4.968245
585	6	0	3.700231	-0.647837	6.728290
586	1	0	2.949550	-1.030721	7.412657
587	6	0	4.872703	-0.067635	7.226459
588	1	0	5.038258	-0.005407	8.297311
589	6	0	5.828629	0.433752	6.335002
590	1	0	6.742716	0.885343	6.708168
591	6	0	5.614343	0.352490	4.955267
592	1	0	6.354532	0.749897	4.268331
593	6	0	19.575454	-3.526296	1.915326
594	1	0	19.759452	-4.583134	1.808089
595	6	0	19.473731	-2.813688	3.075853
596	1	0	19.562674	-3.186065	4.083630
597	6	0	19.123625	-0.711670	5.090890
598	6	0	18.060169	-1.465635	5.623890
599	1	0	17.258266	-1.787689	4.967146
600	6	0	18.024310	-1.794968	6.982857
601	1	0	17.193122	-2.372804	7.375149
602	6	0	19.053548	-1.378330	7.835139
603	1	0	19.027021	-1.634772	8.889475
604	6	0	20.118464	-0.630885	7.318430
605	1	0	20.924939	-0.310184	7.970551
606	6	0	20.153222	-0.300410	5.959759
607	1	0	20.985379	0.269958	5.560322
608	6	0	9.890814	-14.197775	2.553587
609	1	0	10.710090	-13.957069	1.862449
610	1	0	10.132021	-13.793236	3.544786
611	6	0	9.651080	-15.707238	2.615323
612	1	0	8.778021	-15.866729	3.263998
613	6	0	9.338397	-16.287352	1.224370
614	1	0	9.120190	-17.359126	1.293442
615	1	0	10.195511	-16.161855	0.549051
616	1	0	8.474713	-15.786829	0.777964
617	6	0	10.872395	-16.395282	3.257196
618	1	0	10.703584	-17.474445	3.338040
619	1	0	11.072956	-16.009895	4.264183
620	1	0	11.774285	-16.244695	2.649607
621	7	0	14.132038	0.312142	-0.034715
622	6	0	14.570922	0.503562	1.391980
623	1	0	14.111755	1.426681	1.754592
624	1	0	14.159867	-0.322317	1.975934
625	7	0	16.716610	0.420769	0.094358
626	6	0	16.124339	0.555944	1.470094
627	1	0	16.528747	-0.254211	2.080780
628	1	0	16.478337	1.498227	1.892888
629	6	0	14.644323	1.464291	-0.856364
630	1	0	14.299393	1.316636	-1.881870
631	1	0	14.173398	2.373459	-0.475317
632	6	0	16.198365	1.535327	-0.770250
633	1	0	16.540378	2.477084	-0.335063
634	1	0	16.665557	1.437549	-1.752910
635	6	0	14.731826	-0.964444	-0.561933
636	1	0	14.337657	-1.787128	0.038041
637	1	0	14.373914	-1.101047	-1.584888
638	6	0	16.284461	-0.892656	-0.495433
639	1	0	16.739281	-0.974518	-1.484756
640	1	0	16.702614	-1.685428	0.128503
641	6	0	11.614417	1.938434	5.479188
642	1	0	12.589596	1.496047	5.301488
643	6	0	11.293479	2.435143	6.747352
644	1	0	12.022233	2.374042	7.549677
645	6	0	9.112100	3.096266	5.933962
646	1	0	8.139473	3.548065	6.104015
647	6	0	9.430785	2.599705	4.664727
648	1	0	8.708097	2.657304	3.857071
649	8	0	15.036943	13.762038	-0.929636
650	6	0	15.610536	16.095542	-0.631446
651	1	0	14.564726	16.236823	-0.323669
652	6	0	15.993445	14.660762	-0.265474
653	1	0	15.938276	14.498392	0.818279
654	1	0	17.008758	14.423329	-0.610753
655	6	0	16.493425	17.079571	0.162000
656	1	0	16.393610	16.933254	1.244269
657	1	0	16.210449	18.113031	-0.064519
658	1	0	17.552418	16.959894	-0.101308
659	6	0	15.710930	16.338495	-2.148024
660	1	0	15.084748	15.631748	-2.699613
661	1	0	16.747161	16.221795	-2.492891
662	1	0	15.387305	17.355275	-2.397614
663	8	0	7.853207	-2.765210	-2.798304
664	8	0	7.553789	1.570166	1.863009
665	7	0	7.634045	2.198492	0.770892

666	8	0	7.899278	-5.979966	1.441680
667	6	0	7.791541	-1.984057	-1.835634
668	6	0	7.786781	-2.315971	-0.396753
669	6	0	7.749528	-1.122171	0.378063
670	6	0	7.768191	-1.230429	1.772922
671	1	0	7.764318	-0.359481	2.401547
672	6	0	7.795256	-2.501216	2.361575
673	1	0	7.802029	-2.603319	3.437958
674	6	0	7.812391	-3.648360	1.563930
675	6	0	7.820097	-3.576344	0.163708
676	1	0	7.848976	-4.463959	-0.453804
677	8	0	7.471068	1.916661	-5.211585
678	8	0	7.784371	-5.027951	3.456106
679	8	0	7.707741	3.458447	0.713336
680	8	0	7.473100	3.740444	-3.927973
681	7	0	7.830196	-4.950850	2.202067
682	7	0	7.508480	2.485923	-4.083536
683	6	0	7.721444	-0.501175	-1.894084
684	6	0	7.673141	0.254583	-3.047142
685	1	0	7.697034	-0.209671	-4.023896
686	6	0	7.592715	1.641853	-2.895576
687	6	0	7.585960	2.236126	-1.641899
688	1	0	7.532735	3.310692	-1.548056
689	6	0	7.643955	1.443721	-0.484132
690	6	0	7.699523	0.037396	-0.573939

----

6 References

(S1) Hisano, N.; Haino, T., *J. Org. Chem.* **2022**, *87*, 4001-4009.

(S2) Baptiste, B.; Zhu, J.; Haldar, D.; Kauffmann, B.; Léger, J.; Huc, I., *Chem. Asian J.* **2010**, *5*, 1364-1375.

(S3) Faour, L.; Adam, C.; Gautier, C.; Goeb, S.; Allain, M.; Levillain, E.; Canevet, D.; Sallé, M., *Chem. Commun.* **2019**, *55*, 5743-5746.

(S4) Hisano, N.; Kodama, T.; Haino, T., *Chem. Eur. J.* **2023**, *29*, e202300107.

(S5) Pavelyev, V. G.; Parashchuk, O. D.; Krompiec, M.; Orekhova, T. V.; Perepichka, I. F.; van Loosdrecht, P. H. M.; Paraschuk, D. Y.; Pshenichnikov, M. S. J., *Phys. Chem. C* **2014**, *118*, 30291– 30301.

(S6) Ousaka, N.; Yamamoto, S.; Iida, H.; Iwata, T.; Ito, S.; Hijikata, Y.; Irle, S.; Yashima, E., *Nat. Commun.* **2019**, *10*, 1457.

(S7) S. Akine, TitrationFit program for analyses of host–guest complexation, Kanazawa University, Kanazawa, Japan, 2013.

(S8) Connrs, K. A., *Binding Constants: The Measurement of Molecular Complex Stability*. Wiley-Interscience: New York, 1987.

(S9) Gaussian 16, Revision C.01, Frisch, M. J.; Trucks, G. W.; Schlegel, H. B.; Scuseria, G. E.; Robb, M. A.; Cheeseman, J. R.; Scalmani, G.; Barone, V.; Petersson, G. A.; Nakatsuji, H.; Li, X.; Caricato, M.; Marenich, A. V.; Bloino, J.; Janesko, B. G.; Gomperts, R.; Mennucci, B.; Hratchian, H. P.; Ortiz, J. V.; Izmaylov, A. F.; Sonnenberg, J. L.; Williams-Young, D.; Ding, F.; Lipparini, F.; Egidi, F.; Goings, J.; Peng, B.; Petrone, A.; Henderson, T.; Ranasinghe, D.; Zakrzewski, V. G.; Gao, J.; Rega, N.; Zheng, G.; Liang, W.; Hada, M.; Ehara, M.; Toyota, K.; Fukuda, R.; Hasegawa, J.; Ishida, M.; Nakajima, T.; Honda, Y.; Kitao, O.; Nakai, H.; Vreven, T.; Throssell, K.; Montgomery, J. A., Jr.; Peralta, J. E.; Ogliaro, F.; Bearpark, M. J.; Heyd, J. J.; Brothers, E. N.; Kudin, K. N.; Staroverov, V. N.; Keith, T. A.; Kobayashi, R.; Normand, J.; Raghavachari, K.; Rendell, A. P.; Burant, J. C.; Iyengar, S. S.; Tomasi, J.; Cossi, M.; Millam, J. M.; Klene, M.; Adamo, C.; Cammi, R.; Ochterski, J. W.; Martin, R. L.; Morokuma, K.; Farkas, O.; Foresman, J. B.; Fox, D. J. Gaussian, Inc., Wallingford CT, 2016.
